# Tetracycline Antibiotics Induce Biosynthesis of Pro-Inflammatory Metabolites in the Immunobiotic *Bacteroides dorei*

**DOI:** 10.1021/acscentsci.5c00969

**Published:** 2025-12-03

**Authors:** Esther J. Han, Jack G. Ganley, Caitlin B. Winner, Joon Soo An, Mohammad R. Seyedsayamdost

**Affiliations:** † Department of Chemistry, 6740 Princeton University, Princeton, New Jersey 08544, United States; ‡ Department of Molecular Biology, Princeton University, Princeton, New Jersey 08544, United States

## Abstract

The human gut microbiome consists of diverse microbes that communicate through small molecules. Numerous recent studies have demonstrated links between gut microbiota and host physiological processes; however, the underlying metabolites remain elusive in part because laboratory conditions do not replicate the native environment of these bacteria. Herein, we focused on *Bacteroides dorei*, a predominant and representative member of human gut microbiota, to interrogate the chemical composition and possible biological functions of its secondary metabolome. Using UPLC-MS-guided high-throughput elicitor screening (HiTES), we examined how the metabolome of this commensal bacterium responds to hundreds of FDA-approved drug molecules that the host may intake. We identified low-dose tetracyclines as pleiotropic inducers of the *B. dorei* secondary metabolome, leading to the identification and structural elucidation of six serine-glycine dipeptide lipids, named doreamides A–F, and two 6-*N*-acyladenosines. The induced doreamides and *N*-acyladenosines exhibited pro-inflammatory activities, upregulating tumor necrosis factor α (TNFα), interleukin (IL)-1β, IL-6, and IL-10 in macrophages. Doreamides also triggered production of cathelicidin, which inhibits the growth of multiple bacteria tested but not *B. dorei*. Our results show that low-dose antibiotics can perturb the secondary metabolome of gut bacteria, and that these induced metabolites can exert immunomodulatory effects and restructure the microbiome.

## Graphical abstract

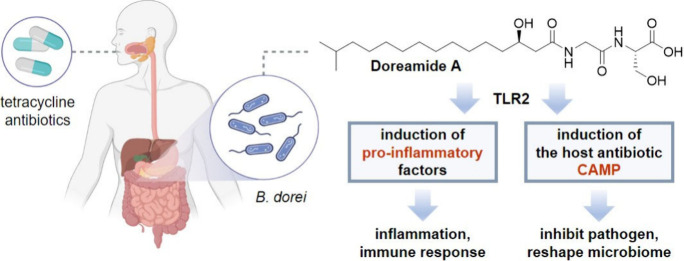

## Introduction

The human gut microbiome consists of a diverse and dynamic community of microorganisms that shapes host health. Among the multitude of microbial inhabitants, *Bacteroides* comprise 25% of human gut microbiota and represent one of the most abundant anaerobes.^1^ *Bacteroides* spp. are Gram-negative, bile-resistant, and nonspore-forming bacteria and have recently gained attention for their multifunctional roles, including regulation of metabolism and immunomodulatory activities.^1–5^ *Bacteroides thetaiotaomicron*, for example, has emerged as a model system and encodes, among other functions, carbohydrate-degrading enzymes that provide nutrients for the host and symbiotic gut microbes.^2,3^ Moreover, *Bacteroides uniformis* and *Bacteroides vulgatus* can attenuate colitis phenotypes in animal models, highlighting potentially beneficial contributions to host physiology.^4,5^ While numerous studies have identified statistically robust correlations between altered gut microbiome composition and disease progression, the molecular details underlying many of these correlations remain to be elucidated.

Bacteria communicate and compete with other microbes using a complex chemical language consisting of secreted small molecules, synonymously referred to as secondary metabolites or natural products. Initial investigations into microbiome secondary metabolites have revealed significant structural diversity and function, including nonribosomal peptides and thiopeptide natural products with potent antimicrobial activity^6,7^ and *N*-acyl amides that mimic eukaryotic signaling molecules.^8^ Additionally, pro-inflammatory polysaccharides associated with Crohn’s Disease,^9^ phospholipids that induce a homeostatic immune response,^10^ bacterial fatty acid amides that promote exercise,^11,12^ and structurally ornate metabolites synthesized by metalloenzymes in gut clostridia or oral streptococci have been characterized.^13–15^ Another important example is colibactin, a genotoxic metabolite produced by certain *Escherichia coli* strains,^16^ although it has yet to be isolated as a pure natural product. These accomplishments notwithstanding, a recent analysis of the human microbiome revealed over 10,000 recognizable biosynthetic gene clusters (BGCs) that code for secondary metabolites.^17^ Thus, the products of the vast majority of natural product biosynthetic pathways in the human microbiome remain unexplored, providing ample opportunities to potentially link host health states to specific bacterially derived metabolites.^18,19^

A challenge in examining the chemistry and biology of these microbiome metabolites is that the corresponding BGCs may not be constitutively active, as they are only expressed under a specific set of conditions. A similar phenomenon has been observed in soil-derived actinomycetes.^20,21^ To address these challenges, we have leveraged high-throughput elicitor screening (HiTES), a forward chemical genetics strategy for identifying small molecule modulators of silent or sparingly expressed BGCs.^21–24^ In HiTES, a microorganism is subjected to a library of small molecules and the secondary metabolome is then examined using a variety of read-outs, including genetic reporters, biological activity, or mass spectrometry (MS)-based methods. HiTES has enabled the discovery of over a hundred novel ‘cryptic’ metabolites, that is, molecules that are not observed under standard laboratory growth conditions.^24–31^ Moreover, the approach has uncovered the regulatory circuits involved in activation of transcriptionally silent BGCs.^32–34^

Herein, we have applied HiTES to the important gut microbiome member *Bacteroides dorei*, a species that is part of the normal gut flora in healthy individuals and contributes to the overall balance of the intestinal microbial community.^35–37^ To explore its metabolic capacity in response to exogenous cues, we screened a library of FDA-approved drugs and found subinhibitory doses of tetracycline antibiotics as strong inducers of a series of serine-glycine dipeptide lipids, termed doreamides, as well as 6-*N*-acyladenosines. Both sets of compounds are produced in limited quantities under unstimulated conditions and were structurally characterized using spectroscopic methods upon elicitation with tetracyclines. Investigations into their biological functions revealed induction of pro-inflammatory cytokines in macrophages, notably tumor necrosis factor α (TNFα), interleukin (IL)-1β, IL-6, and IL-10. Additionally and importantly, the doreamides elicited production of cathelicidin, a host-derived antimicrobial peptide that inhibited the growth of several gut bacteria tested but did not affect *B. dorei*. Phylogenetic analysis showed that the genes coding for the dipeptide lipids are highly conserved in and restricted to the Bacteroidota phylum. Our findings reveal new, induced microbiome metabolites and suggest mechanisms of interplay between *Bacteroides* spp. and human host cells.

## Results and Discussion

### UPLC-Guided HiTES in *B. dorei*

To explore the secondary metabolome of *B. dorei*, we challenged the strain with a collection of 400 structurally and functionally diverse, FDA-approved small molecule drugs (Figures 1A, S1; Table S1). After incubation and growth in a 96-well plate format, the cell-free supernatants were subjected to UPLC-qTOF-MS analysis using established methods and the data analyzed via the Metabolomics Explorer (MetEx), an in-house software tailored for analyzing multidimensional data sets.^38^ The intensity of detected ions in each well was subtracted from those observed in the absence of elicitors, thus focusing on induced metabolites. The results are depicted in a three-dimensional plot, which shows the *m*/*z* and intensity of induced metabolites as a function of the elicitor library (Figure 1A). The plot shows a number of elicited compounds, particularly in the *m*/*z* range of 300–500, suggesting that the secondary metabolome of *B. dorei* is responsive to the library of FDA-approved small molecule drugs.

**Figure 1:**
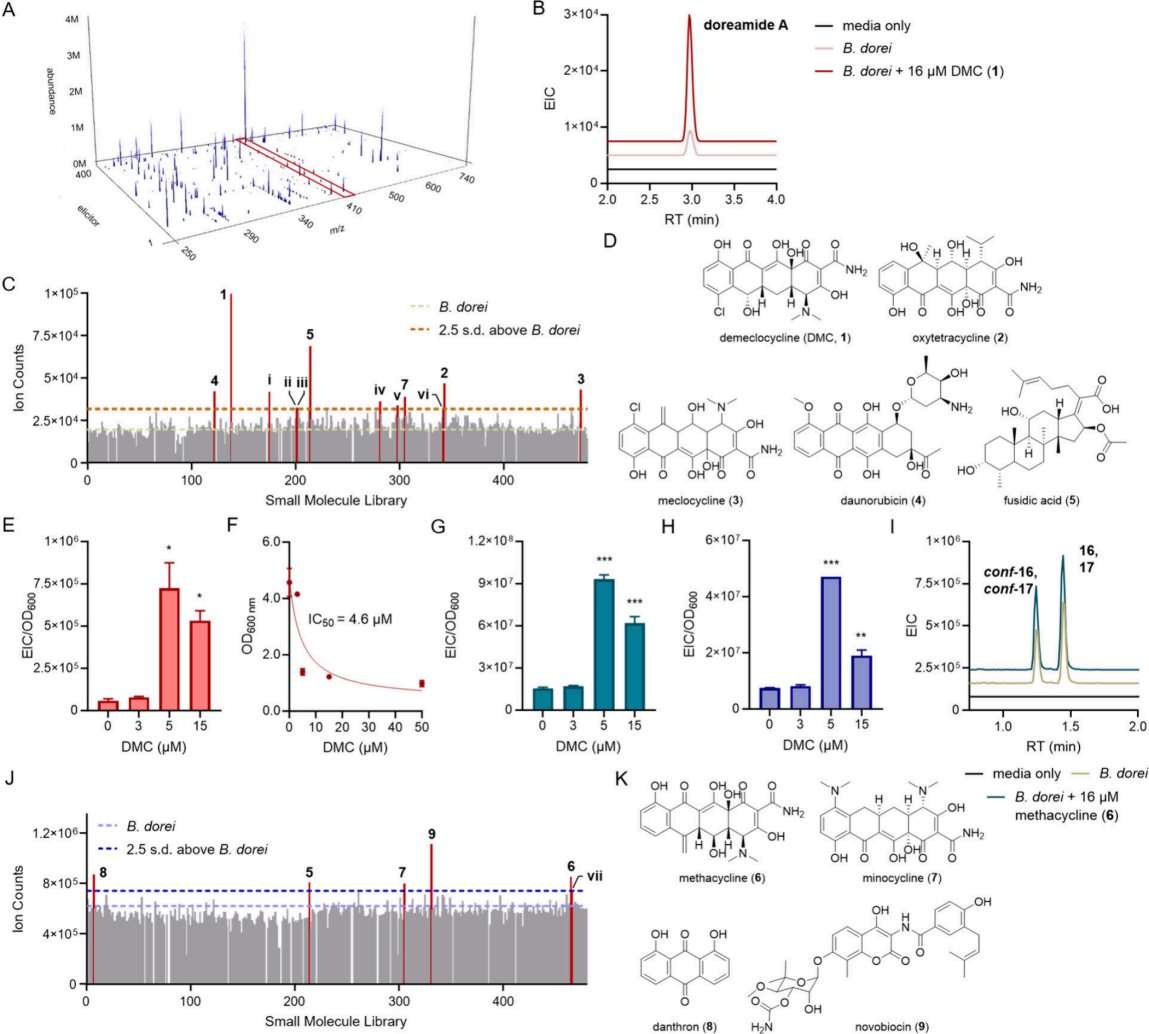
DMC serves as an inducer of secondary metabolism in *B. dorei*. (A) 3D plot of the secondary metabolome of *B. dorei* in response to 400 FDA-approved drugs. Metabolites are characterized by *m*/*z* and abundance as a function of the drug library. The *m*/*z* 417.2931 signals are highlighted with the red box. (B) Extracted ion chromatogram of *m*/*z* 417.2931 (doreamide A) in the presence or absence of 16 μM DMC. (C) Extracted ion counts of doreamide A as a function of elicitors, which are numbered. (D) Chemical structures of the top five elicitors (**1–5**) of doreamide A. (E) Dose-dependent induction of doreamide A by DMC. (F) IC_50_ analysis of DMC against *B. dorei*. (G–H) Dose-dependent induction of two isomeric metabolites with *m*/*z* 352.16 by DMC. *, **, and *** denote differences between DMC-untreated and treated conditions at *p* < 0.05, *p* < 0.01, and *p* < 0.001, respectively. (I) Extracted ion chromatogram of *m*/*z* 352.16 in the presence or absence of 16 μM methacycline (**6**). Compounds **16** and **17**, and *conf* **-16** and *conf* **-17** coelute. (J) Extracted ion counts of *m*/*z* 352.16 as a function of elicitors, which are numbered. (K) Chemical structures of the top five elicitors of *m*/*z* 352.16, including **6**–**9**. See Table S2 and Figure S2 for elicitors labeled with Roman numerals in panels C and J.

Among the metabolites detected, we initially focused on one with an *m*/*z* of 417.2931, which we have named doreamide A, as it displayed up to 5-fold elicitation relative to vehicle-control and did not return any matches in natural product databases, suggesting it is novel (Figure 1B). A 2D slice from the 3D map revealed tetracycline antibiotics, that is, demeclocycline (DMC, **1**), oxytetracycline (**2**), and meclocycline (**3**), as the best elicitors (Figures 1C, 1D, S2; Tables S2, S3). Daunorubicin (**4**) and fusidic acid (**5**) are non-tetracycline antibiotics that also showed significant induction of this compound. Tetracycline antibiotics are known to influence the overall biodiversity and composition of gut microbiota, yet little is known about metabolic perturbations that they cause.^39,40^ Considering this class of antibiotics is among the most commonly prescribed over the past six decades,^41^ we focused further efforts on the cryptic metabolites that they induce from *B. dorei*.

Analytical-scale cultures (10 mL) validated the dose-dependent stimulatory effects of DMC in a second growth format. Doreamide A was sparingly produced in the absence of DMC but was ∼12-fold induced in its presence (at 5 μM, Figure 1E). Interestingly, DMC showed a half-maximal inhibitory concentration (IC_50_) of 4.6 μM (Figure 1F), indicating that optimal production occurred at growth-inhibitory titers. This observation is consistent with previous findings in our laboratory where inhibitory molecules often stimulate secondary metabolism at low doses.^32,33^ Given that oral administration of tetracyclines yields serum concentrations of 2–5 μg/mL (4.5–11.3 μM) and its absorption is incomplete (ranging from 25–60%),^42,43^ subinhibitory levels persist in the intestinal lumen, and the 5 μM concentration falls within this physiologically relevant range. The stimulatory effects of DMC were pleiotropic, leading to the induction of a series of metabolites with 14 Da differences (403.2766, 431.3090, 431.3092, 445.3259, and 459.3392), suggesting a family of compounds with varying numbers of CH_2_ units, as well as a separate group of isomeric compounds with *m*/*z* of 352.1611 and 352.1618 but disparate retention times, which were up to 6-fold elicited relative to untreated cultures (Figures S3, 1G, 1H). A 2D slice from the 3D map for these compounds revealed the tetracyclines methacycline (**6**) and minocycline (**7**) as elicitors, as well as the related anthraquinone danthron (**8**), the aminocoumarin antibiotic novobiocin (**9**), and as with doreamide A, fusidic acid (**5**, Figures 1I–K, S4). While several elicitors identified in the initial screen display antimicrobial activities, they have distinct mechanism of actions. For example, tetracyclines target bacterial protein synthesis and daunorubicin intercalates into DNA and inhibits topoisomerase II.^43,44^ Elicitation in *B. dorei* may involve general antibiotic stress responses and/or the SOS response.^32–34^ Overall, DMC led to the activation of several natural product families, demonstrating a relatively broad effect on the secondary metabolism of *B. dorei*. We sought to determine the structures of these induced metabolites and examine their bioactivities further.

### Structure Elucidation of Doreamides

The target compounds were successfully isolated from large-scale production cultures (9 L) of *B. dorei* prepared in the presence of 4 μM of the elicitor DMC (Figure 2). Doreamide A (*m*/*z* 417.2931, **10**) was assigned a molecular formula of C_21_H_40_N_2_O_6_ based on HR-MS data (Table S4). 1D/2D NMR spectra revealed the presence of two NH amide protons, three α-protons, one oxygenated methine unit, two methyl groups, and methylene carbons (Figure S5; Table S5). Interpretation of HMBC and COSY data established a serine-glycine dipeptide linked to a β-hydroxy fatty acyl unit, and this connectivity was consistent with HR-MS/MS data (Figure S6A). The acyl group was determined to consist of a 3-OH *iso*-branched C16:0 unit (Figure 2A). Marfey’s analysis showed the Ser to be *S*-configured at the α-carbon, and the stereogenic center at C-8 was determined to be *R*-configured using electronic circular dichroism (ECD) calculations and experimental CD data (Figures S7, S8; Tables S6, S7).^45^ These analyses completed the structure of doreamide A, a new secondary metabolite and the first to be identified from *B. dorei*.

**Figure 2:**
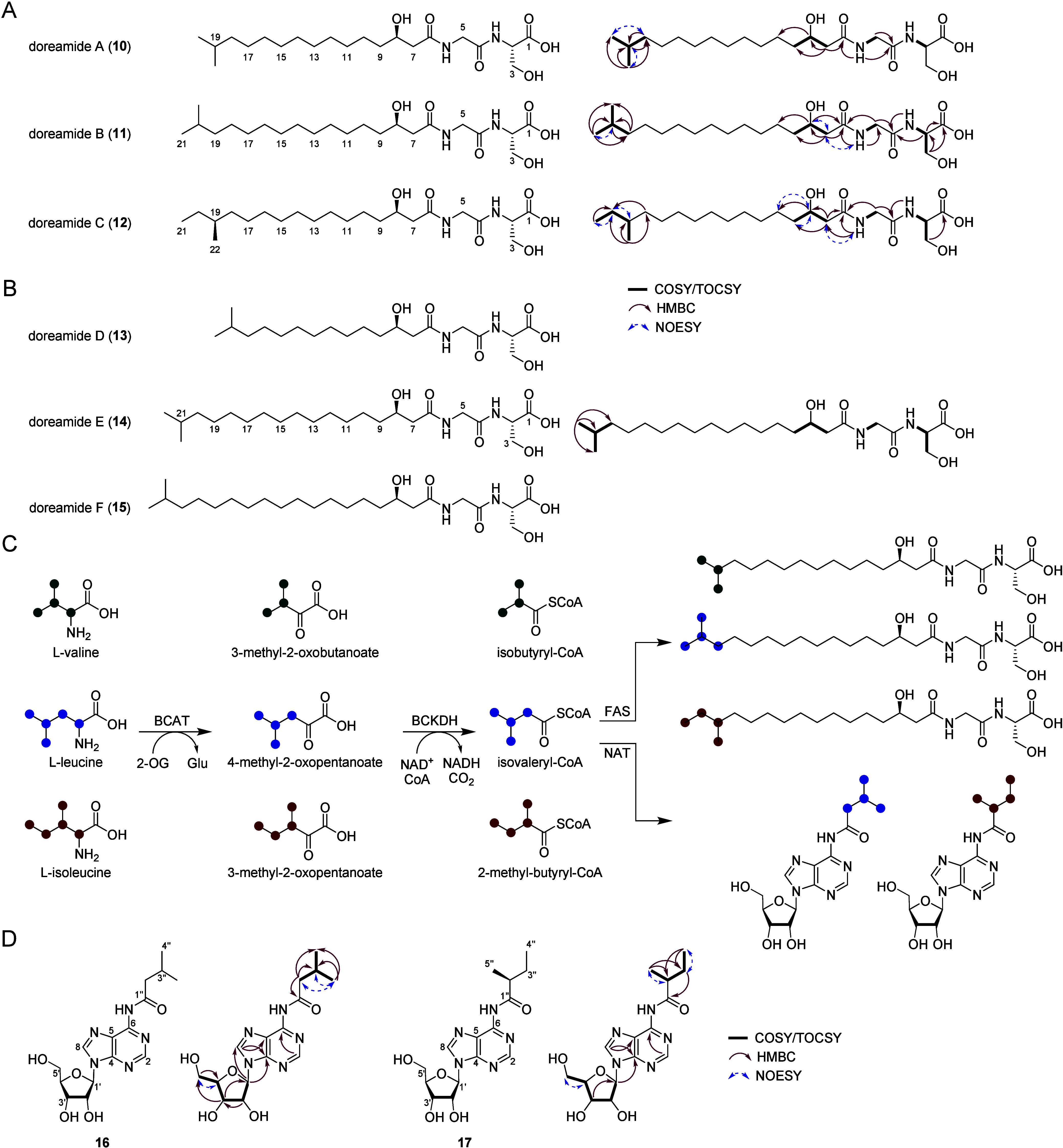
Characterization of DMC-induced metabolites. (A) Chemical structures for doreamides A–C. Key NMR correlations used to solve the structures of **10**–**12** are shown (Tables S5, S8). (B) Structures of three additional derivatives (doreamides D–F, **13–15**). The structure of variant E was elucidated by 1D/2D NMR, while those of variants D and F were deduced based on HR-MS and HR-MS/MS analysis. (C) Catabolic pathway of BCAAs and their incorporation into dipeptide lipids and adenosine. (D) Chemical structures for adenosine variants. Key NMR correlations used to solve the structures of **16** and **17** are shown (Tables S10, S11).

In addition to **10**, we detected five related metabolites, which appeared to be variants of doreamide A as their HR-MS/MS data revealed fragment ions of *m*/*z* 163.0726, 145.0618, and 106.0506, consistent with the presence of the Ser-Gly dipeptide, the dehydrated dipeptide fragment, as well as the Ser fragment, respectively (Figures 2A, 2B, S6). Of these, we isolated the two most abundant compounds, **11** (doreamide B) and **12** (doreamide C), for further NMR spectral analysis (Figures S9, S10; Table S8). Both contained the Ser-Gly dipeptide, with **11** carrying a 3-OH *iso*-branched C17:0 chain and **12** a 3-OH *anteiso*-branched C17:0 group (Figure 2A). Absolute configurations at the Ser α-carbon and C-8 were found to be identical to doreamide A (Figures S7, S8; Tables S6, S7).^45^ Compound **11** has previously been characterized from *Porphyromonas gingivalis*, although the absolute configuration of its two stereogenic centers were not reported.^46^ Doreamide C is a novel metabolite containing the unusual *anteiso* branch. Finally, the structures of the remaining variants, doreamides D–F (**13**–**15**), were either similarly elucidated by 1D/2D NMR (**14**) or deduced based on HR-MS and HR-MS/MS analysis (**13** and **15**), revealing three additional novel metabolites (Figures 2B, S6, S11; Table S9). Together, we identified a total six dipeptide lipids with fatty acyl chains carrying 15–19 carbons and different branching patterns.

We were intrigued by the different branching patterns in doreamides and explored their origins further. We suspected that branched-chain amino acids (BCAAs; leucine, isoleucine, and valine) may be involved, as they can be incorporated into lipids upon deamination to generate branched chain keto acids.^47,48^ These can be converted to the corresponding acyl-CoA species by branched-chain keto-acid dehydrogenase (BCKDH), and then enter various metabolic pathways including fatty acid synthesis. We cultured *B. dorei* with isotopically labeled BCAAs and observed distinct labeling patterns by HR-MS and HR-MS/MS analysis (Figure S12). Deuterons from uniformly deuterated l-Val (l-Val-*d*_8_) were incorporated into **10**, giving a distinct M+7 shift, suggesting isobutyryl-CoA was an intermediate for its biosynthesis (Figure 2C). The α-proton is lost in the conversion from the amino acid to the α-ketoacid. Similarly, M+9 peaks were observed for compounds **11** and **12**, when B. *dorei* cultures were supplemented with l-Leu-*d*_10_ and l-Ile-*d*_10_, respectively, pointing at isovaleryl-CoA and 2-methylbutyryl-CoA as intermediates (Figure 2C). Moreover, incorporation of l-Ile-*d*_10_ into the lipid chain of **12** suggests its C-19 is *S*-configured, thus completing the structure of variant C. These results provide insights into the biosynthesis of doreamides, which allowed us to identify the corresponding biosynthetic operon (see below).

### Structure Elucidation of *N*-Acyladenosine Derivatives

We next focused on the two isomeric metabolites with *m*/*z* 352.16 (compounds **16** and **17**) with a molecular formula C_15_H_21_N_5_O_5_ based on HR-MS (Figure 2D; Table S4). Analysis of **16** by HR-MS/MS and 1D/2D NMR established an adenosine substructure and an *N*-acyl side chain (Figures S13, S14; Table S10). COSY correlations spanning H-1′ through H-5′ showed the sequential arrangement of protons along the ribose sugar, and HMBC correlations from H-1′ to C-4 and C-8 confirmed the linkage between the adenine and ribose moieties. The presence of 3-methylbutanamide at *N*-6 was deduced based on HMBC correlations from H-2″ to C-1′′ and COSY crosspeaks from H-2′′ to H-3′′ and H-4′′. The ^1^H NMR spectrum of **17** was analogous to that of **16** except for signals corresponding to the acyl group (Figure S15; Table S11). Detailed inspection of 1D/2D NMR data of **17** revealed an *anteiso*-methyl unit, indicating the presence of 2-methylbutanamide at *N*-6. To the best of our knowledge, compounds **16** and **17** represent the first structurally characterized, natural *N*-acyladenosines from bacteria. The closest previously determined structure is phorioadenine A, a 6-*N*-acyladenine isolated from a southern Australian marine sponge *Phoriospongia* sp. Interestingly, trace amounts of the suspected adenosine analogue, corresponding to **17**, were detected as well in this study though the yields were too low for isolation.^49^

In addition to **16** and **17**, we identified conformers of each as well (*conf-* **16** and *conf-* **17**, Figure 1I). Isolation and reinjection of each conformer resulted in separation into two peaks with ratios ranging from 0.5:1 to 1:1, suggesting rapid equilibration under these conditions; we also noted the presence of minor conformers in ^1^H NMR spectra of **16** and **17** (Figures S16, S17). To confirm the conformational characteristics of these two compounds, we conducted variable-temperature (VT) ^1^H NMR experiments^50,51^ and observed that, as the temperature increased, their major and minor peaks gradually converged and the ratio of the major to minor conformers decreased (Figures S18–S21; Tables S12, S13). The methyl doublet H-4″ in compound **16**, for example, was observed at δ_H_ 0.95 ppm (major) and 0.80 ppm (minor) at 298 K, giving the chemical shift difference (Δδ_H_) of 78.87 Hz. At 378 K, Δδ_H_ decreased to 66.82 Hz, and the integration-based ratio of major to minor conformer decreased from 11.12 at 298 K to 2.17 at 378 K, supporting the conformational nature of **16** (Table S12). Similar temperature-dependent behavior was observed in other peaks of compound **16**, as well as in compound **17** (Table S13). Both *conf* **-16** and *conf* **-17** showed identical HR-MS/MS fragmentation patterns to **16** and **17** (Figure S22). Moreover, *B. dorei* cultures grown in the presence of isotopically labeled BCAAs showed incorporation of deuterons from l-Leu-*d*_10_ and l-Ile-*d*_10_ into **16**/*conf-* **16** and **17**/*conf-* **17**, respectively (Figure S22). In addition to supporting the conformational relationship, these results suggest that treatment of *B. dorei* with low-dose DMC leads to incorporation of BCAAs into doreamides as well as *N*-acylated adenosines (Figure 2C).

### Immunogenic Activity of Doreamides

With purified metabolites in hand, we sought to interrogate their biological functions. Given that the doreamides and *N*-acyladenosines were induced by tetracycline antibiotics, we speculated they may influence host physiology and therefore assessed immunogenic effects using RAW 264.7 macrophages, a common model cell line for *in vitro* studies of immunomodulators.^52,53^

Doreamides A–C exhibited no cytotoxicity at concentrations ranging from 5–40 μg/mL (12–95 μM) after 24 h exposure against macrophages using MTS assays (Figure S23A–C). However, assessment of transcript levels of genes associated with cytokines, chemokines, and regulators showed significant pro-inflammatory effects upon treatment with **10**–**12** (Figure 3A), leading to an overall 2–20-fold increase in the expression of tumor necrosis factor alpha (TNFα), interleukin (IL)-1β, IL-6, and IL-10 as well as the monocyte chemoattractant protein-1 (MCP-1), which recruits monocytes to sites of inflammation. The macrophage inflammatory proteins, MIP-1 and MIP-2, as well as cyclooxygenase-2 (Cox-2) were significantly induced as well. Among the doreamides, variant C (**12**) with the *anteiso*-methyl chain was the most effective, resulting in 4- and 22-fold induction of TNFα and IL-1β, respectively. To further validate these results, the protein levels of TNFα, IL-1β, and MCP-1 were analyzed by ELISA upon exposure to **10**–**12**, revealing up to 66-, 6-, and 20-fold induction, respectively, in response to **12** (Figure 3B–D). We also examined production of the cathelicidin antimicrobial peptide (CAMP), which is secreted by various cell types including macrophages and serves as an important effector in the innate immune response,^54^ and observed strong induction. Doreamide C (**12**), for example, resulted in 58-fold induction of CAMP (Figure 3E). Doreamides, therefore, upon elicitation by low-dose tetracyclines, are in turn strong inducers of pro-inflammatory cytokines and the host-derived antimicrobial peptide CAMP.

**Figure 3:**
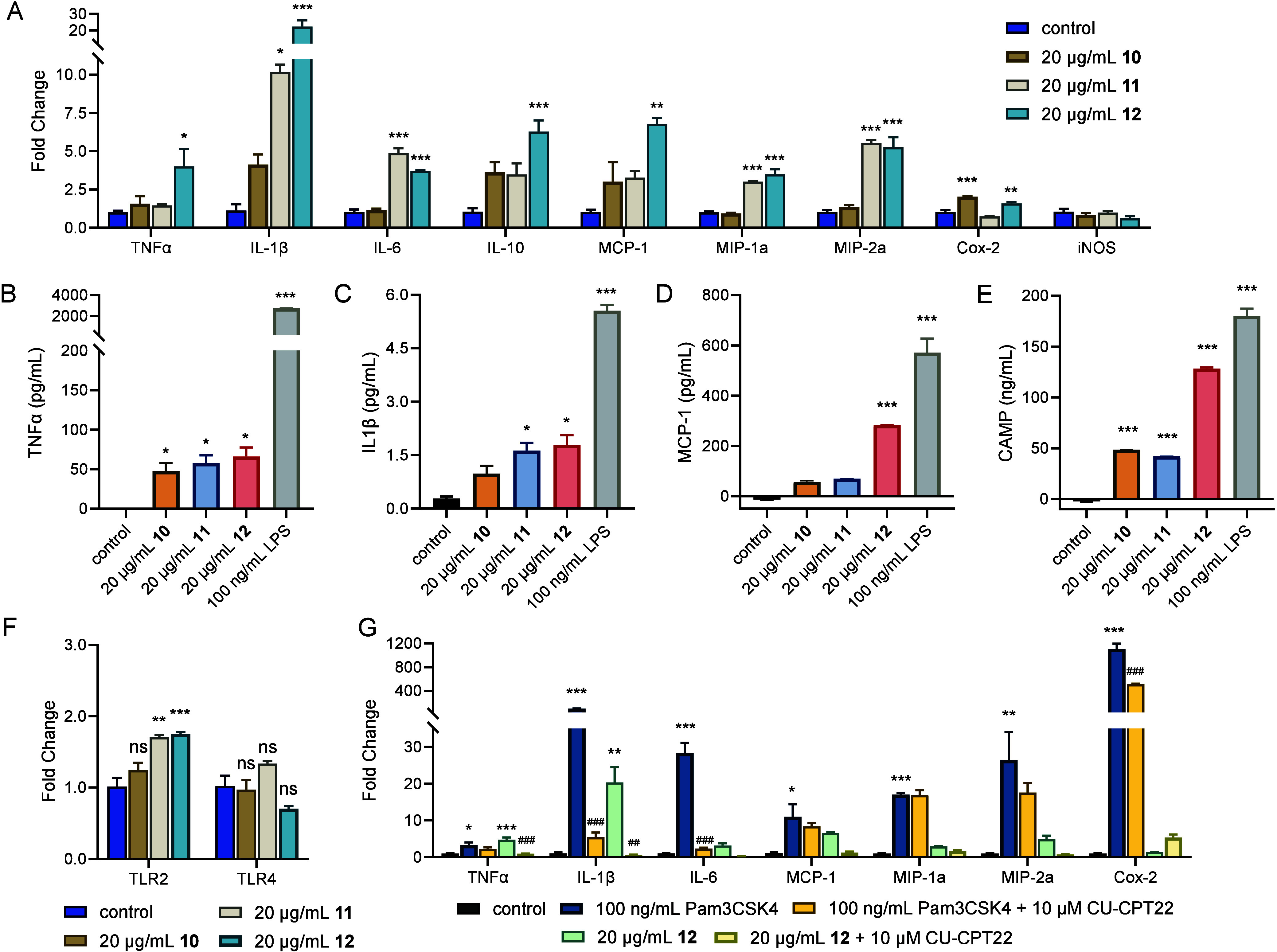
Doreamides induce a pro-inflammatory response in RAW 264.7 macrophages. (A) Effect of doreamides A**–**C (**10**–**12**) on the expression of genes associated with pro-inflammation, anti-inflammation, and immunogenetic regulators. (B–E) Change in serum levels of (B) TNFα and (C) IL-1β as well as (D) MCP-1 and (E) CAMP upon exposure to **10**–**12** or LPS for 24 h. (F) Effect of 24 h treatment of macrophages with **10**–**12** on the expression of TLR2 and TLR4. (G) Reduced expression of genes associated with pro-inflammation by treatment of CU-CPT22, a specific TLR2 antagonist, in the presence of 20 μg/mL **12**. TLR2 agonist Pam3CSK4 was used as positive control. Data represent mean ± SEM. The averages of three and two independent biological replicates are shown for PCR analysis and ELISA assays, respectively. *, **, and *** denote differences between control and the indicated condition at *p* < 0.05, *p* < 0.01, and *p* < 0.001, respectively. #, ##, and ### indicate differences between Pam3CSK4- or **12**-stimulated cells and their respective cotreatments with CU-CPT22 at *p* < 0.05, *p* < 0.01, and *p* < 0.001, respectively. Both sets of analyses were performed via a one-way ANOVA followed by a Bonferroni *post hoc* test. “ns” indicates not significant compared to unstimulated control.

Given the robust induction of CAMP by doreamides, we performed CAMP susceptibility assays against a panel of commensal or pathogenic microbes, and found CAMP to have differential antimicrobial activity against some commensal strains and opportunistic pathogens, but not against *B. dorei* (Table 1). *B. dorei* was resistant to concentrations up to 128 μg/mL. While other commensals, such as *Enterococcus faecalis* and *Streptococcus agalactiae*, also showed lower susceptibility, CAMP displayed potent antibacterial activity against *Clostridium perfringens*, *Peptostreptococcus* sp., and *Prevotella oris*, with minimal inhibitory concentrations (MICs) ranging from 4 to 8 μg/mL. These MIC values fall within the physiologically relevant range, as prior studies have reported that CAMP concentrations reach approximately 5 μg/mL at sites of infection and inflammation.^55,56^ Our results also indicate species-specific CAMP activity, as *Bacteroides fragilis* and *Bacteroides vulgatus* were more sensitive than *B. dorei*. Overall, these findings suggest that *B. dorei* contributes to host defense during tetracycline administration by producing doreamides, which enhance production of pro-inflammatory signals and an antimicrobial peptide response. Through the production of cryptic doreamides in the presence of low-dose tetracyclines, *B. dorei* could contribute to restructuring of the gut microbiome.

**Table 1:** MIC Values (μg/mL) for Cathelicidin against Select Commensal and Pathogenic Microbes

Strain	MIC
*Bacteroides dorei*	128
*Bacteroides fragilis*	8
*Bacteroides vulgatus*	4
*Clostridium perfringens*	4
*Peptostreptococcus* sp.	4
*Prevotella oris*	4
*Enterococcus faecalis*	32
*Staphylococcus aureus*	>128
*Streptococcus agalactiae*	64

Recently, lipid 654 and lipid 430 (related to **11**) were reported to function as Toll-like receptor 2 (TLR2) ligands.^46,57,58^ Though moderate, increased TLR2 expression (1.8-fold) was observed upon treatment with **12**, while levels of TLR4 remained unchanged (Figure 3F). When RAW 264.7 cells were treated with the specific TLR2 antagonist CU-CPT22 for 3 h prior to provision of **12**, transcript levels of pro-inflammatory genes were no longer induced, indicating that the effects of **12** are TLR2-dependent (Figure 3G). In particular, transcript levels of TNFα and IL-1β were significantly reduced compared to cells treated with **12** alone, with a similar trend observed for the other genes examined. Based on these results, we conclude that dipeptide lipids of *B. dorei* induce pro-inflammatory responses in a TLR2-dependent manner and, among several variants, a metabolite with a longer acyl chain and an *anteiso* branch displays stronger immunomodulatory effects than one with a shorter chain and an *iso* branch. These findings lay the foundation for future studies to examine the effects of doreamides *in vivo*.

### Immunogenic Activity of *N*-Acyladenosines

A similar set of experiments were performed for 6-*N*-acyladenosine derivatives leading to observation of pro-inflammatory responses (Figure 4). Much like doreamides, the viability of RAW 264.7 macrophages was unaffected by treatment of **16** or **17** up to 20 μg/mL for 24 h (Figure S23D,E). Exposure to **17** led to increased expression of pro-inflammatory cytokines, as confirmed by RT-qPCR, with MCP-1 exhibiting the most substantial induction (28-fold, Figure 4A). The effects on other modulators were in the 1–4-fold range.

**Figure 4:**
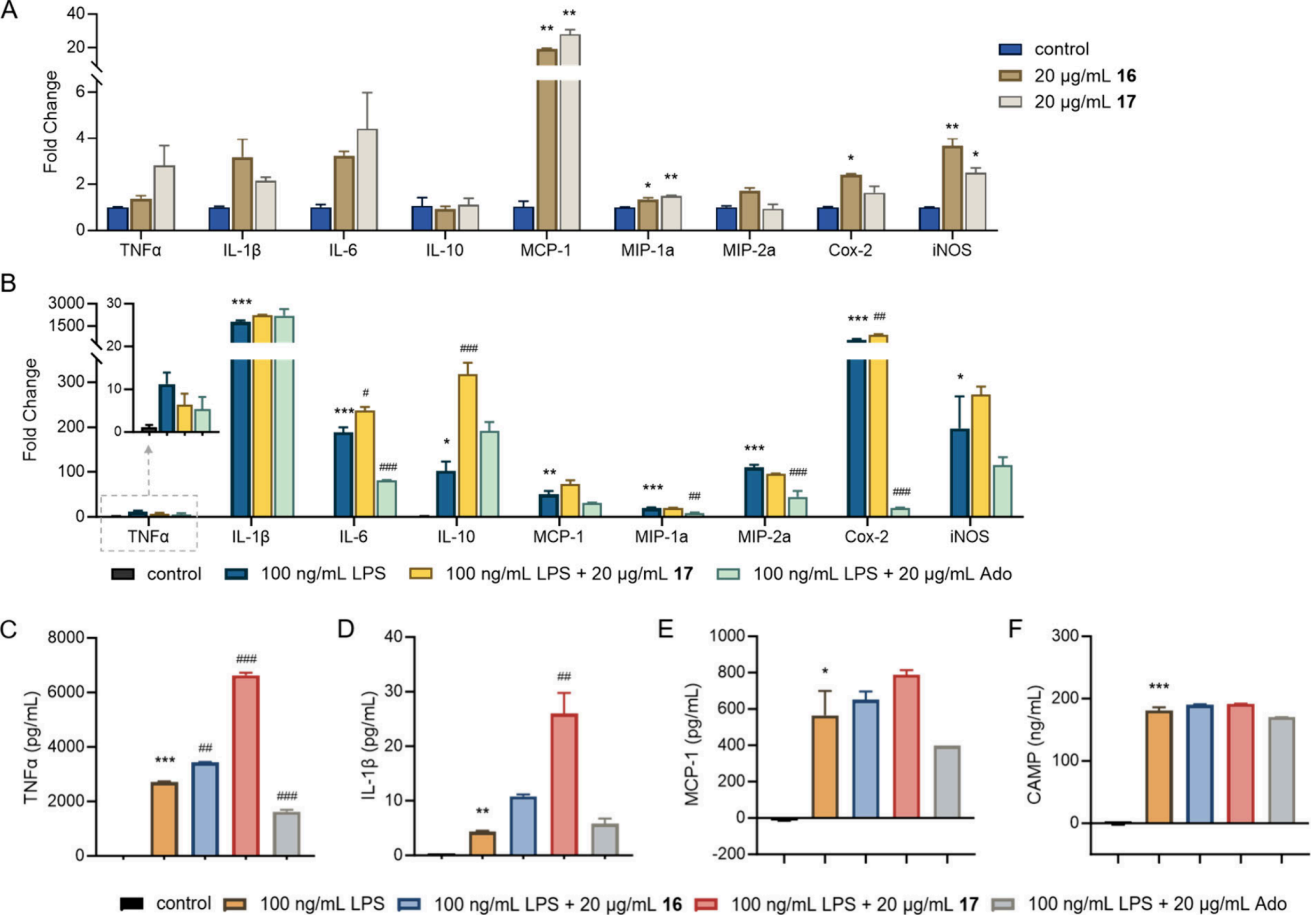
*N*-Acyladenosines induce a pro-inflammatory response in RAW 264.7 macrophages. (A) Effects of *N*-acyladenosines **16** and **17** on the expression of genes associated with pro-inflammation, anti-inflammation, and immunogenetic regulators. (B) Effect of **17** or adenosine (Ado) on cytokine expression in LPS-activated RAW 264.7 macrophages. (C–F) Increase in serum levels of (C) TNFα and (D) IL-1β cytokines as well as (E) MCP-1 and (F) CAMP upon exposure to **16**, **17**, or Ado in LPS-activated RAW 264.7 macrophages. Data represent means ± SEM (*n* = 2–3). *, **, and *** denote the difference between control and the indicated point at *p* < 0.05, *p* < 0.01, and *p* < 0.001, respectively. #, ##, and ### denote the difference between LPS-stimulated cells and the indicated condition at *p* < 0.05, *p* < 0.01, and *p* < 0.001, respectively. Both sets of analyses were performed via a one-way ANOVA followed by a Bonferroni *post hoc* test.

Because adenosine (Ado), the submoiety of **16** and **17**, has been known for its anti-inflammatory effects,^59,60^ we interrogated immunomodulatory properties of the acylated variants in the presence of lipopolysaccharides (LPS). When we treated RAW 264.7 macrophages with 20 μg/mL adenosine for 3 h prior to incubation with 100 ng/mL LPS for an additional 24 h, we observed alleviated pro-inflammatory responses induced by LPS, indicated by the significantly reduced expressions of IL-6, MIP-1a, MIP-2a, and Cox-2. These results stand in sharp contrast to the pretreatment with 20 μg/mL **17**, which retained or further enhanced expression levels of cytokines, including IL-6, IL-10, and Cox-2 (Figure 4B). Subsequent ELISA assays consistently demonstrated that treatment of **16** or **17** resulted in further increased serum levels of TNFα, IL-1β, and MCP-1 initiated by LPS, while adenosine mitigated their levels (Figure 4C–E). We suspect the modification at the 6-*N*-position causes these compounds to display pro-inflammatory properties through mechanisms distinct from adenosine. Thus, the immunomodulatory effects of adenosine can be altered by simple acylation at the 6-*N* moiety. Thin-layer chromatography (TLC) analysis and the Limulus amebocyte lysate (LAL) assays confirmed that our isolated compounds are free of detectable LPS (Figures S24, S25), indicating that the observed biological activity is attributable to **16** and **17**. We also examined the protein levels of CAMP initiated by LPS but in contrast to results with doreamides, no significant changes were observed upon addition of **16** or **17** (Figure 4F). Overall, our findings show that both doreamides and *N*-acyladenosines induce pro-inflammatory responses in macrophages, with doreamides additionally promoting CAMP production. Future studies will be necessary to determine whether these metabolites display similar effects in other cell types, such as intestinal epithelial cells and Paneth cells.

### Doreamides Are Broadly Encoded in Bacteroidota

While *B. dorei* is a prevalent and primarily probiotic species in the human gut,^35,61^ the roles of many other *Bacteroides* spp. remain to be determined. To explore whether the pro-inflammatory doreamides are broadly encoded in the genus, we used the biosynthetic clues above as well as those from prior studies on acylated amino acids to find candidate genes involved in doreamide production. Brady and colleagues previously surveyed commensal eDNA clones and identified an *N*-acetyltransferase involved in the synthesis of *N*-3-hydroxypalmitoyl-glycine, a glycine lipid (GL) termed commendamide.^62^ More recently, Lynch et al. found that deletion of a homologous *N*-acetyltransferase (*glsB*) in *B. thetaiotaomicron* abolished biosynthesis of GLs, including lipid 654 and its precursor lipid 430 (related to **11**, Figure 5).^63^ In *B. thetaiotaomicron*, *glsB* is adjacent to an additional acyltransferase (*glsA*), which is believed to catalyze the 3-OH acylation observed in lipid 654. Therefore, we suspected a BGC akin to *gls* is responsible for production of **10**–**15** in *B. dorei*. Indeed, investigation of the *B. dorei* genome showed that it harbors a *glsAB* operon (Figure 5A), which was upregulated in response to **1**, as determined by RT-qPCR analysis, while transcript levels of the branched-chain amino acid transaminase (*bcat*) remained unchanged (Figure S26).

**Figure 5:**
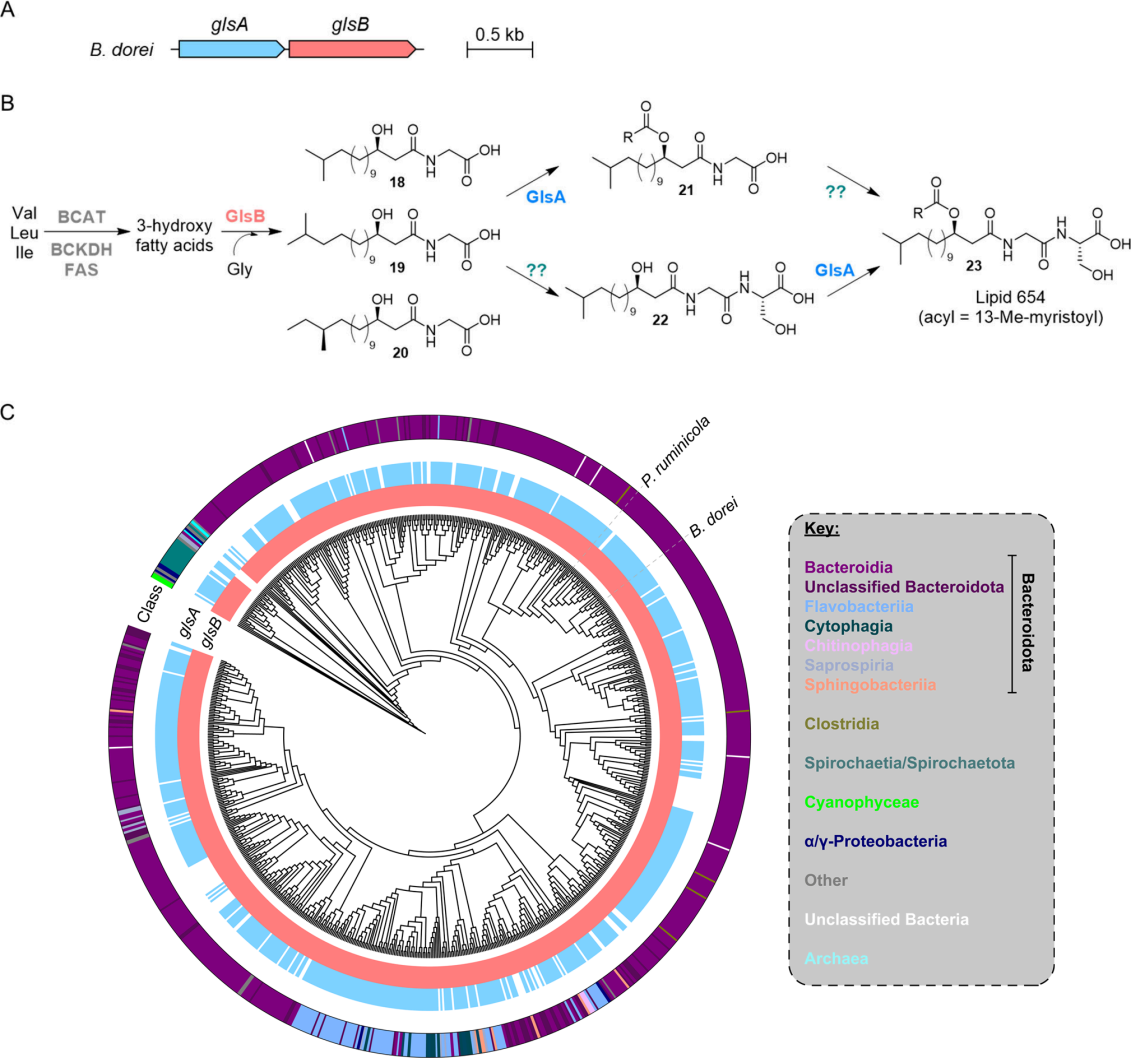
Prevalence of the *glsAB* BGC in bacterial genomes. (A) The two-gene cluster coding for doreamide in the genome of *B. dorei*. (B) Proposed biosynthetic pathway for **10**–**15** and their acylated and dipeptide derivatives. The gene involved in the ligation of Ser remains to be identified (‘??’). Note, acylated derivatives, like lipid 654, are not observed in *B. dorei*. Moreover, dipeptide compounds **22** and **23** are not observed when heterologously expressing *B. dorei glsAB* in *E. coli*. (C) Phylogenetic tree of GlsB proteins and distribution of 841 *glsA* and/or *glsB* sequences; the two-gene operon is almost exclusively encoded in Bacteroidota.

Repeated efforts to generate *gls* deletion or insertional inactivation mutants failed. However, heterologous expression of *B. dorei glsA* and *glsB* individually or together in *E*. *coli* mirrored previous findings,^63^ supporting their role in doreamide biosynthesis. Specifically, expression of *glsB* alone led to the production of several GLs (e.g., **18**–**20**), whereas *glsA* alone did not yield detectable GLs or doreamides (Figures 5A, S27; Table S15). Coexpression resulted in the production of several bisacylated GLs, analogous to **21**, supporting the 3-OH acylation activity of GlsA (Figures S27–S30). Notably, heterologous expression did not produce *N*-acylated Gly-Ser dipeptide analogs (e.g., **22,** Figure 5B). GLs likely serve as precursors for the dipeptide lipids,^63^ suggesting that the Gly-Ser peptide may be formed by a protein encoded outside the *glsAB* operon. We searched for proteins containing the following PFAM domains commonly associated with peptide bond formation: PF02222, PF00668, PF00860, PF00501, PF04262, and PF07478. However, no matches were found beyond primary metabolic enzymes or proteins with already characterized functions. Interestingly, while *glsA* is encoded in *B. dorei*, we did not detect any bisacylated lipid 654-type compounds (e.g., **23**) in our studies.

Searches of the *B. dorei* GlsB protein against the NCBI nonredundant protein database, followed by hierarchical clustering of the initial hits identified 841 putative GlsB homologous groups with at most 75% identity to each other. Subsequently, co-occurrence with GlsA and phylogeny data were mapped onto a phylogenetic tree of a multiple sequence alignment of all 841 hits (Figure 5C), showing that the two-gene *glsAB* cluster is widespread in Bacteroidota and mainly limited to this phylum (Figure 5C). Indeed, a comprehensive analysis of all NCBI reference genomes in the Bacteroidales order shows the *gls* cluster is encoded in >96% of the representative strains (Table S16), including various commensal and human-associated bacteria as well as other strains isolated from disparate ecological niches. To assess whether the tetracycline-mediated induction effect is general across multiple *Bacteroides*, we screened two additional strains, *Bacteroides* sp. 1_1_30 and *Bacteroides dorei* CL02T12C06, the genomes of which encode the doreamide biosynthetic pathway. In both cases, we observed significant induction of compounds **10**, **11**, and **12**, by DMC in a dose-dependent manner (Figure S31). This induction effect was also observed for compound **14** in *B. dorei* CL02T12C06, though *strain* sp. 1_1_30 did not show detectable production of **14**. Moreover, while strain CL02T12C06 produced **16** and **17**, strain 1_1_30 did not. We identified the *gls* operon in a small number of other bacterial taxonomies, including Clostridia and Spirochaete; however, it was not broadly encoded in these genera and metabolites have not been connected to the corresponding BGCs.

The presence of the *gls* cluster across diverse members within Bacteroidota suggests a conserved functional role. Previous work has demonstrated that deletion of *glsB* in *B. thetaiotaomicron* results in a hampered ability to colonize the mouse gut as well as lowered tolerance to bile acid and oxygen *in vitro*.^63^ Likewise, the doreamides could provide a selective advantage by facilitating survival upon exposure to antibiotics or other stressors in a host context. In addition, they may be used to delay or prevent expansion of evading pathogens through activation of pro-inflammatory signals and CAMP, thus contributing to the host’s adaptive immune response.

## Conclusions

Microbes communicate with other organisms using an array of secondary metabolites, and these molecule-mediated associations can be especially complex in animal microbiomes, where hundreds of species compete for limited nutrients and simultaneously interact with each other and with host cells. The molecules that facilitate these multipartite interactions in the human microbiome largely remain to be determined. An additional complicating factor is that these interactions are subject to exogenous stressors, antibiotics, or other metabolites that the host may intake. This aspect, the modulation of microbiome metabolites by exogenous molecules, has received far less attention and is the topic of the current work. Our findings point to one possible mechanism by which exogenous molecules may perturb gut bacterial composition and regulate immune response, a process that is mediated by induced or cryptic metabolites.

Our work was conducted *in vitro* and needs to be assessed further in animal models. This limitation notwithstanding, the results show that low-dose tetracyclines, a group of antibiotics that has been prescribed heavily over the past six decades and has been consumed by humans for several thousand years,^64–66^ leads to a metabolite-mediated immunogenic response in *B. dorei*. Specifically, the antibiotics induce biosynthesis of doreamides, secondary metabolites composed of Gly-Ser dipeptides and fatty acids that incorporate branched chain amino acids. The doreamides in turn induce production of various pro-inflammatory cytokines (Figure 6). While persistent inflammation contributes to numerous pathological conditions, acute inflammation is beneficial to the host and serves as a protective mechanism in response to infections and dysbiosis. A similar response is observed with *N*-acyl adenosines, *B. dorei* metabolites that are also induced by tetracyclines and incorporate branched chain amino acids. Acylation of adenosine seems to convert this otherwise anti-inflammatory precursor to an inflammation-inducing metabolite that triggers production of key cytokines. Thus, tetracyclines act as pleiotropic inducers of secondary metabolism in *B. dorei*, triggering enhanced synthesis of immunogenic small molecules. Future studies are necessary to elucidate the regulatory pathways underlying the stimulatory effects of tetracyclines and to assess whether other drugs or natural stressors, such as bile acids or oxidative stress, serve as elicitors.

**Figure 6:**
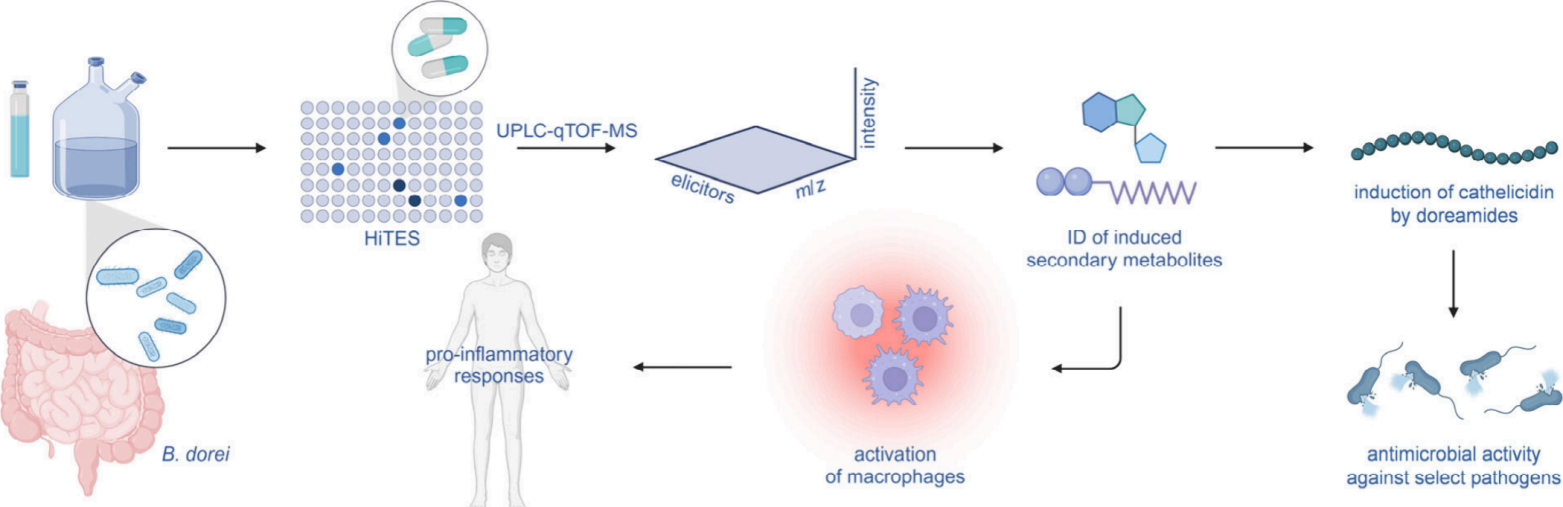
Model for the pro-inflammatory effects mediated by *B. dorei* cryptic metabolites. Low-dose tetracycline antibiotics, identified using a forward chemical genetic screen, pleiotropically induce production of doreamides and 6-*N*-acyladenosines from *B. dorei*, all of which in turn induce synthesis of pro-inflammatory cytokines. The doreamides additionally trigger CAMP production, thereby augmenting the innate immune response and possibly reshaping the local microbiome.

In addition to pro-inflammatory signals, the doreamides induce production of the host-derived antimicrobial peptide cathelicidin, which, like other AMPs, exhibits differential antibiotic activity. *B. dorei* is resistant to high concentrations of CAMP, but several other bacteria tested, including opportunistic pathogens, are highly susceptible. It is conceivable that this sequence, initiated by tetracyclines, mediated by doreamides, and culminating in the production of CAMP and inflammatory cytokines, leads to local reshaping of bacterial composition (Figure 6). It is by now established that antibiotics can prune or restructure local microbiomes through their effects on bacteria. Our results suggest that there is another mechanism by which antibiotics can alter bacterial composition, one that occurs through cryptic metabolites that are elicited by low-dose antibiotics in gut bacteria. These findings contribute to the growing body of work on drug-microbiome interactions^67,68^ and, by exploring the production and function of induced metabolites, extend beyond the traditional focus on microbial drug metabolism.

Beyond this suggested interplay, we uncover new cryptic metabolites from the gut microbiome, which motivates further inquiries into this largely unknown chemical space. Interestingly, the doreamides and *N*-acyladenosines are produced by genes or gene clusters that are not captured by typical genome mining tools, thus highlighting the strength of chemistry-first approaches in unearthing the metabolomic potential of gut bacteria. A major benefit of this approach is that it remains unbiased by genome mining and is not restricted to molecules with limited or straightforward activities. This type of chemistry-first discovery approach is poised to unearth more natural products, thousands of which are predicted from the human microbiome, thus enabling insights into the roles that small molecules play in human health and disease.

## Supplementary Material



## Notes

Detailed descriptions of materials and methods, including UPLC-qTOF-MS-guided HiTES, analysis of the resulting data, isolation of doreamides and *N*-acyladenosines, structure elucidation of these compounds, cytotoxicity and cytokine induction assays, heterologous expression of *glsAB*, and bioinformatic search for the prevalence of *glsAB* (PDF)
