# Supplementary material for: Tetracycline Antibiotics Induce Biosynthesis of Pro-Inflammatory Metabolites in the Immunobiotic Bacteroides dorei
Source: ACS Cent Sci. 2025 Dec 3;11(12):2421–32. doi: 10.1021/acscentsci.5c00969 (PMC12746155; doi:10.1021/acscentsci.5c00969)
Supplement: Supplementary file 1 [file oc5c00969_si_001.pdf]

Supporting Information for

Tetracycline Antibiotics Induce Biosynthesis of Pro-inflammatory Metabolites in the Immunobiotic *Bacteroides dorei*

Esther J. Han,[1] Jack G. Ganley,[1] Caitlin B. Winner,[1] Joon Soo An,[1] and Mohammad R. Seyedsayamdost[1,2,*]

[1]Department of Chemistry, Princeton University, Princeton, NJ 08544, United States

[2]Department of Molecular Biology, Princeton University, Princeton, NJ 08544, United States

[*]Email: mrseyed@princeton.edu

Materials and Methods

General Procedure

Bacterial strain and culture media. *Bacteroides dorei* CL02T00C15, *Bacteroides fragilis* HM-20, and *Bacteroides vulgatus* CL09T03C04 were kindly provided by the Donia lab at Princeton University. *Clostridium perfringens* WAL-14572, *Peptostreptococcus* sp. MV1, *Prevotella oris* F0302, *Bacteroides* sp. 1_1_30, and *Bacteroides dorei* CL02T12C06 were purchased from BEI Resources, and *Enterococcus faecalis* OG1RF, *Staphylococcus aureus* F-182, and *Streptococcus agalactiae* NCTC 8181 from ATCC. *Escherichia coli* BL21(DE3) was obtained from New England Biolabs (NEB). All materials were purchased from Sigma-Aldrich or Fisher Scientific unless specified otherwise. Custom gene and plasmid synthesis was completed by Twist Biosciences. All culture media were purchased from Becton-Dickinson unless noted otherwise. Gifu Anaerobic Broth (GAM, HiMedia Laboratories) was used as seed media. Chemically defined media (CDM) was used as fermentation medium; it consisted of 0.5% (w/v) glucose, 5% (v/v) mineral 3B solution, 0.1% (w/v) L-cysteine, 1% (v/v) hemin solution, 0.002% (w/v) L-methionine, 0.0004% (w/v) $FeSO_4 \cdot 7H_2O$, 0.2% (w/v) $NaHCO_3$, and 0.0001% (w/v) resazurin. Mineral 3B solution contained per liter: 18 g KH_2PO_4, 18 g NaCl, 0.4 g $MgCl_2 \cdot 6H_2O$, 0.52 g $CaCl_2 \cdot 2H_2O$, 0.02 g $CoCl_2 \cdot 6H_2O$, 0.2 g $MnCl_2 \cdot 4H_2O$, 10 g of NH_4Cl, and 5 g of Na_2SO_4. For hemin solution, 100 mg of hemin was dissolved in 2 mL of 1 M NaOH and volume was brought up to 200 mL with distilled water. $FeSO_4$ solution contained per 100 mL: 0.278 g of $FeSO_4 \cdot 7H_2O$ and two drops of concentrated HCl.

Mammalian cell lines and culture media. RAW 264.7 cell line used in this study was kindly provided by the Kang lab at Princeton University. RAW 264.7 cells were cultured with Dulbecco's modified Eagle's medium (Gibco Corp.) containing 10% fetal bovine serum (FBS, Biotchene), 1% Penicillin-Streptomycin (Thermo Fisher), and 2 mM L-glutamine (Thermo Fisher) in a 5% CO_2 incubator at 37°C.

Routine HPLC-MS and NMR Analysis

High-resolution (HR) HPLC-qTOF-MS was performed on an Agilent 6456 accurate-mass quadrupole time-of-flight (qTOF) MS instrument equipped with an automated liquid sampler, a 1260 Infinity II Series LC system, a diode array detector, a JetStream ESI source, and the 6456 Series qTOF. An analytical Kinetex C18 column (Phenomenex, 2.6 µm, 4.6 x 100 mm) was used to resolve samples, unless otherwise stated. Routine elution was carried out at a flow rate of 0.5 mL/min with a gradient of 5–95% MeCN over 15 min in H_2O, and both mobile phases contained 0.1% (v/v) formic acid (FA). HPLC purifications were performed on an Agilent 1260 Infinity Series preparative or semi-preparative HPLC system, which consisted of a diode array detector, automated liquid sampler, and automated fraction collector. Specific columns and gradients are described in the relevant sections below. 1D/2D NMR spectra were acquired at the Princeton University Department of Chemistry Facilities on a 500 MHz NMR spectrometer (Bruker) equipped with a ^1H-optimized cryoprobe.

High-throughput Elicitor Screening with *B. dorei*

A frozen glycerol stock of *B. dorei* was used to inoculate a 10 mL seed culture of GAM in an 18 mL anaerobic culture tube (Chemglass Life Sciences). The seed culture was grown anaerobically at 37°C for 2 d in a COY type B anaerobic chamber using gas mix, consisting of 5% H_2, 85% N_2, and 10% CO_2, and an anaerobic gas infuser to maintain H_2 at approximately 3.3%. All media and materials were pre-reduced in the anaerobic chamber for at least 24 h prior to use. HiTES plates were prepared by dispensing 150 µL CDM into each well of five deep-well 96-well plates and supplementing 0.5 µL of each candidate elicitor from a commercially available FDA-approved drug library (a stock concentration of 10 mM in DMSO, Enzo Scientific) using a CyBi-Well automated liquid transfer robot (Cybio). Plates were reduced for 24 h in an anaerobic chamber. Next, cells were inoculated into 75 mL of pre-reduced CDM to give an optical density at 600 nm (OD_{600}) of 0.1. Subsequently, 150 µL of cells were added into each well containing the elicitor-supplemented 150 µL CDM to give the initial OD_{600} of 0.05 and elicitor concentration of 16.7 µM. Plates were covered with a Breathe-Easy® air-permeable sealing membrane (Sigma-Aldrich) and incubated under anaerobic conditions at 37°C for 4 d. After 4 d, 200 µL of MeOH was added to each well, and the plates were spun down (4000-g, 30 min) to pellet the cells. Supernatants were filtered into clean 96-well plates using 96-well plate vacuum filters (Fisher Scientific). Filtered plates were then covered with pierceable sealing mats (Phenomenex) and analyzed by UPLC-qTOF-MS. Supernatants in each well were resolved on an UPLC Kinetex C18 column (Phenomenex, 1.7 µm, 2.1 x 50 mm) operating at 0.5 mL/min flow rate. Elution was carried out using water and MeCN as mobile phases, both containing 0.1% FA, with a gradient of 5–100% MeCN over 4 min. Collected LC-MS runs were analyzed by Metabolomic Explorer (MetEx) application as detailed below.

Feature Extraction and Data Analysis

After HR-MS data collection, *m/z* and retention time features were extracted across all samples using Agilent Profinder v. B.06.00. An ion count of $\geq 10^4$ was set as the threshold parameters, and multi-dimensional data sets containing discrete *m/z* values and retention times above this threshold were further analyzed using MetEx [1]. The *m/z* and abundance for induced metabolites were displayed as a function of elicitor in a 3D plot, and the obtained 3D plot along with the component 2D slices were used for dereplication and induced metabolite identification.

Validation of UPLC-HiTES Elicitor Candidates

A frozen stock of *B. dorei* was inoculated into 10 mL GAM in an 18 mL anaerobic culture tube and incubated at 37°C for 2 d in the anaerobic chamber. The seed cultures were then used to inoculate ten 18 mL culture tubes each containing 10 mL of CDM at an initial OD_{600} of 0.05. Varying concentrations (0, 3, 5, 15, and 50 µM in DMSO) of demeclocycline (DMC, **1**) were supplemented in duplicates and the cultures were incubated at 37°C for 4 d. Subsequently, the cultures were extracted twice with 15 mL of ethyl acetate. The organic phases were dried *in vacuo*, and the resulting samples were

dissolved in 1 mL of MeOH for HPLC-qTOF-MS analysis as detailed in general procedure above.

Large-scale Fermentation of *B. dorei*

Large-scale fermentation was carried out following a similar procedure to that for the small-scale fermentation described above. This time, 3 x 3 L of CDM in 5 L round bottles (Fisher Scientific) were prepared with an initial OD_{600} of 0.05. The *B. dorei* cultures were supplemented with 4 µM **1** and anaerobically grown at 37°C for 4 d.

Isolation and Structure Elucidation of Compounds 10–12

After 4-d of growth, bacterial cultures were centrifuged to separate cell pellets and supernatants (4000-g, 30 min). The resulting supernatants were extracted twice with an equal volume of ethyl acetate. The organic phases were combined and dried under reduced pressure to yield 300 mg of crude extract. The sample was resuspended in MeOH and purified on a preparative HPLC Luna Phenyl-Hexyl Column (Phenomenex, 5 µm, 21.2 × 250 mm), operating at a flow rate of 10 mL/min with a gradient of 10–100% MeCN (+0.1% FA) over 32 min to afford fourteen subfractions (A1–A14). Fraction A4, which contained two *m/z* 352.16 metabolites (**16** and **17**), was resolved on a semi-preparative Kinetex C18 Column (Phenomenex, 5 µm, 10.0 × 250 mm) operating at a flow rate of 2 mL/min. Pure **17** (0.9 mg) and **16** (1.0 mg) were acquired at retention times of 36 and 38 min, respectively, using isocratic elution at 15% MeCN (+0.1% FA). From fraction A10, *m/z* 417.29 (0.7 mg, **10**) was purified at a retention time of 29 min using isocratic elution at 51% MeCN (+0.1% FA).

The cell pellets were extracted with acetone and MeOH (1:1) for 24 h at room temperature. The solvent mixture was filtered and dried under vacuum, yielding 200 mg of crude extract. The sample was resuspended in MeOH and purified on the same preparative HPLC Luna Phenyl-Hexyl Column (Phenomenex, 5 µm, 21.2 × 250 mm), operating at a flow rate of 10 mL/min with a gradient of 10–100% MeCN (+0.1% FA) over 32 min to afford fourteen subfractions (B1–B14). Fraction B11, which contained *m/z* 431.31 (compounds **11** and **12**), was resolved on a semi-preparative Kinetex C18 Column (Phenomenex, 5 µm, 10.0 × 250 mm) operating at a flow rate of 2 mL/min. Pure **12** (0.9 mg) and **11** (1.1 mg) were acquired at retention times of 29 and 31 min, respectively, using 55% MeCN isocratic solvent (+0.1% FA) over 60 min.

Large-scale fermentation and purification of compounds of 14, 16 and 17. An additional 30 L of large-scale fermentation was performed to isolate and purify compounds **14**, **16**, and **17**, following the same procedures described above. This time, 30 × 1 L of CDM was cultured in 1 L round bottles. After 3 d of growth, the cultures were extracted twice with 1.5 volumes of ethyl acetate using a separation funnel. The organic layers were combined and dried over anhydrous sodium sulfate to remove residual water. The crude extract was concentrated using a rotary evaporator, yielding approximately 1 g of extract. The extract was subjected to reversed-phase open-column chromatography (60 × 40 mm, YMC*GEL ODS-A, 12 nm, S-75 µm), and fractionated by vacuum liquid chromatography (VLC) using 400 mL of stepwise MeOH/water fractions (20%, 40%, 60%,

80%, and 100%) followed by 50% MeOH/DCM. Doreamide E (**14**) was observed in the 100% MeOH/water and 50% MeOH/DCM fractions. These fractions were concentrated and purified using a preparative HPLC column (Phenomenex Luna C18(2), 250 × 21.2 mm, 5 μm) with a gradient of 60–100% MeCN containing 0.1% FA over 40 min (flow rate: 10 mL/min). **14** was eluted at 18 min and further purified using a semi-preparative HPLC column (Luna C18(2), 250 × 10 mm, 5 μm) under a step-gradient system: isocratic 60% MeCN for 50 min followed by a step to 65% (+0.1% FA), at a flow rate of 2.0 mL/min. **14** was eluted at 59 min (0.4 mg). Both **16** and **17** were detected in the 60% and 80% MeOH/water fractions. These were concentrated and purified using a preparative HPLC column (Phenomenex Luna C18(2), 250 × 21.2 mm, 5 μm) under a gradient from 25% to 55% MeCN containing 0.1% FA over 30 min (flow rate: 10 mL/min). Both compounds eluted at 12 min. Further purification was carried out on a semi-preparative Kinetex C18 column (250 × 10 mm, 5 μm) using an isocratic system of 15% MeCN with 0.1% FA (flow rate: 2.0 mL/min), with **16** eluting at 33 min and **17** at 36 min. Final purification was performed on a semi-preparative Luna C18(2) column (250 × 4.6 mm, 5 μm) using the same isocratic system (15% MeCN+0.1 FA, flow rate: 1.0 mL/min). **16** was eluted at 9.5 min (2.3 mg), and **17** at 10 min (3.0 mg).

Marfey's analysis of compounds 10–12. Approximately 0.1 mg of pure **10–12** was hydrolyzed in 0.5 mL of 6 N HCl in a 110°C oil bath for 6 h. Hydrochloric acid was dried *in vacuo* and 100 μL of 1 N $NaHCO_3$ was added to the resulting hydrolysate. To an Eppendorf tube, 50 μL of the hydrolysate was added and supplemented with 50 μL of L-FDLA or D-FDLA (1% w/v in acetone) and the mixture was heated at 80°C for 10 min. The reaction was quenched with 20 μL of 1 N HCl. Derivatization of L-serine standard with L-FDLA and D-FDLA was performed following a similar procedure. Hydrolysate of **10–12** as well as standards were analyzed by HPLC-qTOF-MS. Separation of FDLA-derivatized amino acids was achieved on a Kinetex C18 column (Phenomenex, 2.6 μm, 4.6 x 100 mm) operating at a flow rate of 0.5 mL/min. Elution was carried out isocratically with 10% MeCN in H_2O for 5 min followed by an initial gradient of 10–45% MeCN over 35 min and a second gradient of 45–95% MeCN over 10 min. The mobile phases contained 0.1% FA. The retention times for authentic standards were as follows: L-Ser-L-FDLA, 39.02 min and L-Ser-D-FDLA, 39.83 min. The retention times for dipeptide lipid derivatives were as follows: **10**-L-FDLA, 39.18 min; **10**-D-FDLA, 39.91 min; **11**-L-FDLA, 39.02 min; **11**-D-FDLA, 39.89 min; **12**-L-FDLA, 39.08 min; and **12**-D-FDLA, 39.81 min.

Computational analysis for ECD simulations for dipeptide lipid variants [2]. All conformers reported in this study were computed using the MacroModel module (version 2019-3, Schrödinger LLC) with 'mixed torsional/low mode sampling' implemented with the MMFF94 force field. Searches were performed in the gas phase with a 5 kJ/mol energy window limit and 10,000 maximum number of steps to consider all possible conformers. The Polak-Ribiere conjugate gradient (PRCG) protocol was conducted with 10,000 maximum iterations and a 0.001 kJ (mol Å)$^{-1}$ convergence threshold on the rms gradient to minimize conformers. Conformers acquired within 10 kJ/mol in the MMFF force field were selected for the geometry optimization by Tmolex 4.3.1 using DFT settings of B3-LYP/6-31+G(d,p) level. ECD calculations for conformers **11a** (2*S*, 8*R*) and **11b** (2*S*, 8*S*)

were carried out at the identical theory levels and basis sets. Calculated ECD spectra were simulated by overlying each transition, where σ is the width of the band at the height of 1/e (see equation below). The ΔE_i and R_i are the excitation energies and rotatory strengths for transition *i*, respectively, and the value of σ was 0.20 eV. The excitation energies and rotational strengths for ECD spectra were calculated based on the Boltzmann populations of conformers and ECD visualization performed using the GraphPad Prism 9 software (GraphPad Software, San Diego, CA, USA).

$$\Delta\epsilon(E) = \frac{1}{2.297 \times 10^{-39}} \frac{1}{\sqrt{2\pi\sigma}} \sum_{A}^{i} \Delta E_i R_i e^{[-(E-\Delta E_i)^2/(2\sigma)^2]}$$

Variable temperature (VT) NMR analysis of compounds 16 and 17. ^{1}H NMR spectra were acquired at 298, 308, 318, 328, 338, 348, 358, 368, and 378 K using a 500 MHz NMR spectrometer. As the temperature increased, the major and minor peaks gradually converged, and the ratio of the major to minor conformer decreased (Figures S18–S21, Tables S12 and S13). For example, in compound **16**, the methyl doublet H_3-4″ appeared at δ_H 0.95 ppm (major) and 0.80 ppm (minor) at 298 K. The chemical shift difference ($\Delta\delta_H$) between the two signals was 78.87 Hz at 298 K, which decreased to 66.82 Hz at 378 K. The integration-based ratio of major to minor conformer decreased from 11.12 at 298 K to 2.17 at 378 K (Table S12). Other peaks exhibited similar temperature-dependent behavior, supporting the presence of conformational mixture. Likewise, compound **17** also exists as a conformational mixture.

Identification of Isotopically Labeled Doreamides A–C and 6-*N*-acyladenosines. A frozen stock of *B. dorei* was inoculated into 10 mL GAM in an 18 mL anaerobic culture tube and incubated at 37°C for 2 d in the anaerobic chamber. The seed cultures were used to inoculate eight 18 mL culture tubes each containing 10 mL of CDM at an initial OD_{600} of 0.05. L-valine-d_8, L-leucine-d_{10}, and L-isoleucine-d_{10} (Cambridge Isotopes Inc.) were supplemented to a final concentration of 0.5 mM in duplicates, and these cultures along with controls were incubated at 37°C. After 4 d, the cultures were extracted twice with 15 mL of ethyl acetate. The organic phases were dried *in vacuo* and the resulting samples were dissolved in 1 mL MeOH for HR-MS and HR-MS/MS analysis as described in general procedure above.

Bioactivity Assays and Immune Profiling for Doreamides A–C and 6-*N*-acyladenosines

MTS assay. Viability of RAW 264.7 cells was evaluated using the CellTiter 96® AQueous One Solution Cell Proliferation Assay (Promega), which is based on the 3-(4,5-dimethylthiazol-2-yl)-5-(3-carboxymethoxyphenyl)-2-(4-sulfophenyl)-2H-tetrazolium (MTS) reduction assay. Cells were seeded at a density of 1 x 10^4 cells per well in 96-well plates and incubated overnight in a 5% CO_2 incubator at 37°C to ensure cell attachment. Cells were then treated with doreamides A–C (**10**–**12**) or 6-*N*-acyladenosines (**16** and **17**) at concentrations ranging from 5 to 40 µg/mL for 24 h. After each treatment, media was discarded and fresh DMEM (100 µL) and MTS solution (20 µL) were added to each well.

After 1 h incubation at 37°C, cell viability was evaluated according to the manufacturer's instructions by measuring the absorbance of the solution at 490 nm using a Synergy H1 microplate reader (Biotek).

Reverse transcription quantitative polymerase chain reaction (RT-qPCR). RAW 264.7 cells were seeded at a density of 1 x 10^5 cells per well in 6-well plates and incubated overnight in a 5% CO_2 incubator at 37°C to ensure cell attachment. Subsequently, cells were treated with 20 µg/mL doreamides A–C (**10**–**12**) or 6-*N*-acyladenosines (**16** and **17**) for 24 h. Cells with DMSO treatment served as the negative control and cells with 100 ng/mL LPS from *Escherichia coli* O26:B6 (Sigma) served as the positive control. For dipeptide lipid variants, additional co-treatment experiments were carried out using the specific TLR2 antagonist CU-CPT22 (Sigma). To this end, 10 µM CU-CPT22 was introduced to macrophages for 3 h and cells were exposed to 20 µg/mL **12** for additional 24 h. For acylated adenosine variants, additional co-treatment experiments were carried out using LPS. To this end, 20 µg/mL **16**, **17**, or adenosine was introduced to macrophages for 3 h and cells were stimulated with 100 ng/mL LPS for additional 24 h. After 24 h exposure of each treatment, cells were lysed and total RNA isolated using TRIzol (Thermo Fisher). Then, cDNA was synthesized using 3 µg of the total RNA extracted using the Superscript III cDNA Synthesis Kit (Invitrogen, Carlsbad, CA, USA) according to manufacturer protocols.

The real-time RT-PCR analysis was conducted using the iTaq Universal SYBR Green Supermix (Bio-Rad) on a CFX96 Real-Time PCR Detection System (Bio-Rad) consisting of a C1000 Thermal Cycler and a CFX96 Real-Time System. Each well, in a total volume of 16 µL, contained 1 µL of 2-fold diluted cDNA, 1 µL of each primer (final concentration of 0.625 µM), 8 µL of iTaq Universal SYBR Green Supermix, and 5 µL of nuclease-free water (Bio-rad). The PCR cycle consisted of a 1 min incubation at 95°C followed by 42 cycles of a 2-step amplification protocol (5 s at 95°C and 30 s at 58°C) with a final extension at 68°C for 10 min. Forward and reverse primer sequences used in this study were listed in Table S14. The $2^{-\Delta\Delta Ct}$ method was used to quantify the level of each transcript. β-actin was used as internal reference.

To measure gene transcript levels in *B. dorei* CL02T00C15 under DMC treatment, a frozen stock of *B. dorei* was inoculated into 10 mL GAM in an 18 mL anaerobic culture tube and incubated at 37°C for 2 d in the anaerobic chamber. The seed cultures were then used to inoculate 18 mL culture tubes each containing 10 mL of CDM at an initial OD_{600} of 0.05. DMC (4 µM) or a vehicle DMSO control was supplemented in triplicates and the cultures were incubated at 37°C for until cultures reached mid-exponential growth (~0.5–0.7 OD_{600}), which was at 8 and 17 h post inoculation for the vehicle- and DMC-treated samples, respectively. Then 1-mL of each was taken, centrifuged, and resuspended in 1 mL of RNAlater® and stored at 4°C until RNA isolation. RNA isolation was completed via the RNeasy kit (Qiagen), per manufacturer instructions. Contaminating genomic DNA was removed with the TURBO DNA-free kit (ThermoFisher), per manufacturer instructions. Quality of the RNA was assessed via gel electrophoresis. RNA was converted into cDNA using the iScript kit (BioRad) with 1 µg of RNA as the template.

RT-qPCR primers (Table S14) were designed to give an amplicon length of ~100 bp and melting temperatures at 59°C. Genomic DNA of *B. dorei* CL02T00C15 was isolated using the Wizard genomic DNA purification kit (Promega) and was used as a template to amplify PCR amplicon standards, which were subsequently purified. Standard curves for each amplicon were generated using 5 concentrations (from 2 pg/µL to 200 ag/µL, 10-fold successive dilutions) for quantification. qPCR was completed using a CFX96 real-time PCR detection system (BioRad) with the iTaq Universal SYBR green Supermix (BioRad) in technical triplicates. The PCR amplification cycle included a 1-min incubation (95°C) followed by 42 cycles of a two-step amplification (5 sec at 95°C, then 30 sec at 59°C). For each reaction, a single amplification species was observed. Quantification cycle (Cq) values were determined from triplicate experiments and then converted to copy number using the standard curve. The resulting value was normalized to the DMSO control sample and then further normalized to transcript levels of the housekeeping gene *rpoB* to give the fold change for each gene analyzed.

Measurements of TNFα, IL-1β, MCP-1, and CAMP by ELISA. To confirm the immunogenic effects of dipeptide lipids, the serum levels of TNFα, IL-1β, MCP-1, and CAMP produced by activated RAW 264.7 cells were determined using mouse TNFα, IL-1β, and MCP-1 ELISA kits (Abcam) as well as mouse CAMP ELISA kits (Antibodies.com). RAW cells were incubated at a density of 1 x 10^5 cells per well in 6-well plates overnight. Cells were then treated with 20 µg/mL dipeptide lipids (**10**–**12**) for 24 h. To examine the immunogenic effects of acylated adenosine variants, cells were pre-treated with 20 µg/mL **16**, **17**, or adenosine for 3 h and stimulated by 100 ng/mL LPS for additional 24 h. Cells with DMSO treatment served as the negative control and cells with 100 ng/mL LPS served as the positive control. After 24 h treatment, culture supernatants were used to analyze the serum levels of TNFα, IL-1β, MCP-1, and CAMP following the manufacturer's protocols.

Cathelicidin Susceptibility Assay. Antimicrobial activity was evaluated using the broth microdilution method in accordance with Clinical and Laboratory Standards Institute (CLSI) guidelines. *B. dorei*, *B. fragilis*, *B. vulgatus*, *C. perfringens*, *P.* sp., and *P. oris* were cultured anaerobically in GAM media at 37°C, while *S. aureus* and *E. faecalis* were grown aerobically in brain heart infusion (BHI) media at 37°C with shaking, and *S. agalactiae* in Todd-Hewitt media supplemented with 0.2% yeast extract at 37°C without shaking. Cathelicidin (BOC Sciences) stock was prepared at 1 mg/mL in water and tested at final concentrations ranging from 0.25 to 128 µg/mL. For anaerobic species, overnight cultures were adjusted to an initial OD_{600} of 0.02 in GAM in 96-well polypropylene plates, and plates were sealed with Breathe-Easy sealing membrane and incubated at 37°C for 48 h. Aerobic species were treated similarly using Mueller-Hinton broth, with shaking for 12 h (48 h for *S. agalactiae*). The MIC was defined as the lowest concentration of cathelicidin that inhibited visible bacterial growth. All assays were performed in biological duplicates.

Heterologous Expression of *glsA* and *glsB* in *E. coli* BL21(DE3)

Expression plasmids encoding codon optimized C-terminal hexahistidine tagged *Bacteroides dorei* CL02T00C15 GlsA (NCBI Reference Sequence: WP_008655575.1) and GlsB (NCBI Reference Sequence: WP_007834762.1) were synthesized and cloned into pET blank (AMP) and pET-28a(+) expression vectors, respectively, by Twist Biosciences (pET_*glsA* and pET28a_*glsB*) (see Supplemental Note for sequence details). Chemically competent *E. coli* BL21(DE3) cells were transformed with pET_*glsA*, pET28a_*glsB*, with both, or with pET-28a(+) and pET-15b(+) empty vectors as a control, with appropriate antibiotics on LB agar plates (50 µg mL^{-1} kanamycin for pET-28a(+) plasmids and 200 µg mL^{-1} ampicillin for pET_*glsA* and pET-15b(+)). Single colonies were picked and grown in LB media precultures (5 mL) overnight at 37°C and 200 rpm with the appropriate antibiotics. 250 µL of each preculture were used to inoculate 25 mL of LB media in 250 mL Erlenmeyer flasks supplemented with the appropriate antibiotics. The cultures were grown to mid-log phase (OD_{600} = ~0.6) at 37ºC and 200 rpm. At this point, the protein expression was induced through the addition of isopropyl-β-D-1-thiogalactopyranoside (IPTG) at a final concentration of 500 µM and the temperature was cooled to 18°C. After 20 hours of incubation, each culture was subject to an ethyl acetate extraction (1 x 25 mL), dried with Na_2SO_4, and concentrated *in vacuo.* The extracts were resuspended in 500 µL of MeOH, filtered, and was analyzed by HPLC-qTOF-MS. Separation of the heterologous products was achieved on an analytical Poroshell C18 column (Agilent Poroshell 120 139 SB-Aq 2.7 µm, 2.7 x 50 mm) operating at a flow rate of 0.5 mL/min and an elution gradient of 10% MeCN in H_2O to 100% MeCN over 12 min and then held at 100% MeCN FA for 3 min. The mobile phases contained 0.1% FA.

Bioinformatic Analyses of the *gls* BGC

Global identification of the *gls* BGC. To identify all candidate homologs of GlsB, a BLASTP search was conducted on the complete non-redundant protein database from NCBI (as of November 2023) using the GlsB protein from *B. dorei* CL02T00C15 (NCBI Reference Sequence: WP_007834762.1) with an e-value threshold of 0.001, which gave 29,504 hits. Hit sequences were clustered at 40% identity using CD-HIT [3], which afforded 519 clusters. Representative sequences of each cluster were used to generate a multiple sequence alignment using Clustal Omega [4]. The phylogenetic tree from this alignment was visualized in iTol [5]. Closest branches to the original query branch were manually investigated to determine if the *glsAB* cluster was present. The first clade of common ancestors that included non *glsAB* BGC members were collapsed and taken forward, which included 33 clusters. The sequences from all 33 clusters were expanded (6,492 nonredundant sequences) and re-clustered at 75% identity using CD-HIT, which gave 1,149 clusters. These sequences were trimmed to only include clusters whose representative sequence was over 300 amino acids in length, which gave 1,043 clusters. The representative sequences for each cluster were analyzed by RODEO webtool 2.0 to generate PFAM cooccurrence genome neighbor datasets [6]. Of the 1,043 sequences searched, neighborhood data was available for 841 sequences. Representative sequences and their neighbors were analyzed to determine whether each has a PFAM

match to PF13444 (Acetyltransferase 5 (GNAT)), which is the PFAM classification for GlsB, and/or a PFAM hit for PF19576 or PF01553 (1-acyl-sn-glycerol-3-phosphate acyltransferase), which are the PFAM classifications for GlsA. Representative GlsB sequences of the post-RODEO analysis clusters were used to generate a multiple sequence alignment using Clustal Omega and a phylogenetic tree was visualized from this alignment with iTol (Figure 5C). The *glsA* and *glsB* cooccurrence data and phylogeny were mapped in iTol. The tree was rooted with a non-GlsB ancestor from a Cyanobacteriota lineage (WP_193991399.1) and branch lengths were ignored for visual clarity.

Quantification of the *gls* BGC in reference Bacteroidales. Protein coding sequences for all 275 representative Bacteroidales were downloaded from NCBI (as of December 2023) and a custom protein BLAST database was created from these sequences. To identify all candidate homologs GlsB, a BLASTP search was conducted on the complete custom database using the GlsB protein from *B. dorei* CL02T00C15 (NCBI Reference Sequence: WP_007834762.1) with an e-value threshold of 0.001. Hits were analyzed using the RODEO webtool 2.0 to generate PFAM cooccurrence genome neighbor datasets as described above. 263 out of 272 (96.7%) representative Bacteroidales genomes contained the *glsAB* BGC (Table S15). Several sequences were omitted due to the low genome completeness, the presence of the *glsAB* homolog on a contig edge, or either *glsA* or *glsB* were predicted to be pseudogenes.

Doreamide production and elicitation in additional Bacteroidales strains. *Bacteroides* sp. 1_1_30 and *Bacteroides dorei* CL02T12C06 were inoculated from glycerol stocks into 10 mL GAM in an 18 mL anerobic culture tube and incubated at 37°C for 2 d in the anaerobic chamber. Cultures were inoculated into CDM and dosed with varying concentrations (0, 2, 4, and 6 µM) of demeclocycline, as described above. After 4 d, cultures were extracted twice with 15 mL of ethyl acetate. The organic phases were dried in vacuo, and the resulting samples were dissolved in 1 mL of MeOH for HPLC-qTOF-MS analysis as detailed in general procedure above.

Statistical Analysis

Statistical analysis was performed with the GraphPad Prism 9 software (GraphPad Software, San Diego, CA, USA). Two-sample comparisons were carried out using an unpaired two-tailed Student's *t* test and multiple comparisons performed using one-way ANOVA followed by *post hoc* test. Statistical differences were accepted at the 5% level.

Supplemental Note

Amino Acid and DNA Sequences

- GlsA with hexahistidine tag
    - MTDDSIFLIDIEKILKTKAGKKYKYIPRFVVSYLKRIVHQDELNVFLKDSKN
KVGVDFLEACMEFLDAKVEIKGLENLPENGKCTFVSNHPLGGQDGVALG
YILGKHYNGNVRYLVNDLLMNLHGLAPLCIPINKTGSQSRDFPKMVEAGF
ASDNHIIMFPAGLCSRRQGGEIKDLEWKKTFVTKSIETHRDVVPLHFEGR
NSDFFYNLANICKALGIKFNIAMLYLADEMLKNRHKTFTLTIGKPIPWQTF
DKTKTPAQWAQFVKDVVYKLHHHHHH*

- *glsA* (codon optimized)
    - ATGACAGACGATTCTATCTTCTTAATTGACATCGAGAAGATTCTTAAG ACTAAGGCTGGAAAGAAGTATAAGTATATTCCCCGCTTCGTAGTTTCG TATCTTAAGCGTATCGTACATCAGGACGAATTGAACGTATTCCTTAAG GACAGTAAAAACAAAGTGGGCGTCGACTTCCTGGAGGCTTGCATGG AATTTTTGGATGCGAAGGTGGAGATCAAGGGACTTGAGAACTTACCC GAAAACGGCAAGTGCACTTTTGTAAGCAATCATCCGCTTGGGGGTCA GGATGGTGTGGCATTAGGTTACATCTTGGGAAAACACTACAACGGAA ACGTGCGTTACTTGGTCAATGACTTGCTGATGAACTTGCACGGGCTG GCTCCGCTGTGTATTCCCATCAATAAGACGGGTAGCCAGTCGCGTGA TTTCCCCAAGATGGTAGAAGCTGGGTTTGCAAGTGATAATCATATTAT TATGTTCCCAGCCGGATTATGCAGTCGTCGCCAAGGCGGTGAGATTA AAGACTTGGAATGGAAGAAAACGTTCGTTACGAAGAGTATTGAAACC CACCGCGACGTCGTACCACTGCACTTCGAGGGCCGCAATTCAGACT TTTTCTATAACCTTGCGAATATCTGCAAGGCGCTTGGCATTAAGTTCA ACATTGCGATGCTGTATCTGGCGGACGAAATGCTTAAGAATCGTCAC AAAACTTTTACACTGACGATTGGGAAGCCAATCCCCTGGCAAACCTT CGACAAGACAAAGACGCCTGCACAATGGGCGCAGTTCGTGAAGGAC GTTGTATATAAGTTACACCACCACCACCACCACTGA

- GlsB with hexahistidine tag
    - MEEEIIAPISKEILKAELSEDKRLRFTNKSHNEIYVITHQDSPNVMKEIGRL REIAFRAAGGGTGKAMDIDEYDVMENPYKQLVVWNPEAEEILGGYRYLL GDEVQFDEHGKPLLATAHMFNFSEVFLKKYLPYTVELGRSFVTLEYQST RAGSKGLFALDNLWDGLGALTVIKPNVKYFFGKMTMYPSYHRQGRDMI LYFLNKHFGDKDKLITPMKPLEIETDKKMLENLFCYDSFKEDYKILNTEVR KLGYNIPPLVNAYMSLSPTMRMFGTAINYGFGDVEETGILIAVNEILEDKR VRHIESFVKQHPEALKITSGAHPILTKLEHHHHHH*
- *glsB* (codon optimized)
    - ATGGAAGAAGAGATCATCGCACCAATCTCGAAAGAAATCCTGAAGGC CGAGCTGTCTGAGGATAAGCGCTTGCGCTTCACGAACAAGAGCCAC AATGAAATCTATGTTATCACACACCAAGATAGCCCAAATGTTATGAAA GAGATCGGCCGCTTGCGTGAGATCGCGTTCCGTGCCGCTGGTGGTG GGACTGGTAAGGCTATGGACATTGACGAGTATGATGTCATGGAAAAT CCGTATAAACAACTTGTCGTGTGGAACCCCGAAGCTGAAGAAATCCT TGGTGGCTATCGCTATCTTCTGGGCGACGAAGTTCAGTTTGACGAAC ATGGGAAACCTCTGTTGGCTACTGCGCACATGTTCAACTTTAGCGAA GTATTTTTGAAGAAATATTTACCGTATACAGTGGAGTTGGGTCGTTCT TTTGTCACTCTGGAGTACCAGTCGACGCGTGCGGGAAGCAAAGGGC TGTTCGCTTTGGATAATCTGTGGGATGGATTAGGGGCGTTAACAGTA ATCAAGCCAAACGTCAAGTATTTTTTTGGGAAAATGACAATGTACCCG AGCTACCATCGCCAAGGGCGTGACATGATTTTATATTTCCTTAACAAG CACTTCGGCGACAAAGACAAATTGATTACACCAATGAAGCCCCTTGA

AATCGAAACAGATAAAAAGATGCTGGAAAATCTGTTCTGCTACGATAG
CTTCAAAGAGGATTATAAAATCCTGAATACGGAGGTACGCAAACTGG
GTTATAACATCCCACCGCTTGTTAATGCATACATGTCTCTTTCTCCTA
CAATGCGCATGTTTGGCACTGCGATCAATTACGGATTCGGGGATGTA
GAGGAGACAGGTATCCTTATCGCGGTCAACGAGATCCTGGAAGACA
AACGTGTGCGTCACATCGAGTCCTTTGTCAAACAACATCCTGAAGCC
CTGAAAATCACCAGCGGGGCCCATCCCATTTTAACAAAACTCGAGCA
CCACCACCACCACCACTGA

Table S1. A complete list of elicitors used for the *B. dorei* screen.

No.	Compound name	No.	Compound name
1	griseofulvin	49	bethanechol chloride
2	salsalate	50	bisacodyl
3	danthron	51	bithionate sodium
4	mequinol	52	bromocriptine mesylate
5	hydrocortisone	53	busulfan
6	desoxycorticosterone acetate	54	caffeine
7	testosterone propionate	55	camphor
8	sparteine sulfate	56	carbachol
9	salicylanilide	57	carbamazepine
10	phenyl aminosalicylate	58	carbenicillin disodium
11	testosterone	59	carbinoxamine maleate
12	sanguinarium chloride	60	carisoprodol
13	mitomycin	61	cefadroxil
14	sodium nitroprusside	62	cefazolin sodium
15	sodium oxybate	63	cefotaxime sodium
16	mannitol	64	cephalothin sodium
17	acetaminophen	65	cephapirin sodium
18	acetylcholine chloride	66	acetazolamide
19	acetylcysteine	67	cephradine
20	adenosine	68	cetylpyridinium chloride
21	allopurinol	69	chlorambucil
22	alverine citrate	70	chloramphenicol palmitate
23	amantadine hydrochloride	71	chloramphenicol sodium succinate
24	docetaxel	72	chloramphenicol
25	amiloride hydrochloride	73	norgestimate
26	potassium p-aminobenzoate	74	chlorhexidine dihydrochloride
27	aminocaproic acid hydrochloride	75	chlorocresol
28	aminoglutethimide	76	chlorothiazide
29	aminosalicylate sodium	77	chloroxylenol
30	amitriptyline hydrochloride	78	dexchlorpheniramine maleate
31	amodiaquine dihydrochloride	79	chlorpromazine
32	amoxicillin	80	chlorpropamide
33	amphotericin b	81	chlortetracycline hydrochloride
34	ampicillin sodium	82	chlorthalidone
35	anthralin	83	chlorzoxazone
36	antipyrine	84	ciclopirox olamine
37	apomorphine hydrochloride	85	cinoxacin
38	aspirin	86	clemastine fumarate
39	atropine sulfate	87	clidinium bromide
40	azathioprine	88	clindamycin hydrochloride
41	bacitracin	89	clomiphene citrate
42	beclomethasone dipropionate	90	clonidine hydrochloride
43	benserazide hydrochloride	91	clotrimazole
44	benzethonium chloride	92	cloxacillin sodium
45	benzocaine	93	cloxyquin
46	benzthiazide	94	colchicine
47	betamethasone	95	colistimethate sodium
48	betamethasone valerate		

No.	Compound name
96	cortisone acetate
97	cotinine
98	cresol
99	cromolyn sodium
100	cyclizine
101	cyclopentolate hydrochloride
102	cyclophosphamide
103	cycloserine (d)
104	cyproterone acetate
105	cytarabine
106	dacarbazine
107	danazol
108	dapsone
109	daunorubicin
110	deferoxamine mesylate
111	dehydrocholate sodium
112	demeclocycline hydrochloride
113	desipramine hydrochloride
114	dexamethasone
115	dexamethasone acetate
116	dexamethasone sodium phosphate
117	dextromethorphan hydrobromide
118	dibenzothiophene
119	dibucaine hydrochloride
120	diclofenac sodium
121	dicloxacillin sodium
122	dicyclomine hydrochloride
123	dienestrol
124	diethylcarbamazine citrate
125	diethylstilbestrol
126	diflunisal
127	digitoxin
128	digoxin
129	dihydroergotamine mesylate
130	dimenhydrinate
131	dimercaprol
132	dimethadione
133	dioxybenzone
134	diphenhydramine hydrochloride
135	diphenylpyraline hydrochloride
136	dipyridamole
137	pyrithione zinc
138	disulfiram
139	dopamine hydrochloride
140	doxepin hydrochloride
141	doxycycline hydrochloride
142	doxylamine succinate
143	dyclonine hydrochloride
144	dyphylline

No.	Compound name
145	ethylenediamine tetracetate tetra-ethanolamine
146	emetine dihydrochloride
147	ephedrine (1r,2s) hydrochloride
148	epinephrine bitartrate
149	domperidone
150	ergocalciferol
151	ergonovine maleate
152	erythromycin ethylsuccinate
153	erythromycin
154	estradiol
155	estradiol cypionate
156	estradiol valerate
157	estriol
158	estrone
159	ethacrynic acid
160	ethambutol hydrochloride
161	ethinyl estradiol
162	ethionamide
163	ethopropazine hydrochloride
164	eucalyptol
165	semustine
166	eugenol
167	fludrocortisone acetate
168	flumethazone pivalate
169	fluocinolone acetonide
170	fluocinonide
171	doramectin
172	fluorouracil
173	flurbiprofen
174	furazolidone
175	furosemide
176	fusidic acid
177	gallamine triethiodide
178	gemfibrozil
179	gentian violet
180	glucosamine hydrochloride
181	gramicidin (gramicidin a shown)
182	guaifenesin
183	guanabenz acetate
184	halazone
185	haloperidol
186	hetacillin potassium
187	hexachlorophene
188	hexylresorcinol
189	histamine dihydrochloride
190	homatropine hydrobromide
191	homatropine methylbromide
192	hydrochlorothiazide
193	hydrocortisone acetate

No.	Compound name
194	hydrocortisone hemisuccinate
195	hydrocortisone phosphate triethylamine
196	hydroflumethiazide
197	hydroxyprogesterone caproate
198	hydroxyurea
199	hydroxyzine pamoate
200	hyoscyamine
201	ibuprofen
202	imipramine hydrochloride
203	indapamide
204	indomethacin
205	canagliflozin
206	inositol
207	iodoquinol
208	ipratropium bromide
209	isoniazid
210	isopropamide iodide
211	isoproterenol hydrochloride
212	isosorbide dinitrate
213	isoxsuprine hydrochloride
214	kanamycin a sulfate
215	ketoconazole
216	lactulose
217	leucovorin calcium
218	levonordefrin
219	lincomycin hydrochloride
220	mafenide hydrochloride
221	maprotiline hydrochloride
222	mecamylamine hydrochloride
223	mechlorethamine
224	meclizine hydrochloride
225	meclofenamate sodium
226	medroxyprogesterone acetate
227	medrysone
228	megestrol acetate
229	melphalan
230	mepenzolate bromide
231	mercaptopurine
232	mestranol
233	metaproterenol
234	methacholine chloride
235	methenamine
236	methicillin sodium
237	methimazole
238	methocarbamol
239	methotrexate(+/-)
240	methoxamine hydrochloride
241	methoxsalen
242	methscopolamine bromide
243	methyldopa
244	methylergonovine maleate
245	methylprednisolone
246	methylthiouracil
247	metoclopramide hydrochloride
248	metoprolol tartrate
249	metronidazole
250	miconazole nitrate
251	minocycline hydrochloride
252	moxalactam disodium
253	nadide
254	nafcillin sodium
255	naloxone hydrochloride
256	naphazoline hydrochloride
257	naproxen(+)
258	neomycin trisulfate
259	neostigmine bromide
260	niacin
261	nifedipine
262	nitrofurantoin
263	nitrofurazone
264	nitromide
265	norepinephrine
266	norethindrone
267	norethindrone acetate
268	nortriptyline hydrochloride
269	norethynodrel
270	norfloxacin
271	norgestrel
272	noscapine hydrochloride
273	novobiocin sodium
274	nylidrin hydrochloride
275	nystatin
276	orphenadrine citrate
277	oxacillin sodium
278	oxidopamine hydrochloride
279	oxybenzone
280	oxymetazoline hydrochloride
281	oxyphenbutazone
282	oxyquinoline hemisulfate
283	oxytetracycline
284	papaverine hydrochloride
285	parachlorophenol
286	pargyline hydrochloride
287	penicillin g potassium
288	penicillin v potassium
289	phenacemide
290	phenazopyridine hydrochloride
291	phenelzine sulfate
292	phenindione

No.	Compound name
293	pheniramine maleate
294	phenolphthalein
295	phenylbutazone
296	phenylephrine hydrochloride
297	1s,2r-phenylpropanolamine hydrochloride
298	phenytoin sodium
299	apixaban
300	pilocarpine nitrate
301	pindolol
302	piperacillin sodium
303	piperazine
304	prednicarbate
305	polymyxin b sulfate
306	praziquantel
307	prazosin hydrochloride
308	prednisolone
309	prednisolone acetate
310	prednisone
311	primaquine phosphate
312	primidone
313	probenecid
314	procainamide hydrochloride
315	procaine hydrochloride
316	prochlorperazine edisylate
317	procyclidine hydrochloride
318	progesterone
319	promazine hydrochloride
320	promethazine hydrochloride
321	vidarabine
322	vinblastine sulfate
323	warfarin
324	xylometazoline hydrochloride
325	zomepirac sodium
326	merbromin
327	phenacetin
328	chloroquine diphosphate
329	phenylmercuric acetate
330	azelaic acid
331	strychnine sulfate
332	dicumarol
333	yohimbine hydrochloride
334	acebutolol hydrochloride
335	adenosine 5-monophosphate
336	ketotifen fumarate
337	betahistine hydrochloride
338	molsidomine
339	mycophenolic acid
340	indoprofen
341	albuterol
342	arecoline hydrobromide
343	captopril
344	cimetidine
345	nebivolol hydrochloride
346	hydrastine (1r, 9s)
347	lidocaine hydrochloride
348	phentolamine hydrochloride
349	nalidixic acid
350	butamben
351	cefaclor
352	iodipamide
353	levothyroxine
354	liothyronine
355	allantoin
356	althiazide
357	adenine
358	aminacrine
359	bekanamycin sulfate
360	budesonide
361	canrenoic acid, potassium salt
362	chenodiol
363	cholecalciferol
364	cyanocobalamin
365	cholesterol
366	piperine
367	etoposide
368	dehydrocholic acid
369	azatadine maleate
370	flumequine
371	flunarizine hydrochloride
372	fluphenazine hydrochloride
373	flutamide
374	droperidol
375	famotidine
376	etodolac
377	fenoterol hydrobromide
378	fenbufen
379	fenofibrate
380	fenoprofen
381	flufenamic acid
382	fenbendazole
383	fenspiride hydrochloride
384	mefenamic acid
385	methacycline hydrochloride
386	puromycin dihydrochloride
387	mefexamide hydrochloride
388	probucol
389	mebendazole
390	propoxycaine hydrochloride
391	mebeverine hydrochloride

No.	Compound name	No.	Compound name
392	meclocycline sulfosalicylate	397	tramiprosate
393	proglumide	398	atenolol
394	minaprine hydrochloride	399	capsaicin
395	memantine hydrochloride	400	carbetapentane citrate
396	aceclidine		

Table S2. Complete list of elicitors for compounds **10**, **16**, and **17** identified from the *B. dorei* screen. Fold changes were calculated by normalizing to the average mass intensity of elicitor-untreated controls.

elicitor	function/bioactivity	fold change
	m/z 417.29 (**10**)	
daunorubicin (**4**)	antineoplastic	2.14
demeclocycline (**1**)	antibacterial	5.09
dyclonine (**i**)	anesthetic	2.13
semustine (**ii**)	antineoplastic	1.65
eugenol (**iii**)	analgesic	1.62
fusidic acid (**5**)	antibacterial	3.50
mercaptopurine (**iv**)	antineoplastic	1.84
Methylthiouracil (**v**)	others	1.73
minocycline (**7**)	antibacterial	1.97
oxyquinoline (**vi**)	antifungal, anti-infective, antimalarial, antiviral	1.62
oxytetracycline (**2**)	antibacterial	2.38
meclocycline (**3**)	antibacterial	2.19
	m/z 352.16 (**16** and **17**)	
danthron (**8**)	cathartic	1.40
fusidic acid (**5**)	antibacterial	1.30
minocycline (**7**)	antibacterial	1.29
novobiocin (**9**)	antibacterial	1.79
methacycline (**6**)	antibacterial	1.38
puromycin (**vii**)	antineoplastic	1.21

Table S3. A list of representative induced metabolites by elicitors **1–5** in initial screen, besides **10**, **16**, and **17**. Fold changes were calculated relative to the average of three untreated control samples.

fold change	*m/z*				
elicitor (no.)	382.1568	268.0988	304.1563	298.1120	286.1727
daunorubicin (**4**)	1.18	0.42	3.34	1.20	1.61
demeclocycline (**1**)	3.86	8.75	2.51	1.99	2.90
fusidic acid (**5**)	3.66	8.65	3.76	2.32	3.62
oxytetracycline (**2**)	3.34	8.37	1.56	1.68	0.91
meclocycline (**3**)	3.44	8.38	1.37	1.40	1.06

Table S4. HR-MS data for *B. dorei* metabolites identified in this study. $[M+H]^+_{calc}$ and $[M+H]^+_{obs}$ denote calculated and observed $[M+H]^+$, respectively.

name (no.)	formula	$[M+H]^+_{calc}$	$[M+H]^+_{obs}$	Δppm
doreamide A (**10**)	$C_{21}H_{40}N_2O_6$	417.2959	417.2931	6.7
doreamide B (**11**)	$C_{22}H_{42}N_2O_6$	431.3116	431.3090	6.0
doreamide C (**12**)	$C_{22}H_{42}N_2O_6$	431.3116	431.3092	5.6
doreamide D (**13**)	$C_{20}H_{38}N_2O_6$	403.2803	403.2766	9.2
doreamide E (**14**)	$C_{23}H_{44}N_2O_6$	445.3272	445.3259	2.9
doreamide F (**15**)	$C_{24}H_{46}N_2O_6$	459.3429	459.3392	8.1
iso-branched 6-*N*-acyladenosine (**16**)	$C_{15}H_{21}N_5O_5$	352.1615	352.1611	1.1
anteiso-branched 6-*N*-acyladenosine (**17**)	$C_{15}H_{21}N_5O_5$	352.1615	352.1618	0.9
***conf*-16**	$C_{15}H_{21}N_5O_5$	352.1615	352.1616	0.3
***conf*-17**	$C_{15}H_{21}N_5O_5$	352.1615	352.1614	0.3

Table S5. ^{1}H and ^{13}C NMR assignments of doreamide A (**10**). "n.d." denotes not detected.

no.	**10**[a]	
	δ_C[b]	δ_H (J in Hz)[c]
**1**	n.d.	-
**2**	54.5	3.85 m
**3**	62.6	3.54; 3.29 dd (11.5, 5.5)
**NH**	-	7.44 s
**4**	168.5	-
**5**	42.4	3.66 d (6.0)
**NH**		8.22 t (6.0)
**6**	171.5	-
**7**	43.9	2.19 m
**8**	67.6	3.80 m
**9**	37.1	1.35 m
**10**	25.2	1.22; 1.33 m
**11–17**		overlapped
**18**	38.5	1.13 q (7.0)
**19**	27.5	1.49 dp (13.5, 6.5)
**20**	22.6	0.84 d (6.5)

[a]DMSO-d_6, [b]125 MHz, [c]500 MHz

doreamide A (**10**)

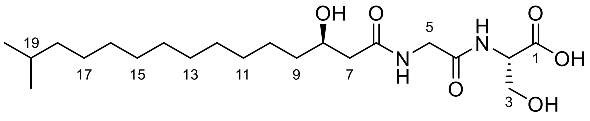

Table S6. Gibbs free energies and Boltzmann distribution of conformer **11a** (2*S*, 8*R*).

Conformers	B3LYP/6-31+G(d,p) Gibbs free energy (298.15K)		
	G (Hartree)	ΔG (kcal/mol)	Boltzmann distribution (%)
**1**	-2838.8638	0.00	**46.25933%**
**2**	-2838.8549	5.63	**0.00345%**
**3**	-2838.8520	7.43	**0.00016%**
**4**	-2838.8513	7.88	**0.00008%**
**5**	-2838.8503	8.52	**0.00003%**
**6**	-2838.8559	4.97	**0.01044%**
**7**	-2838.8510	8.05	**0.00006%**
**8**	-2838.8567	4.49	**0.02374%**
**9**	-2838.8596	2.69	**0.49765%**
**10**	-2838.8397	15.17	**0.00000%**
**11**	-2838.8360	17.48	**0.00000%**
**12**	-2838.8530	6.80	**0.00048%**
**13**	-2838.8541	6.09	**0.00159%**
**14**	-2838.8360	17.49	**0.00000%**
**15**	-2838.8629	0.60	**16.66218%**
**16**	-2838.8636	0.14	**36.54082%**

Table S7. Gibbs free energies and Boltzmann distribution of conformer **11b** (2*S*, 8*S*).

Conformers	B3LYP/6-31+G(d,p) Gibbs free energy (298.15K)		
	G (Hartree)	ΔG (kcal/mol)	Boltzmann distribution (%)
**1**	-2838.8657	0.00	**0.00011%**
**2**	-2838.8772	-7.17	**19.87150%**
**3**	-2838.8642	0.99	**0.00002%**
**4**	-2838.8646	0.71	**0.00003%**
**5**	-2838.8744	-5.40	**0.99974%**
**6**	-2838.8373	17.87	**0.00000%**
**7**	-2838.8578	4.97	**0.00000%**
**8**	-2838.8473	11.55	**0.00000%**
**9**	-2838.8450	13.00	**0.00000%**
**10**	-2838.8653	0.26	**0.00007%**
**11**	-2838.8392	16.67	**0.00000%**
**12**	-2838.8474	11.48	**0.00000%**
**13**	-2838.8582	4.74	**0.00000%**
**14**	-2838.8785	-7.98	**77.99120%**
**15**	-2838.8745	-5.47	**1.13733%**
**16**	-2838.8264	24.68	**0.00000%**
**17**	-2838.8593	4.02	**0.00000%**
**18**	-2838.8394	16.53	**0.00000%**
**19**	-2838.8521	8.54	**0.00000%**

Table S8. ^1H and ^{13}C NMR assignments of doreamides B and C (**11** and **12**).

no.	**11**[a]		no.	**12**[a]	
	δ_C[b]	δ_H (J in Hz)[c]		δ_C[b]	δ_H (J in Hz)[c]
**1**	172.6	-	**1**	173.0	-
**2**	54.8	3.98 q (6.0)	**2**	54.7	3.87 m
**3**	62.0	3.62; 3.44 dd (10.0, 5.0)	**3**	62.2	3.59; 3.38 dd (10.0, 5.0)
**NH**	-	7.66 d (6.5)	**NH**	-	7.54 d (6.5)
**4**	168.8	-	**4**	168.6	-
**5**	42.2	3.70 d (6.0)	**5**	42.3	3.68 d (6.0)
**NH**	-	8.14 t (6.0)	**NH**	-	8.19 t (6.0)
**6**	171.5	-	**6**	171.5	-
**7**	43.8	2.20 dd (6.5, 3.0)	**7**	43.8	2.20 dd (6.5, 3.0)
**8**	67.6	3.79 m	**8**	67.6	3.79 m
**9**	37.0	1.35 m	**9**	37.0	1.35 m
**10**	25.2	1.24; 1.35 m	**10**	25.2	1.24; 1.35 m
**11–18**		overlapped	**11–17**		overlapped
**19**	38.6	1.13 q (7.0)	**18**	36.1	1.24 m
**20**	27.9	1.49 dd (13.0, 6.5)	**19**	33.8	1.28 m
**21**	22.6	0.84 d (6.5)	**20**	29.0	1.09 m
			**21**	11.3	0.82 m
			**22**	19.2	0.82 t (7.0)

[a]DMSO-d_6, [b]125 MHz, [c]500 MHz

doreamide B (**11**)

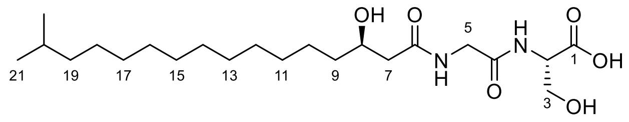

doreamide C (**12**)

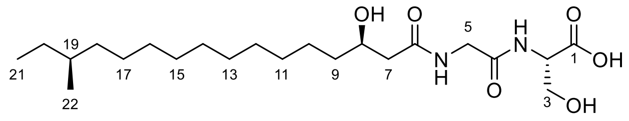

Table S9. ^{1}H and ^{13}C NMR assignments of doreamide E (**14**). "n.d." denotes not detected.

no.	**14**[a]	
	δ_C[b]	δ_H (J in Hz)[c]
**1**	n.d.	-
**2**	n.d.	3.70, m
**3**	70.4	3.51, m
**NH**	-	7.43, br s
**4**	-	n.d.
**5**	42.8	3.66, m
**NH**	-	8.19, br s
**6**	n.d.	-
**7**	44.3	2.19, m
**8**	67.8	3.79, m
**9**	37.7	1.35, m
**10–19**	30.0–27.1	1.26–1.19, m
**20**	38.9	1.13, m
**21**	27.9	1.49, m
**22**	23.0	0.84,[d] d (6.5)
**23**	23.0	0.84,[d] d (6.5)

[a]DMSO-d_6, [b]^{13}C chemical shifts determined by comprehensive analyses of ^{1}H, HSQC, and HMBC NMR spectra recorded at 500 MHz, [c]500 MHz, [d]Overlapped signals.

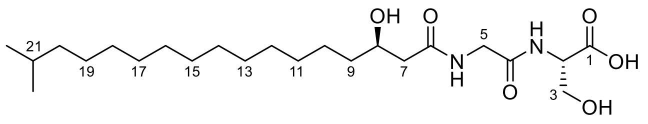

doreamide E (**14**)

Table S10. ^{1}H NMR and ^{13}C NMR assignments of compounds **16**.

no.	**16**[a]		no.	***conf*-16**[a]	
	δ_C[b]	δ_H (J in Hz)[c]		δ_C[b]	δ_H (J in Hz)[c]
**1**	-	-	**1**	-	-
**2**	152.8	8.14 s	**2**	152.8	8.14 s
**3**	-	-	**3**	-	-
**4**	149.5	-	**4**		-
**5**	119.7	-	**5**		-
**6**	156.8	-	**6**		-
**7**	-	-	**7**		-
**8**	140.4	8.37 s	**8**	140.4	8.37 s
**9**	-	-	**9**		
**1′**	88.2	5.87 d (7.2)	**1′**	85.9	6.14 d (7.2)
**2′**	72.1	4.90 m	**2′**	69.2	4.45 m
**3′**	73.7	5.30 dd (5.5, 2.0)	**3′**	75.3	5.63 dd (6.0, 5.2)
**4′**	84.2	4.11 m	**4′**	86.9	4.01 m
**5′**	62.2	3.68; 3.58 m	**5′**	62.2	3.68; 3.58 m
**NH**	-	7.38 s	**NH**		7.35 s
**1″**	172.0	-	**1″**		
**2″**	43.1	2.29 d (7.0)	**2″**	42.7	2.19 d (7.0)
**3″**	25.6	2.06 m	**3″**	25.5	1.91 m
**4″**	22.6; 22.6[d]	0.95 d (7.0)	**4″**	22.5; 22.5[d]	0.80 d (7.0)
**2′-OH**		5.80 br s			
**5′-OH**		5.66 br s	**5″**		

[a]DMSO-d_6, [b]^{13}C chemical shifts determined by comprehensive analyses of ^{1}H, HSQC, and HMBC NMR spectra that were recorded at 500 MHz, [c]500 MHz, [d]Overlapped signals.

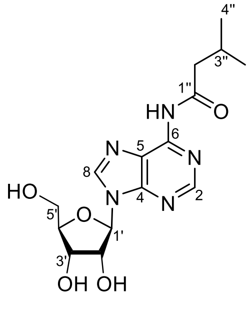

16

Table S11. ^{1}H NMR and ^{13}C NMR assignments of compounds **17**.

no.	**17**[a]		no.	***conf*-17**[a]	
	δ_C[b]	δ_H (*J* in Hz)[c]		δ_C[b]	δ_H (*J* in Hz)[c]
**1**	-		**1**	-	-
**2**	152.9	8.15 s	**2**	152.9	8.14 s
**3**	-		**3**	-	-
**4**	149.5		**4**	149.5	-
**5**	119.0		**5**	119.0	-
**6**	156.8		**6**	156.8	-
**7**	-		**7**	-	-
**8**	140.4	8.38 s	**8**	140.4	8.37 s
**9**	-		**9**	-	-
**1′**	88.2	5.88 d (7.0)	**1′**	86.0	6.14 d (5.5)
**2′**	72.0	4.92 dd (7.0, 5.5)	**2′**	75.4	5.61 dd (5.5, 5.5)
**3′**	73.8	5.29 dd (5.5, 2.0)	**3′**	69.2	4.46 dd (5.5, 5.5)
**4′**	84.2	4.10 m	**4′**	86.9	4.01 m
**5′**	62.1	3.68; 3.60 m	**5′**	62.1	3.68; 3.60 m
**NH**	-	7.37 s	**NH**	-	7.35 s
**1″**	175.5		**1″**	175.2	-
**2″**	40.7	2.51 m	**2″**	40.7	2.51 m
**3″**	26.7	1.48; 1.64 m	**3″**	26.5	1.38; 1.55 m
**4″**	11.9	0.91 t (7.0)	**4″**	11.9	0.79 t (7.0)
**5″**	16.8	1.13 d (7.0)	**5″**	16.7	1.00 d (7.0)
**5’-OH**		5.68 br s	**5’-OH**		5.48 br s

[a]DMSO-d_6, [b]^{13}C chemical shifts determined by comprehensive analyses of ^{1}H, HSQC, and HMBC NMR spectra recorded at 500 MHz, [c]500 MHz

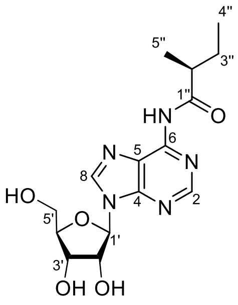

17

Table S12. Temperature-dependent changes in Major/minor ratio and $\Delta\delta_H$ of H_3-4′′ in variable-temperature ^1H NMR experiments of **16** in DMSO-d_6 at 500 MHz.

Temperature (K)	Major/Minor ratio	$\Delta\delta_H$ in Hz
298	11.12	78.87
308	10.53	77.29
318	8.50	76.15
328	6.00	75.00
338	3.44	72.70
348	2.55	70.97
358	2.28	69.70
368	2.26	68.25
378	2.17	66.82

Table S13. Major/minor ratio and $\Delta\delta_H$ of H_3-5′′ depending on temperature in variable-temperature ^1H NMR experiments of **17** in DMSO-d_6 at 500 MHz.

Temperature (K)	Major/Minor ratio	$\Delta\delta_H$ in Hz
298	10.31	66.85
308	9.36	66.09
318	8.23	65.15
328	6.90	64.32
338	5.36	63.11
348	2.85	62.17
358	2.41	61.05
368	2.31	60.08
378	2.20	59.38

Table S14. Primers used in this study [7-9].

primer	sequence (5'→3')
pro-inflammatory genes	
TNFα_F	CCCTCACACTCAGATCATCTTCT
TNFα_R	GCTACGACGTGGGCTACAG
IL-1β_F	GCCTTGGGCCTCAAAGGAAAGAATC
IL-1β _R	GGAAGACACAGATTCCATGGTGAAG
IL-6_F	TGGAGTCACAGAAGGAGTGGCTAAG
IL-6_R	TCTGACCACAGTGAGGAATGTCCAC
MCP-1_F	GGAAAAATGGATCCACACCTTGC
MCP-1_R	TCTCTTCCTCCACCACCATGCAG
MIP-1a_F	CCCAGCCAGGTGTCATTTTCC
MIP-1a_R	GCATTCAGTTCCAGGTCAGTG
MIP-2a_F	TCCAGAGCTTGAGTGTGACG
MIP-2a_R	TCAGGTACGATCCAGGCTTC
anti-inflammatory genes	
IL-10_F	CCAAGCCTTATCGGAAATGA
IL-10_R	TTTTCACAGGGGAGAAATCG
mediator or regulator genes	
Cox-2_F	CAGCAAATCCTTGCTGTTCC
Cox-2_R	TGGGCAAAGAATGCAAACATC
iNOS_F	GTCAACTGCAAGAGAACGGAGAAC
iNOS_R	GAGCTCCTCCAGAGGGTAGGCT
TLR2_F	CTCCTGAAGCTGTTGCGTTAC
TLR2_R	GCTCCCTTACAGGCTGAGTTC
TLR4_F	TCGCCTTCTTAGCAGAAACAC
TLR4_R	GCCTTAGCCTCTTCTCCTTC
housekeeping gene	
β-actin_F	CCTAAGGCCAACCGTGAAAA
β-actin_R	TCTTCATGGTGCTAGGAGCCA
B. dorei genes	
*glsA*_F	CGTAGACAGGGTGGGGAAAT
*glsA*_R	TGCAAAGGTACCACATCACGAT
*glsB*_F	GGGAAATAGCCTTTCGTGCT
*glsB*_R	ATTCCATACAACTAATTGCTTATAGGGAT
*bcat*_F	TTGGAACTTTGTTCGGAGGAAA
*bcat*_R	ACGATAAGCTTTCAACCCTTCAAA
*rpoB*_F	TTCTGACGGAAACGTCCTTGCTTA
*rpoB*_R	GATAACTCACGTAAATCAAGTGCAGTGA

Table S15. HR-MS data for *E. coli* heterologous GL and *bis*-GL products derived from *glsAB*. $[M+H]^+_{calc}$ and $[M+H]^+_{obs}$ denote calculated and observed $[M+H]^+$, respectively.

name	putative formula	$[M+H]^+_{calc}$	$[M+H]^+_{obs}$	Δppm
GL-1	$C_{16}H_{30}NO_4$	300.2169	300.2139	-10.0
GL-2	$C_{16}H_{32}NO_4$	302.2326	302.2297	-9.6
GL-3	$C_{18}H_{34}NO_4$	328.2482	328.2477	-1.5
GL-4	$C_{18}H_{36}NO_4$	330.2639	330.2621	-5.5
GL-5	$C_{20}H_{38}NO_4$	356.2795	356.2793	-0.6
bis-GL-1	$C_{28}H_{54}NO_5$	484.3997	484.3990	-1.4
bis-GL-2	$C_{30}H_{56}NO_5$	510.4153	510.4145	-1.6
bis-GL-3	$C_{30}H_{58}NO_5$	512.4310	512.4302	-1.6
bis-GL-4	$C_{32}H_{58}NO_5$	536.4310	536.4297	-2.4
bis-GL-5	$C_{32}H_{60}NO_5$	538.4466	538.4455	-2.0
bis-GL-6	$C_{34}H_{62}NO_5$	564.4623	564.4607	-2.8
bis-GL-7	$C_{34}H_{64}NO_5$	566.4779	566.4762	-3.0
bis-GL-8	$C_{36}H_{66}NO_5$	592.4936	592.4912	-4.0
bis-GL-9	$C_{36}H_{68}NO_5$	594.5092	594.5070	-3.7
bis-GL-10	$C_{38}H_{70}NO_5$	620.5249	620.5220	-4.7

Table S16. Analysis of reference Bacteroidales for the *glsAB* cluster.

bacterial species	*glsAB* cluster (Y/N)	RefSeq assembly ID
Acetobacteroides hydrogenigenes	Y	GCF_004340205.1
Alistipes communis	Y	GCF_006542665.1
Alistipes dispar	Y	GCF_006542685.1
Alistipes finegoldii	Y	GCF_027677115.1
Alistipes hominis	Y	GCF_014288115.1
Alistipes ihumii AP11	Y	GCF_025144665.1
Alistipes indistinctus	Y	GCF_014163495.1
Alistipes inops	Y	GCF_000786575.1
Alistipes megaguti	Y	GCF_900604385.1
Alistipes montrealensis	Y	GCF_018362775.1
Alistipes muris	Y	GCF_943193205.1
Alistipes onderdonkii subsp. *vulgaris*	Y	GCF_006542645.1
Alistipes provencensis	Y	GCF_900083545.1
Alistipes putredinis DSM 17216	Y	GCF_000154465.1
Alistipes senegalensis	Y	GCF_900624755.1
Alistipes shahii	Y	GCF_015558405.1
Alistipes timonensis JC136	Y	GCF_900107675.1
Alloprevotella rava	Y	GCF_014195585.1
Alloprevotella tannerae ATCC 51259	Y	GCF_000159995.1
Bacteroides acidifaciens	Y	GCF_910575425.1
Bacteroides bouchesdurhonensis	Y	GCF_900155865.1
Bacteroides caccae CL03T12C61	Y	GCF_018292205.1
Bacteroides caecigallinarum	Y	GCF_016902245.1
Bacteroides caecimuris	Y	GCF_001688725.2
Bacteroides cellulolyticus	Y	GCF_025567135.1
Bacteroides cellulosilyticus	Y	GCF_018292125.1
Bacteroides clarus	Y	GCF_015555825.1
Bacteroides congonensis	Y	GCF_900130125.1
Bacteroides coprosuis DSM 18011	Y	GCF_000212915.1
Bacteroides cutis	Y	GCF_900241005.1
Bacteroides difficilis	Y	GCF_014287385.1
Bacteroides eggerthii	Y	GCF_016894205.1
Bacteroides faecalis	Y	GCF_003865075.1
Bacteroides faecichinchillae	Y	GCF_900129065.1
Bacteroides faecis	Y	GCF_020091505.1
Bacteroides faecium	Y	GCF_012113595.1
Bacteroides finegoldii	Y	GCF_008571395.1

Bacteroides fluxus YIT 12057	Y	GCF_000195635.1
Bacteroides fragilis	Y	GCF_016889925.1
Bacteroides gallinaceum	Y	GCF_030373775.1
Bacteroides gallinarum DSM 18171 = JCM 13658	Y	GCF_000374365.1
Bacteroides graminisolvens	Y	GCF_029265535.1
Bacteroides helcogenes P 36-108	Y	GCF_000186225.1
Bacteroides heparinolyticus	Y	GCF_002998535.1
Bacteroides hominis (ex Liu et al. 2022)	Y	GCF_014288095.1
Bacteroides humanifaecis	Y	GCF_017309675.2
Bacteroides ihuae	Y	GCF_900104585.1
Bacteroides ilei	Y	GCF_900128495.1
Bacteroides intestinalis	Y	GCF_020341675.1
Bacteroides koreensis	Y	GCF_007341375.1
Bacteroides kribbi	Y	GCF_007341395.1
Bacteroides luhongzhouii	Y	GCF_009193295.2
Bacteroides luti	Y	GCF_900128905.1
Bacteroides mediterraneensis	Y	GCF_900128455.1
Bacteroides ndongoniae	Y	GCF_900108345.1
Bacteroides neonati	Y	GCF_902375225.1
Bacteroides nordii	Y	GCF_019930665.1
Bacteroides oleiciplenus	Y	GCF_902374815.1
Bacteroides ovatus	Y	GCF_001314995.1
Bacteroides parvus	Y	GCF_014287575.1
Bacteroides propionicifaciens DSM 19291 = JCM 14649	Y	GCF_000375405.1
Bacteroides propionicigenes	Y	GCF_018390535.1
Bacteroides pyogenes	Y	GCF_008121405.1
Bacteroides reticulotermitis	Y	GCF_014196955.1
Bacteroides rhinocerotis	Y	GCF_027942795.1
Bacteroides rodentium JCM 16496	–[b]	GCA_000614125.1
Bacteroides salyersiae	Y	GCF_025914135.1
Bacteroides stercorirosoris	Y	GCF_900142015.1
Bacteroides stercoris ATCC 43183	Y	GCF_000154525.1
Bacteroides thetaiotaomicron	Y	GCF_014131755.1
Bacteroides timonensis	Y	GCF_000513195.1
Bacteroides togonis	Y	GCF_900130135.1
Bacteroides uniformis CL03T12C37	Y	GCF_018292165.1
Bacteroides xylanisolvens	Y	GCF_018289135.1
Bacteroides zhangwenhongii	Y	GCF_009193325.2
Bacteroides zoogleoformans	Y	GCF_002998435.1

Balneicella halophila	Y	GCF_003096835.1
Barnesiella intestinihominis YIT 11860	Y	GCF_000296465.1
Barnesiella propionica	Y	GCF_025567045.1
Barnesiella viscericola DSM 18177	Y	GCF_000512915.1
Butyricimonas faecalis	Y	GCF_003991565.1
Butyricimonas faecihominis	Y	GCF_014196975.1
Butyricimonas hominis	Y	GCF_014287455.1
Butyricimonas paravirosa	Y	GCF_014647355.1
Butyricimonas synergistica DSM 23225	Y	GCF_000379665.1
Butyricimonas virosa	Y	GCF_016889065.1
Caecibacteroides pullorum	Y	GCF_019239235.1
Candidatus Alistipes pullistercoris	Y	GCF_027665465.1
Candidatus Azobacteroides pseudotrichonymphae genomovar. CFP2	Y	GCF_000010645.1
Candidatus Bacteroides intestinigallinarum	Y	GCF_024793055.1
Candidatus Symbiothrix dinenymphae	–[a]	GCF_001431085.1
Coprobacter fastidiosus NSB1 = JCM 33896	Y	GCF_003634345.1
Coprobacter secundus subsp. *similis*	Y	GCF_015097275.1
Coprobacter tertius	Y	GCF_024330105.1
Culturomica massiliensis	Y	GCF_900091655.1
Duncaniella dubosii	Y	GCF_004803915.1
Duncaniella freteri	Y	GCF_004766125.1
Duncaniella muricolitica	Y	GCF_910574735.1
Duncaniella muris	Y	GCF_003024805.1
Dysgonomonas alginatilytica	Y	GCF_003201355.1
Dysgonomonas capnocytophagoides	Y	GCF_004405045.1
Dysgonomonas gadei ATCC BAA-286	Y	GCF_000213555.1
Dysgonomonas hofstadii	Y	GCF_014196915.1
Dysgonomonas macrotermitis	Y	GCF_900128985.1
Dysgonomonas massiliensis	Y	GCF_900240225.1
Dysgonomonas mossii DSM 22836	Y	GCF_000213575.1
Gabonibacter massiliensis	Y	GCF_001487125.1
Gallalistipes aquisgranensis	Y	GCF_014982715.1
Hallella absiana	Y	GCF_025457545.1
Hallella bergensis DSM 17361	Y	GCF_000160535.1
Hallella colorans	Y	GCF_003096815.1
Hallella faecis	Y	GCF_018789675.1
Hallella mizrahii	Y	GCF_009695775.1
Hallella multisaccharivorax DSM 17128	Y	GCF_000218235.1
Hallella seregens ATCC 51272	Y	GCF_000518545.1

Heminiphilus faecis	Y	GCF_008728965.1
Hoylesella buccalis	Y	GCF_020735565.1
Hoylesella enoeca	Y	GCF_001444445.1
Hoylesella loescheii DSM 19665 = JCM 12249 = ATCC 15930	Y	GCF_000378085.1
Hoylesella marshii DSM 16973 = JCM 13450	Y	GCF_000146675.1
Hoylesella nanceiensis	Y	GCF_019375915.1
Hoylesella oralis ATCC 33269	Y	GCF_000185145.2
Hoylesella pleuritidis F0068	Y	GCF_000468135.1
Hoylesella saccharolytica F0055	Y	GCF_000318195.2
Hoylesella shahii	Y	GCF_937990675.1
Hoylesella timonensis	Y	GCF_002894165.1
Jilunia laotingensis	Y	GCF_014385165.1
Lascolabacillus massiliensis	Y	GCF_001282625.1
Lentimicrobium saccharophilum	Y	GCF_001192835.1
Lepagella muris	N	GCF_004793975.1
Leyella lascolaii	Y	GCF_900079775.1
Leyella stercorea DSM 18206	Y	GCF_000235885.1
Macellibacteroides fermentans	Y	GCF_013409575.1
Marseilla massiliensis	Y	GCF_023336985.1
Massilibacteroides vaginae	Y	GCF_900178525.1
Mediterranea massiliensis	Y	GCF_900128475.1
Metaprevotella massiliensis	–[a]	GCF_900241015.1
Microbacter margulisiae	N	GCF_014192515.1
Millionella massiliensis	Y	GCF_900104655.1
Muribaculum caecicola	Y	GCF_004801635.1
Muribaculum gordoncarteri	Y	GCF_004803695.1
Muribaculum intestinale	Y	GCF_001688845.2
Odoribacter laneus YIT 12061	Y	GCF_000243215.1
Odoribacter lunatus	Y	GCF_943193135.1
Odoribacter splanchnicus DSM 20712	Y[c]	GCF_020736405.1
Palleniella intestinalis	Y	GCF_013166595.1
Palleniella muris	Y	GCF_004792655.1
Paludibacter jiangxiensis	N	GCF_001618385.1
Paludibacter propionicigenes WB4	N	GCF_000183135.1
Parabacteroides acidifaciens	Y	GCF_003363715.1
Parabacteroides bouchesdurhonensis	Y	GCF_900186615.1
Parabacteroides chartae	Y	GCF_900168155.1
Parabacteroides chinchillae	Y	GCF_900108035.1
Parabacteroides chongii	Y	GCF_029581355.1

Parabacteroides distasonis	Y	GCF_012273055.1
Parabacteroides faecalis	Y	GCF_022809355.1
Parabacteroides faecis	Y	GCF_014199665.1
Parabacteroides goldsteinii	Y	GCF_020091425.1
Parabacteroides gordonii	Y	GCF_020297465.1
Parabacteroides hominis	Y	GCF_014287585.1
Parabacteroides johnsonii	Y	GCF_020735865.1
Parabacteroides massiliensis	Y	GCF_900155425.1
Parabacteroides merdae CL03T12C32	Y	GCF_000307345.1
Parabacteroides pacaensis	Y	GCF_900292045.1
Parabacteroides provencensis	Y	GCF_900232875.1
Parabacteroides segnis	Y	GCF_014287545.1
Parabacteroides timonensis	Y	GCF_900128505.1
Paramuribaculum intestinale	N	GCF_003024815.1
Paraprevotella clara	Y	GCF_902388125.1
Paraprevotella xylaniphila YIT 11841	Y	GCF_900683745.1
Perlabentimonas gracilis	Y	GCF_011174675.1
Petrimonas mucosa	Y	GCF_900095795.1
Phocaeicola abscessus CCUG 55929	Y	GCF_000312445.1
Phocaeicola barnesiae DSM 18169 = JCM 13652	Y	GCF_000374585.1
Phocaeicola coprocola	Y	GCF_027688575.1
Phocaeicola coprophilus	Y	GCF_016888945.1
Phocaeicola dorei	Y	GCF_902387545.1
Phocaeicola faecalis	Y	GCF_021730445.1
Phocaeicola faecicola	Y	GCF_013618865.1
Phocaeicola faecium	Y	GCF_014837055.1
Phocaeicola fibrisolvens	Y	GCF_025567125.1
Phocaeicola intestinalis	Y	GCF_014837065.1
Phocaeicola massiliensis B84634 = Timone 84634 = DSM 17679 = JCM 13223	Y	GCF_000382445.1
Phocaeicola oris	Y	GCF_021295095.1
Phocaeicola paurosaccharolyticus JCM 15092	Y	GCF_000613805.1
Phocaeicola plebeius	Y	GCF_902374375.1
Phocaeicola salanitronis DSM 18170	Y	GCF_000190575.1
Phocaeicola sartorii	Y	GCF_013358205.1
Phocaeicola vulgatus	Y	GCF_020885855.1
Porphyromonas asaccharolytica DSM 20707	Y	GCF_000212375.1
Porphyromonas bennonis DSM 23058 = JCM 16335	N	GCF_000375645.1

Porphyromonas bobii	Y	GCF_943169685.1
Porphyromonas cangingivalis	Y	GCF_900638305.1
Porphyromonas canoris	Y	GCF_000765975.1
Porphyromonas catoniae F0037	Y	GCF_000318215.2
Porphyromonas circumdentaria	Y	GCF_900167105.1
Porphyromonas crevioricanis	Y	GCF_900476255.1
Porphyromonas endodontalis	Y	GCF_900454815.1
Porphyromonas gingivalis ATCC 33277	Y	GCF_000010505.1
Porphyromonas gingivicanis	Y	GCF_000769135.1
Porphyromonas gulae	Y	GCF_000765945.1
Porphyromonas levii	Y	GCF_018206105.1
Porphyromonas loveana	Y	GCF_003096695.1
Porphyromonas macacae DSM 20710 = JCM 13914	Y	GCF_000379945.1
Porphyromonas pasteri	Y	GCF_014647755.1
Porphyromonas somerae	Y	GCF_022845615.1
Porphyromonas uenonis DSM 23387 = JCM 13868	Y	GCF_000482365.1
Prevotella amnii CRIS 21A-A	Y	GCF_000177355.1
Prevotella aurantiaca	Y	GCF_015262605.1
Prevotella bivia DSM 20514	Y	GCF_000262545.1
Prevotella brunnea	Y	GCF_008016795.1
Prevotella communis	Y	GCF_022024095.1
Prevotella conceptionensis 9403948	Y	GCF_000312305.1
Prevotella corporis DSM 18810 = JCM 8529	Y	GCF_000430525.1
Prevotella dentalis	Y	GCF_000242335.1
Prevotella dentasini JCM 15908	Y	GCF_000614065.1
Prevotella denticola	Y	GCF_018128205.1
Prevotella disiens	Y	GCF_900454955.1
Prevotella falsenii DSM 22864 = JCM 15124	Y	GCF_000613945.1
Prevotella fusca JCM 17724	Y	GCF_001262015.1
Prevotella herbatica	Y	GCF_017347605.1
Prevotella histicola	Y	GCF_018128125.1
Prevotella ihumii	Y	GCF_900155655.1
Prevotella illustrans	Y	GCF_017426725.1
Prevotella intermedia ATCC 25611	Y	GCF_001953955.1
Prevotella jejuni	Y	GCF_002849795.1
Prevotella koreensis	Y	GCF_003977605.1
Prevotella lacticifex	Y	GCF_019973475.1
Prevotella marseillensis	Y	GCF_900625065.1
Prevotella melaninogenica	Y	GCF_000144405.1

Prevotella merdae	Y	GCF_900290275.1
Prevotella micans F0438	Y	GCF_000243035.1
Prevotella multiformis	Y	GCF_018127985.1
Prevotella nigrescens	Y	GCF_018127825.1
Prevotella pallens	Y	GCF_900454935.1
Prevotella pectinovora	Y	GCF_000833995.1
Prevotella phocaeensis	Y	GCF_900065875.1
Prevotella scopos JCM 17725	Y	GCF_001683355.1
Prevotella veroralis	Y	GCF_018127765.1
Prevotella vespertina	Y	GCF_009728485.1
Prevotellamassilia timonensis	Y	GCF_900106785.1
Proteiniphilum acetatigenes DSM 18083	Y	GCF_000380985.1
Proteiniphilum propionicum	Y	GCF_022267555.1
Proteiniphilum saccharofermentans	Y	GCF_900095135.1
Pseudoprevotella muciniphila	Y	GCF_003265305.2
Rikenella microfusus DSM 15922	N	GCF_000427365.1
Salinivirga cyanobacteriivorans	Y	GCF_001443605.1
Sangeribacter muris	N	GCF_910574005.1
Sanguibacteroides justesenii	Y	GCF_003204205.1
Segatella albensis DSM 11370 = JCM 12258	Y	GCF_000426565.1
Segatella baroniae F0067	Y	GCF_000468635.1
Segatella bryantii	Y	GCF_022024215.1
Segatella buccae	Y	GCF_900454855.1
Segatella cerevisiae	Y	GCF_024054555.1
Segatella copri DSM 18205	Y	GCF_020735445.1
Segatella hominis	Y	GCF_004535825.1
Segatella maculosa OT 289	Y	GCF_000243015.1
Segatella oris	Y	GCF_900637655.1
Segatella oulorum F0390	Y	GCF_000224615.1
Segatella paludivivens DSM 17968 = JCM 13650	Y	GCF_000373185.1
Segatella salivae F0493	Y	GCF_000477535.1
Seramator thermalis	N	GCF_004138125.1
Sodaliphilus pleomorphus	Y	GCF_009676955.1
Tannerella forsythia 92A2	Y	GCF_000238215.1
Tannerella serpentiformis	Y	GCF_003033925.1
Tenuifilum thalassicum	Y	GCF_013265555.1
Tidjanibacter massiliensis	Y	GCF_900104605.1
Williamwhitmania taraxaci	Y	GCF_900096565.1
Xylanibacter brevis P6B11	Y	GCF_000621825.1

Xylanibacter caecicola	Y	GCF_013166515.1
Xylanibacter muris	Y	GCF_013166605.1
Xylanibacter oryzae DSM 17970	Y	GCF_000585355.1
Xylanibacter rarus	Y	GCF_001275135.1
Xylanibacter rodentium	Y	GCF_013166575.1
Xylanibacter ruminicola	Y	GCF_018389405.1
**Summary**		(263/272) = 96.7%

[a]Sequence was omitted due to low genome completeness, or the *glsA* homolog was found on a contig edge. [b]Either *glsA* or *glsB* is predicted to be a pseudogene. [c]The *glsAB* genes are fused.

Figure S1. Compound library used in this study to activate the secondary metabolome of *B. dorei*. The bioactivities of chemicals within the library have been categorized based on information provided by the vendor and are presented in the pie chart below.

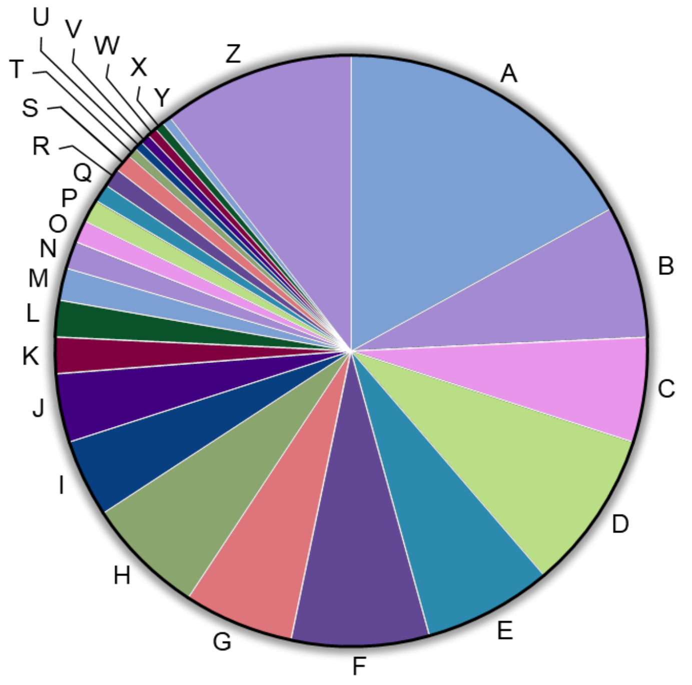

- A, 68, antibacterial
- B, 29, antiinfective, antifungal, antimalarial, antiviral
- C, 23, antineoplastic
- D, 35, anti-inflammatory, glucocorticoid
- E, 28, antihistaminic, anticholinergic, antitussive
- F, 30, antihypertensive, diuretic, antianginal
- G, 24, antidepressant, anticonvulsant, antipsychotic, antiemetic
- H, 26, androgen, estrogen, progestogen, mineralocorticoid, oxytocic, contraceptive,
- I, 17, bronchodilator, vasodilator, vasoconstrictor
- J, 15, analgesic, narcotic, antagonist
- K, 8, anesthetic
- L, 8, muscle relaxant
- M, 7, adrenergic agonist
- N, 6, stimulant (cardiac, CNS, gastric secretion, gonad, etc)
- O, 5, cholinergic
- P, 5, vitamin
- Q, 4, anthelmintic
- R, 4, anticoagulant
- S, 4, cathartic, laxative
- T, 2, antiarrhythmic
- U, 2, antiasthmatic
- V, 2, antidiabetic
- W, 2, antiglaucoma
- X, 2, antihyperlipidemic
- Y, 2, choleretic
- Z, 42, others (chelating agent, etc)

Figure S2. Chemical structures of potential inducers of **10** (*m/z* 417.29) identified by UPLC-MS-guided HiTES.

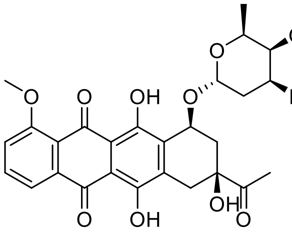

daunorubicin (**4**)

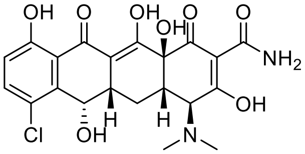

demeclocycline (**1**)

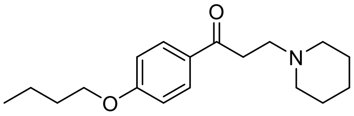

dyclonine (**i**)

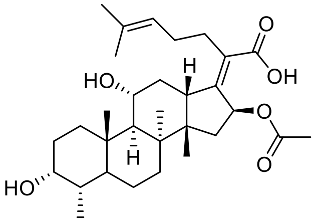

fusidic acid (**5**)

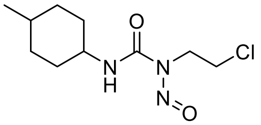

semustine (**ii**)

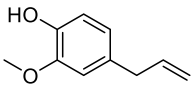

eugenol (**iii**)

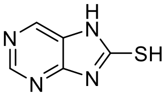

mercaptopurine (**iv**)

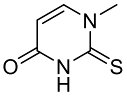

methylthiouracil (**v**)

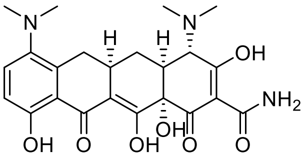

minocycline (**7**)

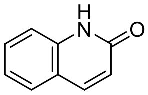

oxyquinoline (**vi**)

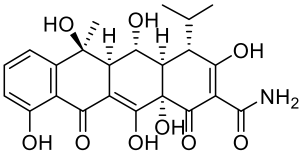

oxytetracycline (**2**)

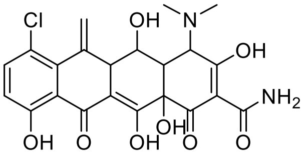

meclocycline (**3**)

Figure S3. Identification of additional doreamide derivatives induced by DMC. (A–D) Dose-dependent induction of additional compounds with *m/z* of (A) 431.31 (compound **11**), (B) 431.31 (**12**), (C) 445.33 (**14**), and (D) 459.34 (**15**). *, **, and *** denote difference between control and indicated point at $p<0.05$, $p<0.01$, and $p<0.001$, respectively.

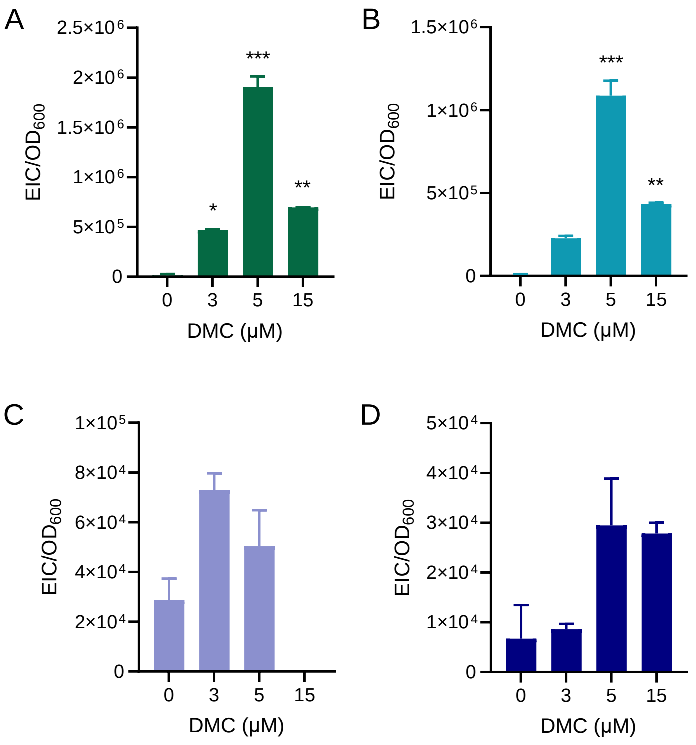

Figure S4. Chemical structures of potential inducers of two metabolites with *m/z* 352.16 (**16** and **17**) identified by UPLC-MS-guided HiTES.

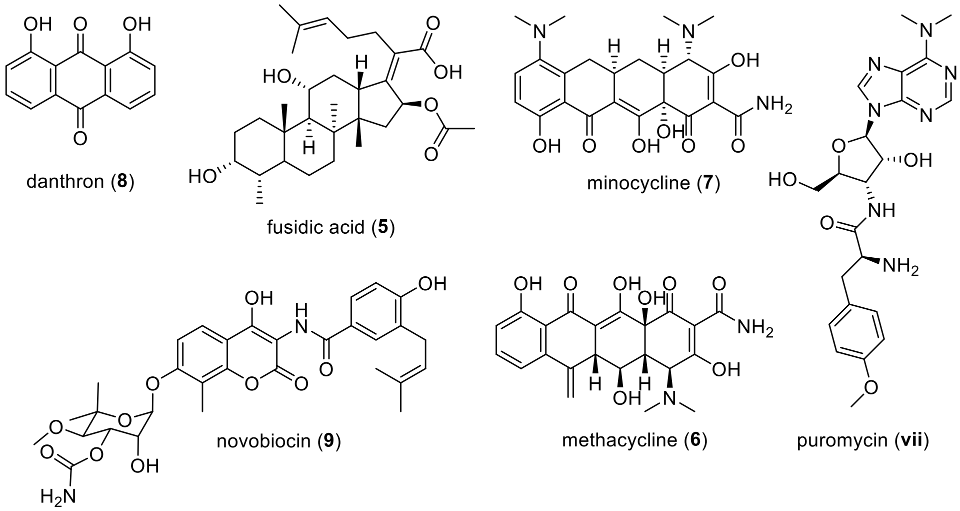

Figure S5. 1D and 2D NMR spectra of **10** (*m/z* 417.29) in DMSO-d_6.

A ^{1}H NMR

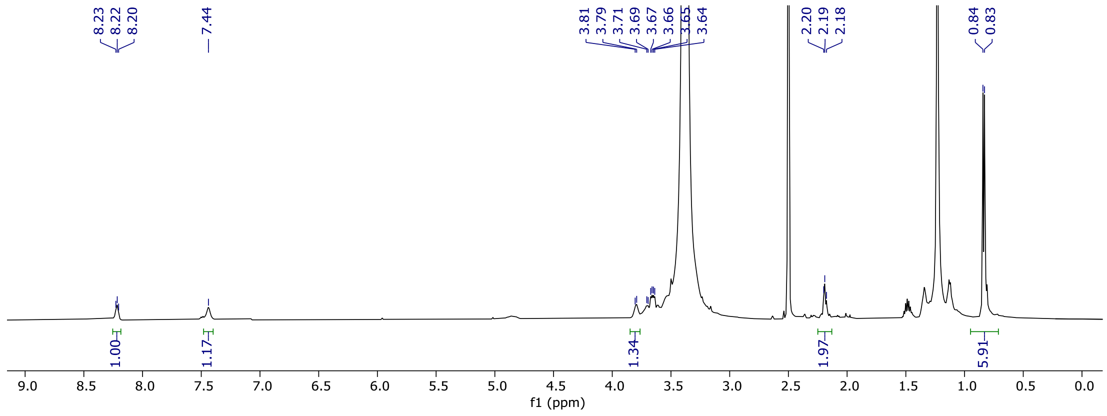

B ^{13}C NMR

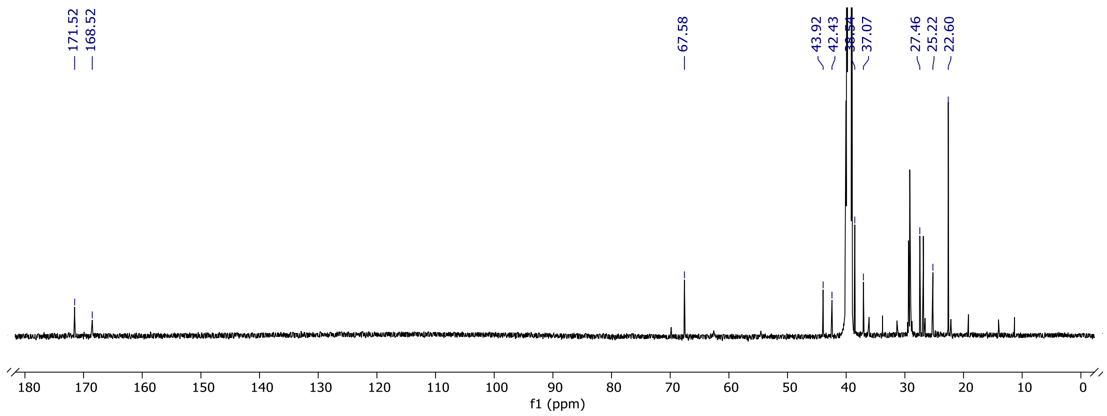

C HSQC

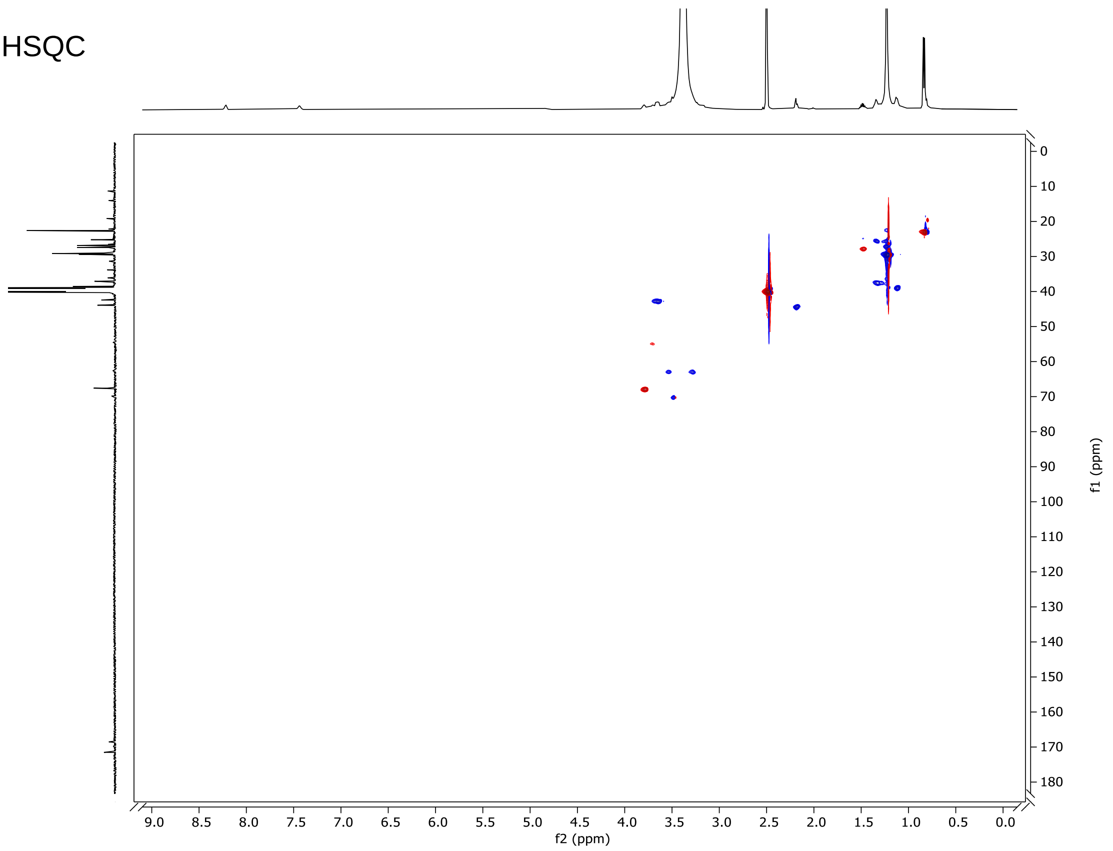

9.0 8.5 8.0 7.5 7.0 6.5 6.0 5.5 5.0 4.5 4.0 3.5 3.0 2.5 2.0 1.5 1.0 0.5 0.0
f2 (ppm)
0 10 20 30 40 50 60 70 80 90 100 110 120 130 140 150 160 170 180
f1 (ppm)

D HMBC

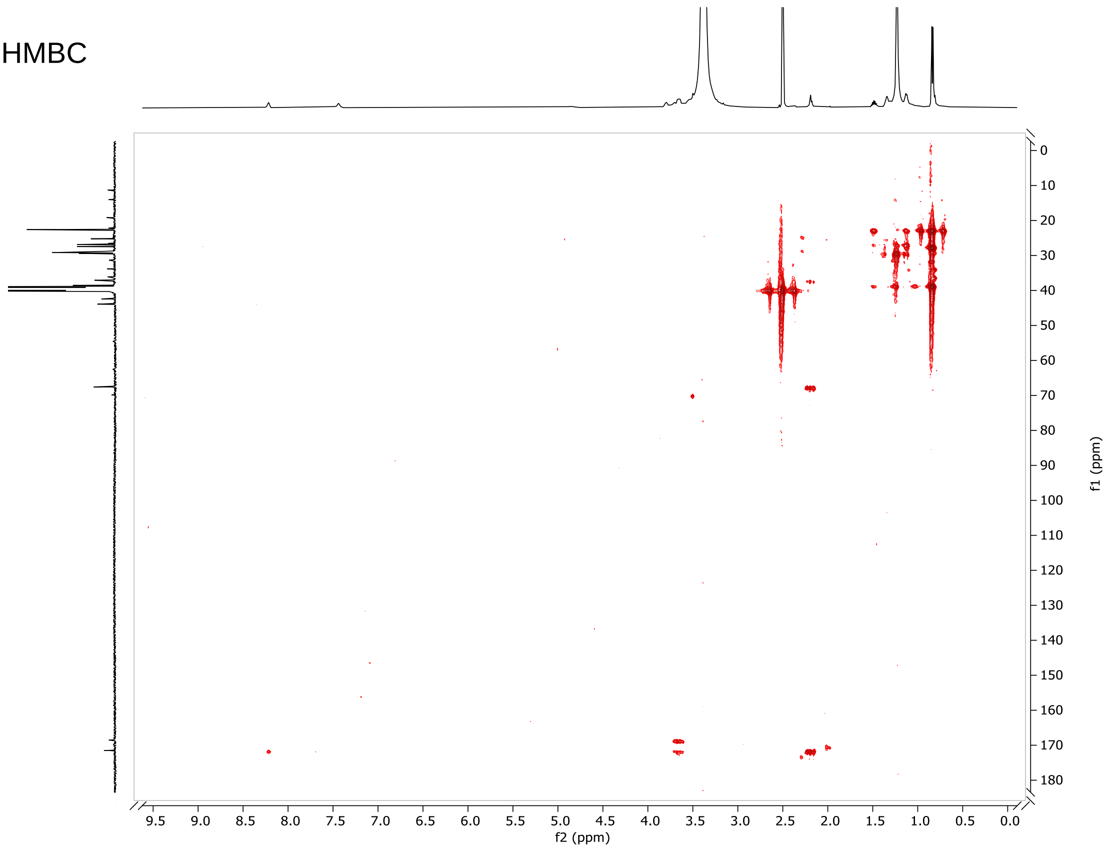

9.5 9.0 8.5 8.0 7.5 7.0 6.5 6.0 5.5 5.0 4.5 4.0 3.5 3.0 2.5 2.0 1.5 1.0 0.5 0.0
f2 (ppm)
0 10 20 30 40 50 60 70 80 90 100 110 120 130 140 150 160 170 180
f1 (ppm)

E gCOSY

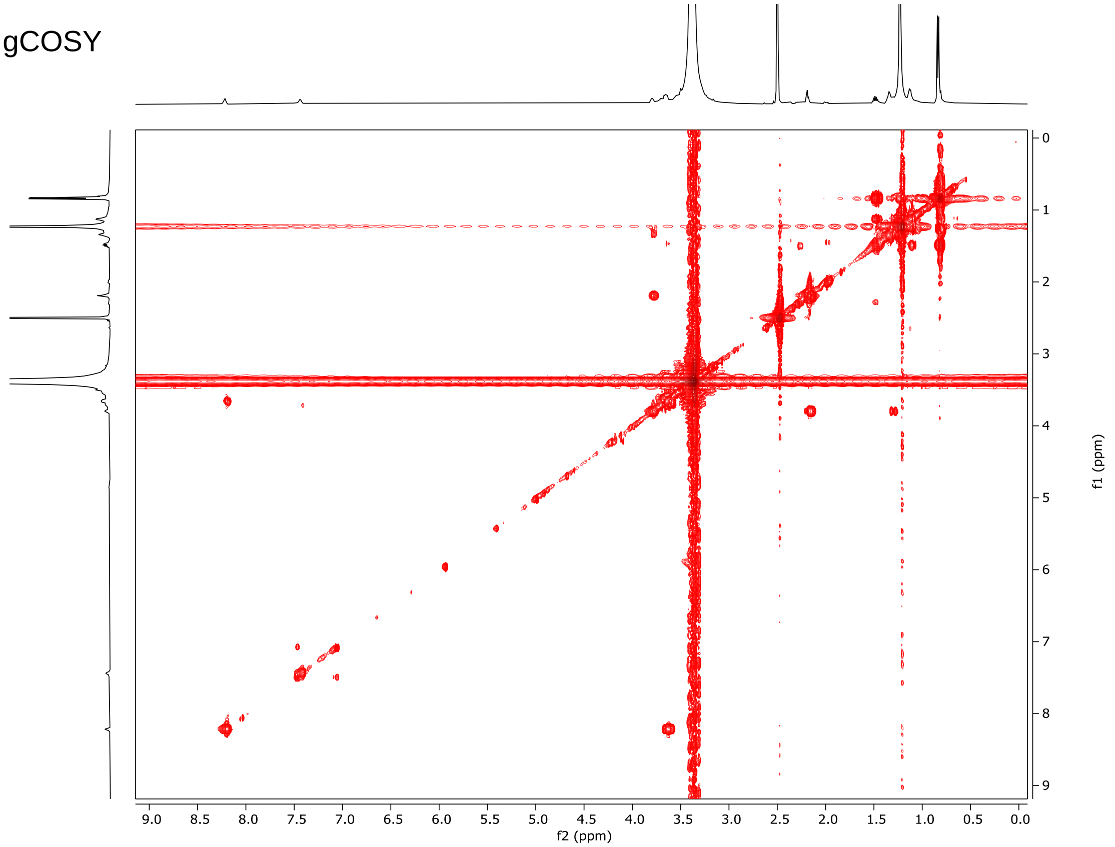

F NOESY

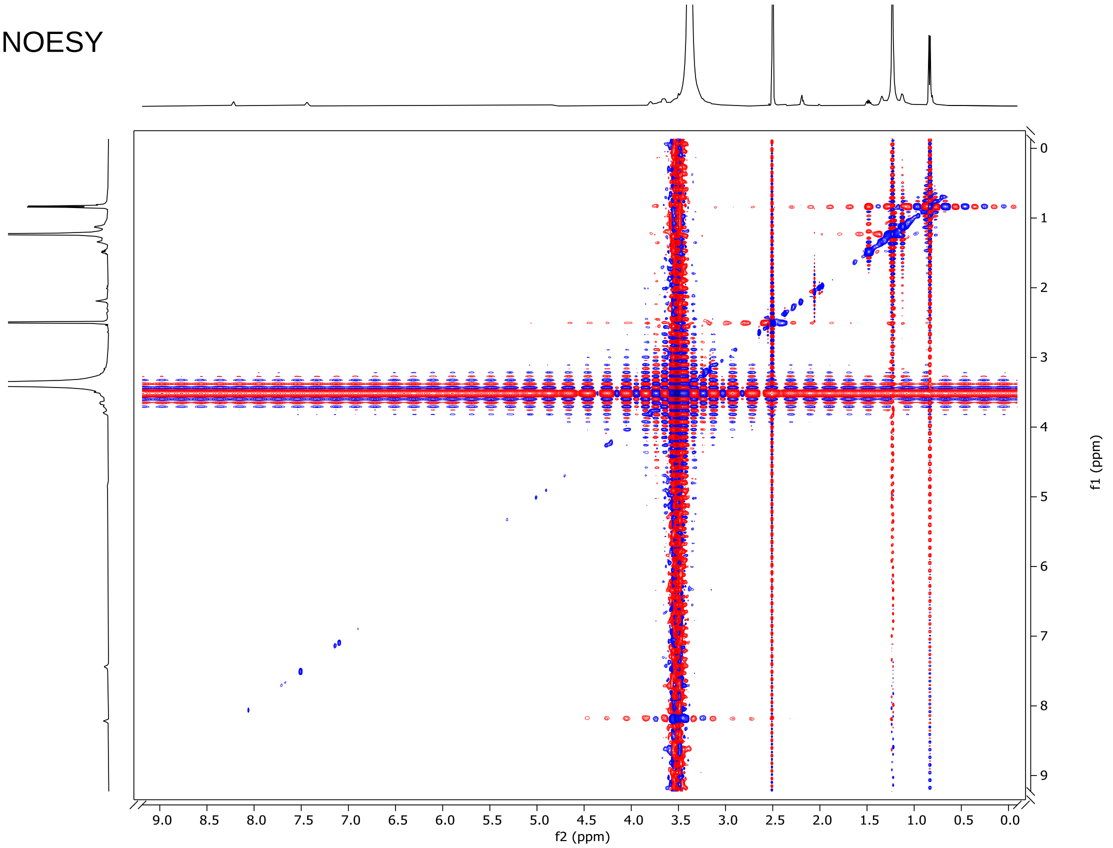

Figure S6. HR-MS/MS data for doreamides A–F (**10–15**). (A–F) Collision-induced ion loss of 164.07, 145.06, and 106.05 was observed from (A) **10** (*m/z* 417.29), (B) **11** (*m/z* 431.31), (C) **12** (*m/z* 431.31), (D) **13** (*m/z* 403.28), (E) **14** (*m/z* 445.33), and (F) **15** (*m/z* 459.34).

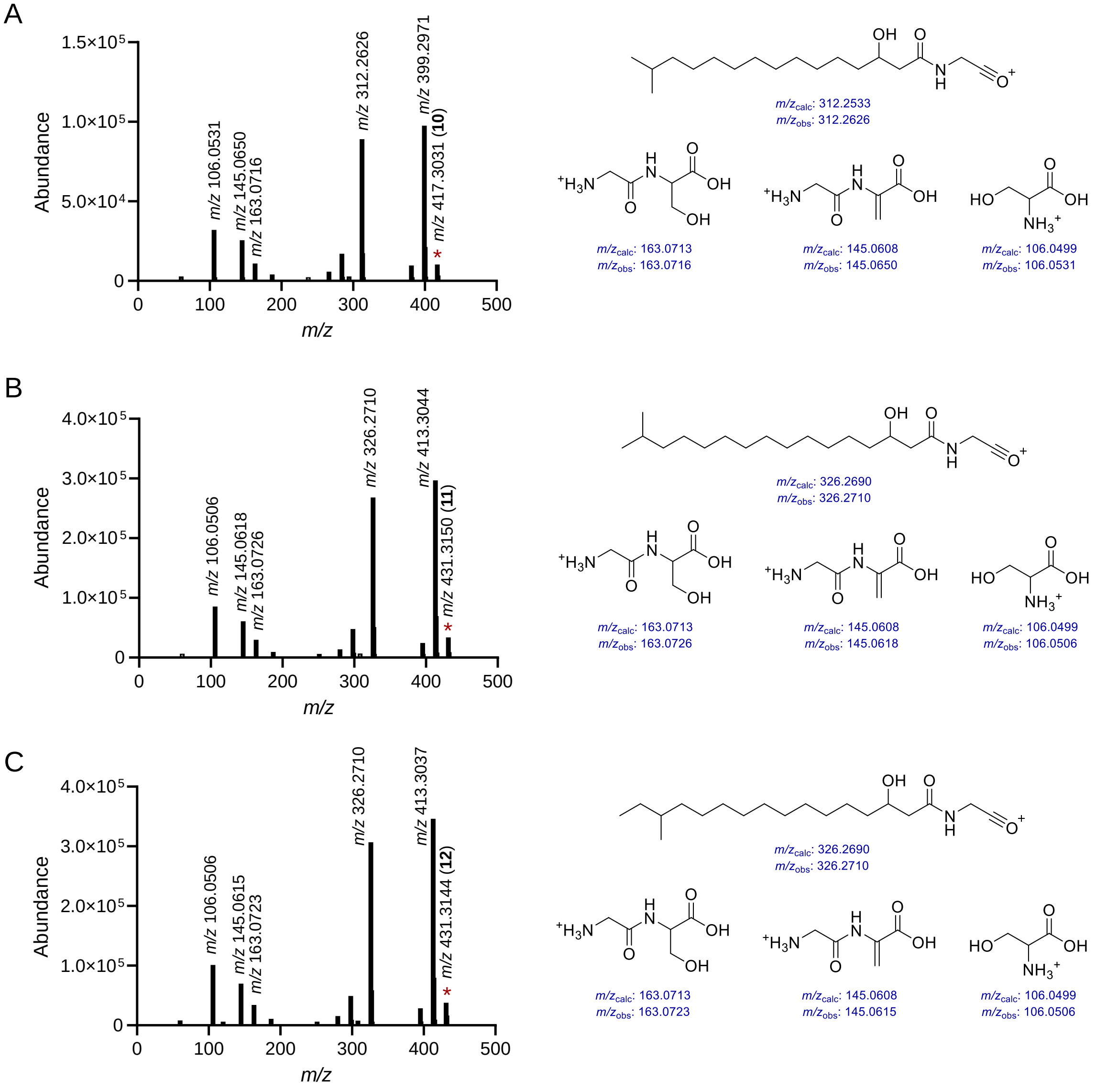

D

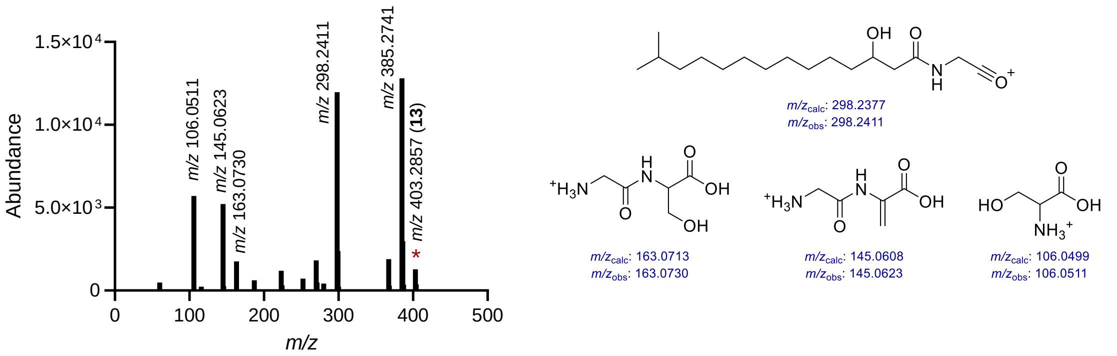

Abundance
1.5×10^4
1.0×10^4
5.0×10^3
0
m/z
m/z 106.0511
m/z 145.0623
m/z 163.0730
m/z 298.2411
m/z 385.2741
m/z 403.2857 (13)
*
m/z_calc: 298.2377
m/z_obs: 298.2411
m/z_calc: 163.0713
m/z_obs: 163.0730
m/z_calc: 145.0608
m/z_obs: 145.0623
m/z_calc: 106.0499
m/z_obs: 106.0511

E

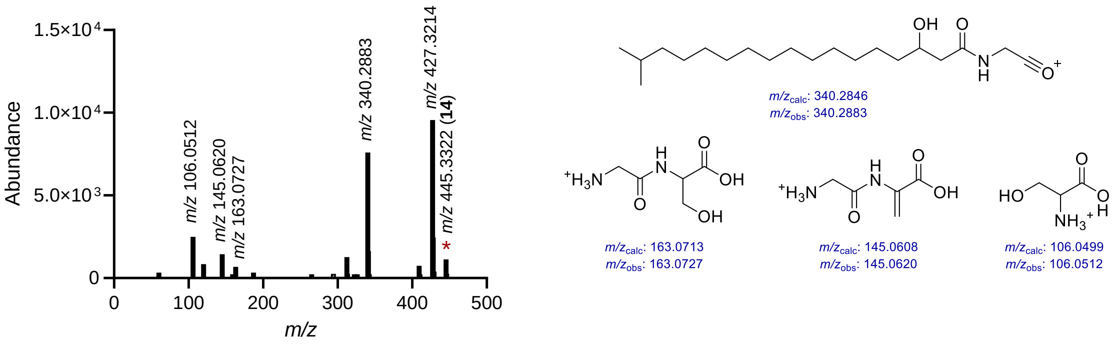

Abundance
1.5×10^4
1.0×10^4
5.0×10^3
0
m/z
m/z 106.0512
m/z 145.0620
m/z 163.0727
m/z 340.2883
m/z 427.3214
m/z 445.3322 (14)
*
m/z_calc: 340.2846
m/z_obs: 340.2883
m/z_calc: 163.0713
m/z_obs: 163.0727
m/z_calc: 145.0608
m/z_obs: 145.0620
m/z_calc: 106.0499
m/z_obs: 106.0512

F

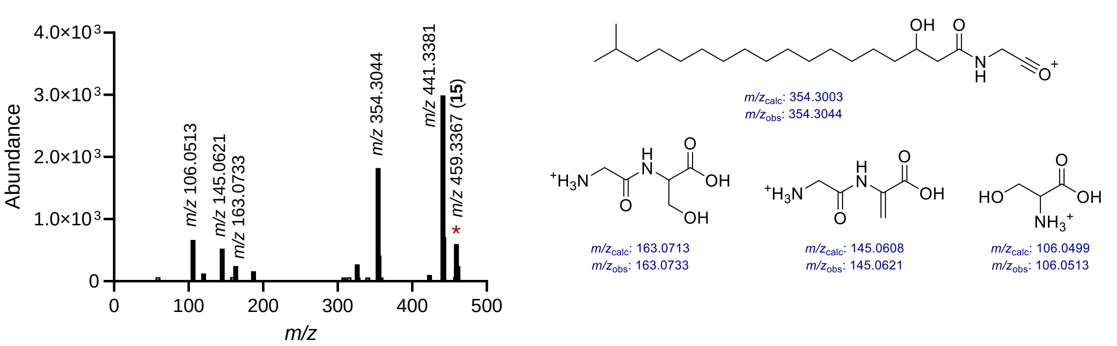

Abundance
4.0×10^3
3.0×10^3
2.0×10^3
1.0×10^3
0
m/z
m/z 106.0513
m/z 145.0621
m/z 163.0733
m/z 354.3044
m/z 441.3381
m/z 459.3367 (15)
*
m/z_calc: 354.3003
m/z_obs: 354.3044
m/z_calc: 163.0713
m/z_obs: 163.0733
m/z_calc: 145.0608
m/z_obs: 145.0621
m/z_calc: 106.0499
m/z_obs: 106.0513

Figure S7. Determination of absolute configuration of Ser in dipeptide lipid derivatives (**10–12**) using advanced Marfey's method followed by HPLC-qTOF-MS analysis. Shown are extracted ion chromatograms of the Ser-FDLA adduct (*m/z* 400.14) of hydrolyzed, FDLA-derivatized **10–12** as well as those of Ser standards.

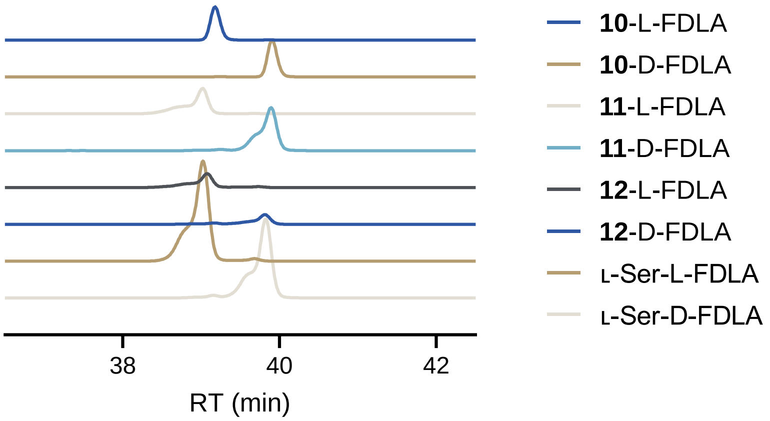

Figure S8. Experimental ECD spectra of dipeptide lipid derivatives (**10–12**) and calculated ECD data of **11a** (2*S*, 8*R*) and **11b** (2*S*, 8*S*).

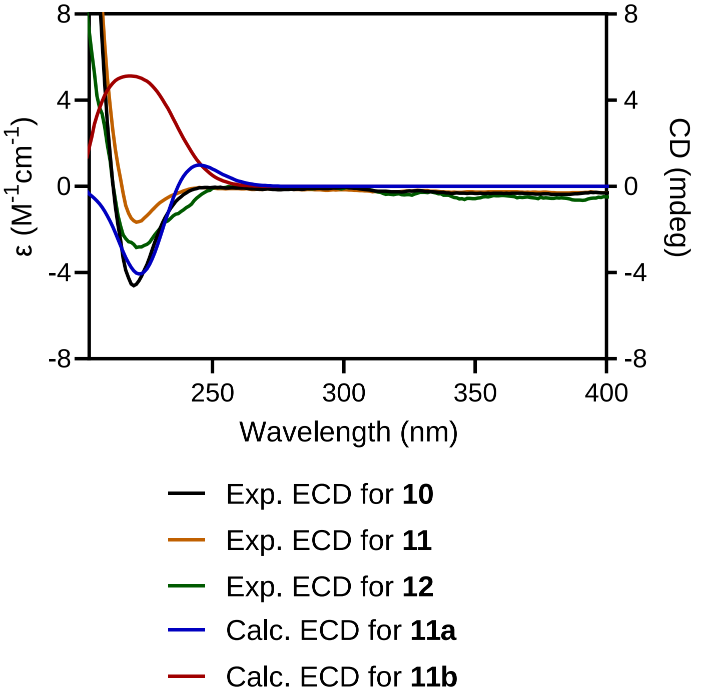

Figure S9. 1D and 2D NMR spectra of **11** (*m/z* 431.31) in DMSO-d_6.

A ^{1}H NMR

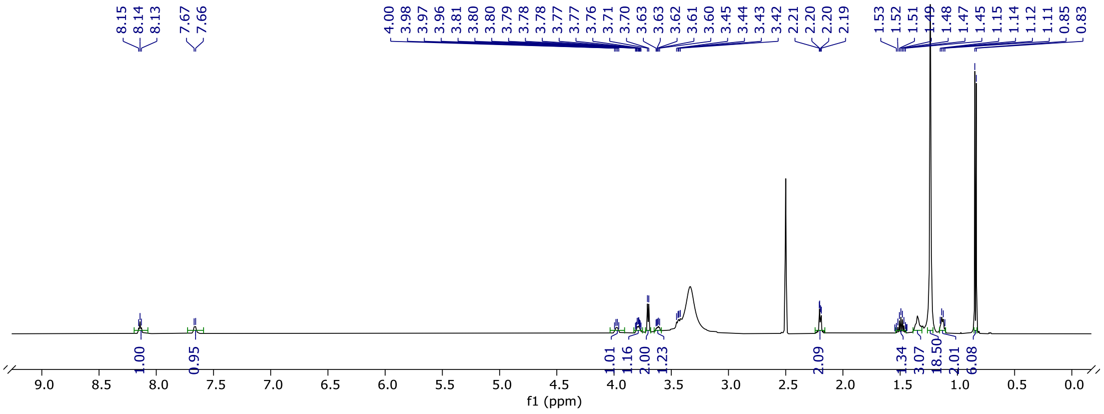

B ^{13}C NMR

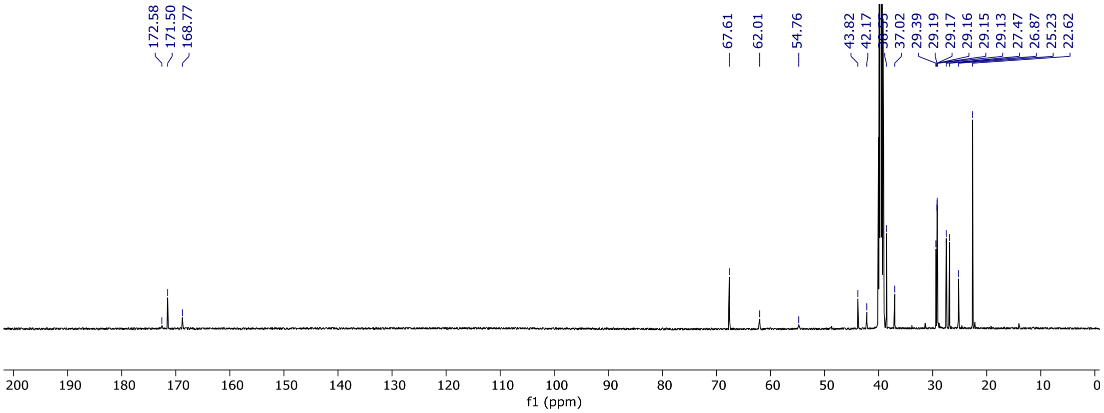

C HSQC

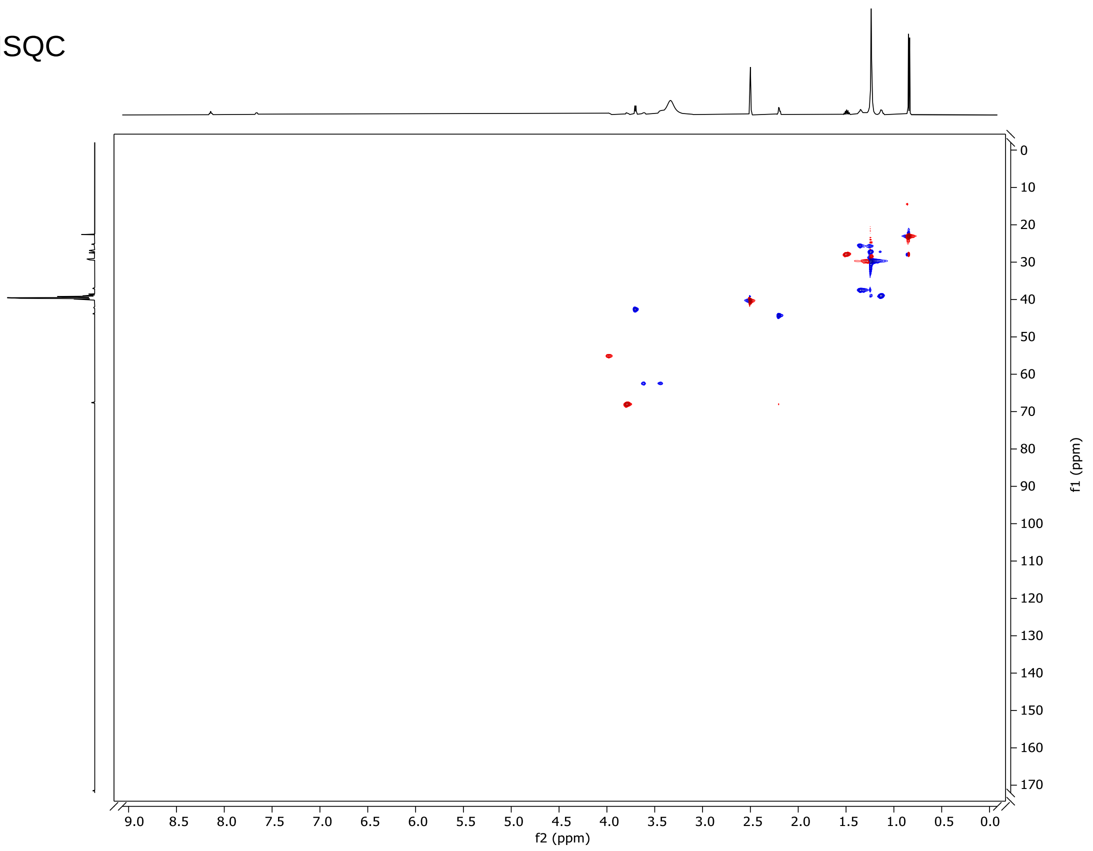

D HMBC

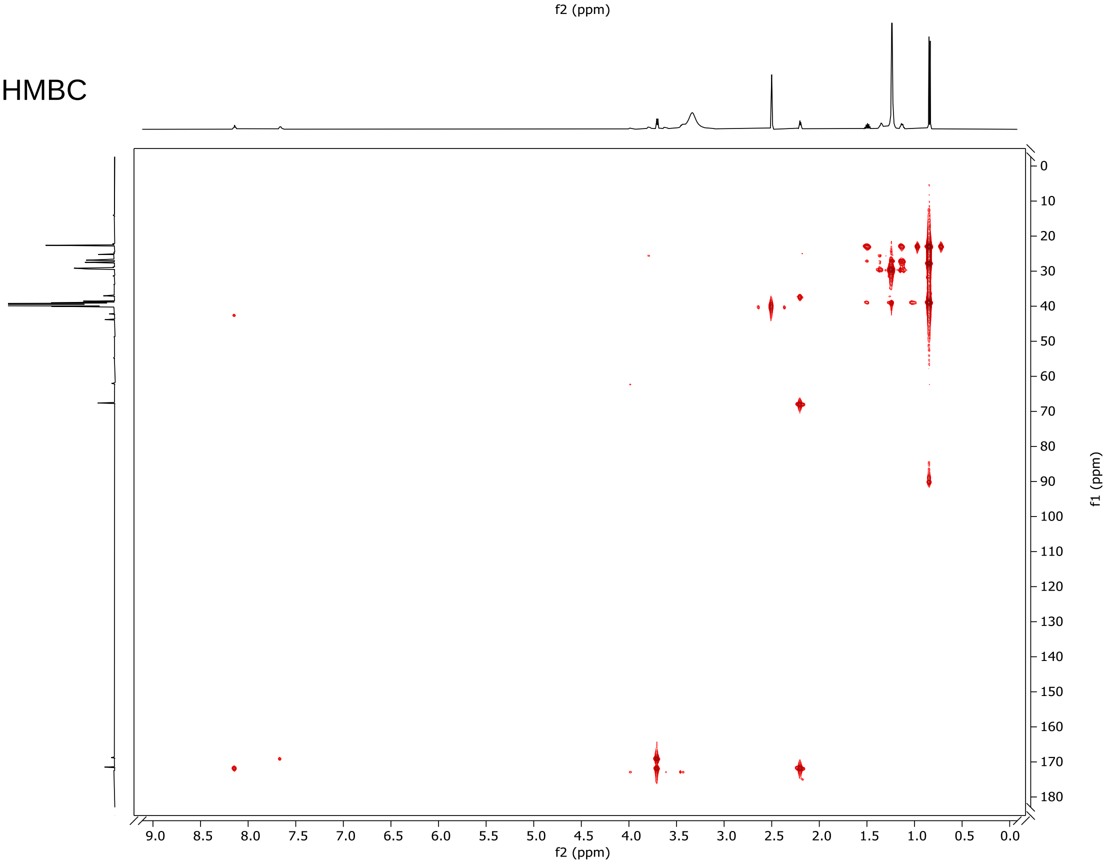

E gCOSY

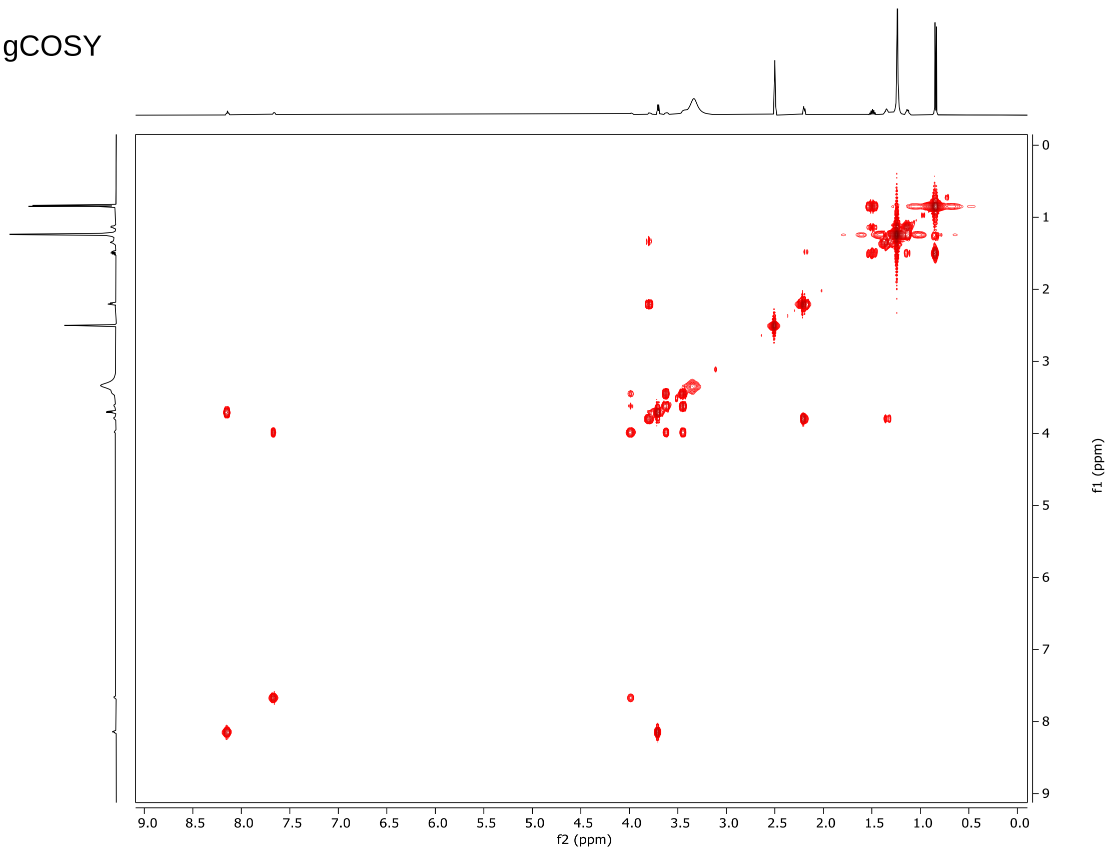

F NOESY

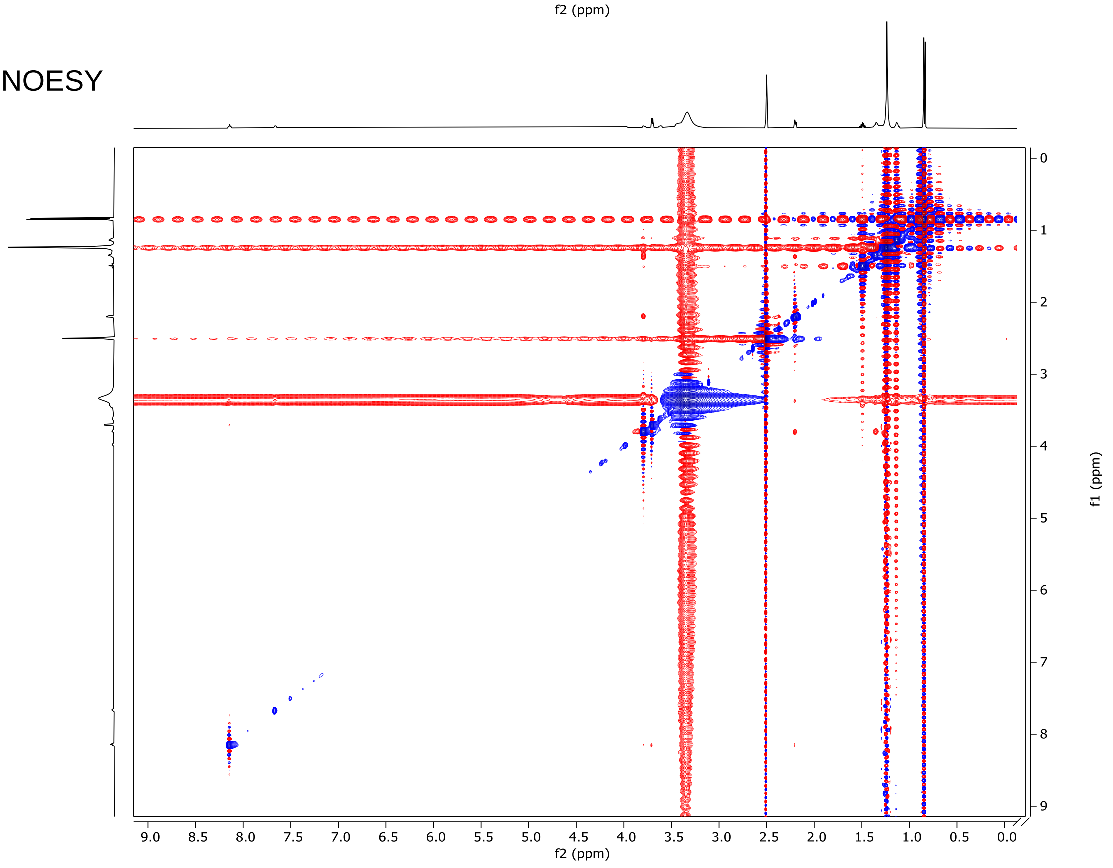

Figure S10. 1D and 2D NMR spectra of **12** (*m/z* 431.31) in DMSO-d_6.

A ^{1}H NMR

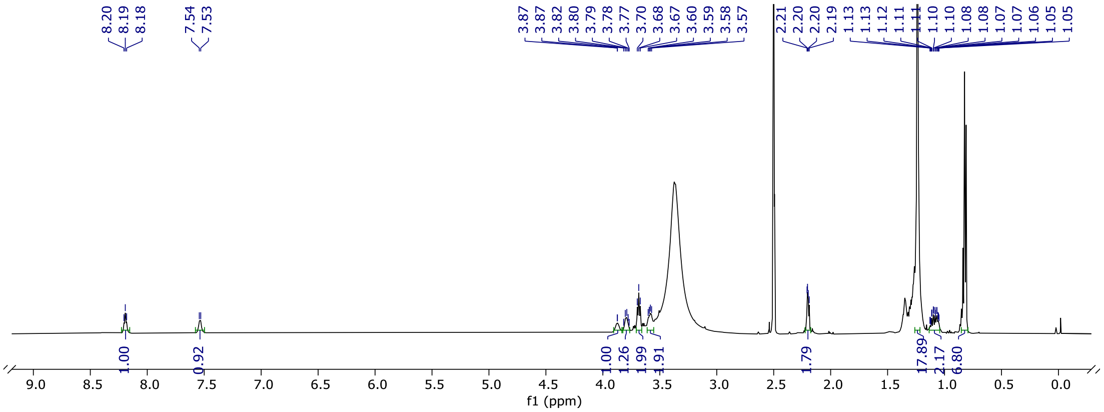

B ^{13}C NMR

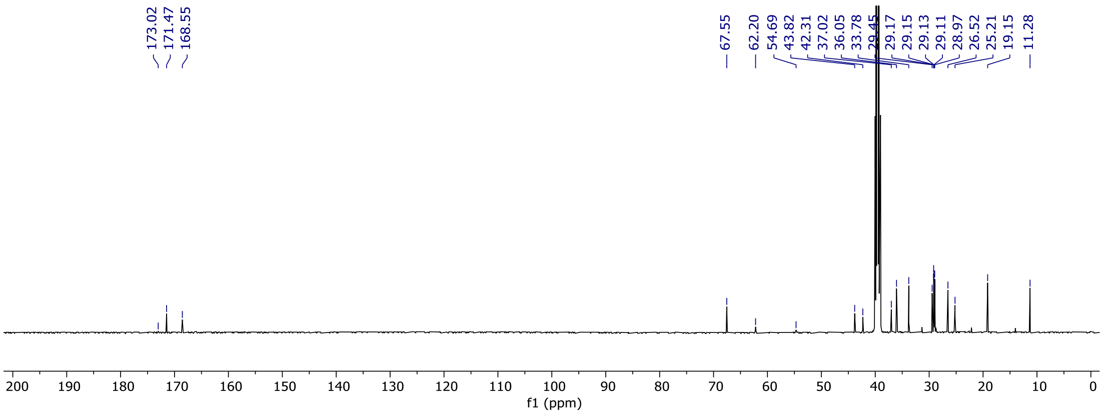

C HSQC

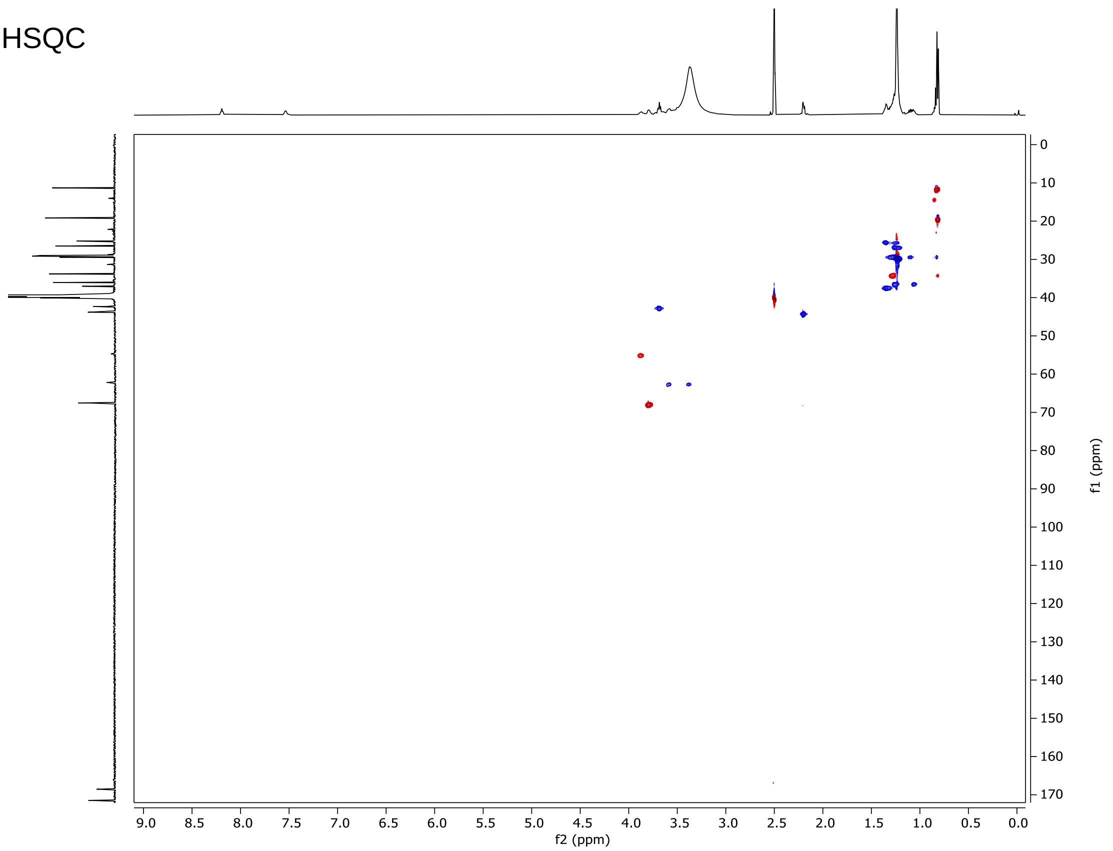

0
10
20
30
40
50
60
70
80
90
100
110
120
130
140
150
160
170
f1 (ppm)
9.0 8.5 8.0 7.5 7.0 6.5 6.0 5.5 5.0 4.5 4.0 3.5 3.0 2.5 2.0 1.5 1.0 0.5 0.0
f2 (ppm)

D HMBC

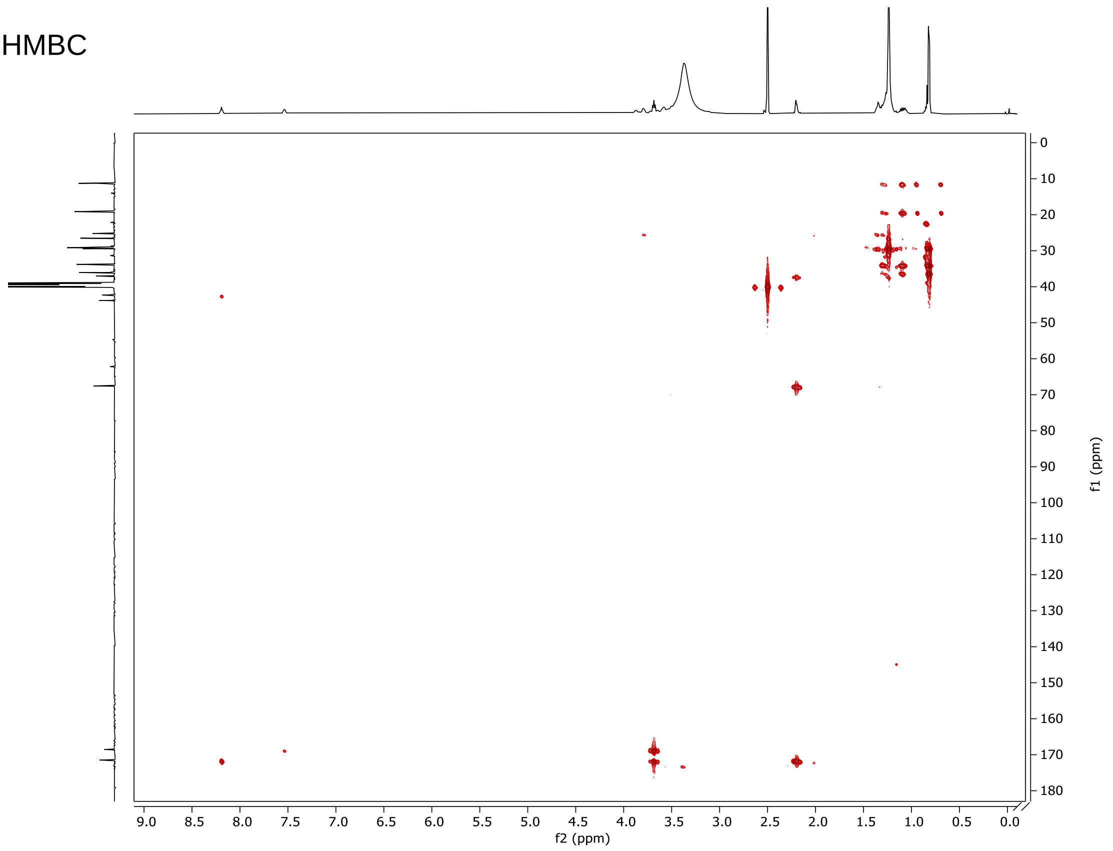

0
10
20
30
40
50
60
70
80
90
100
110
120
130
140
150
160
170
180
f1 (ppm)
9.0 8.5 8.0 7.5 7.0 6.5 6.0 5.5 5.0 4.5 4.0 3.5 3.0 2.5 2.0 1.5 1.0 0.5 0.0
f2 (ppm)

E gCOSY

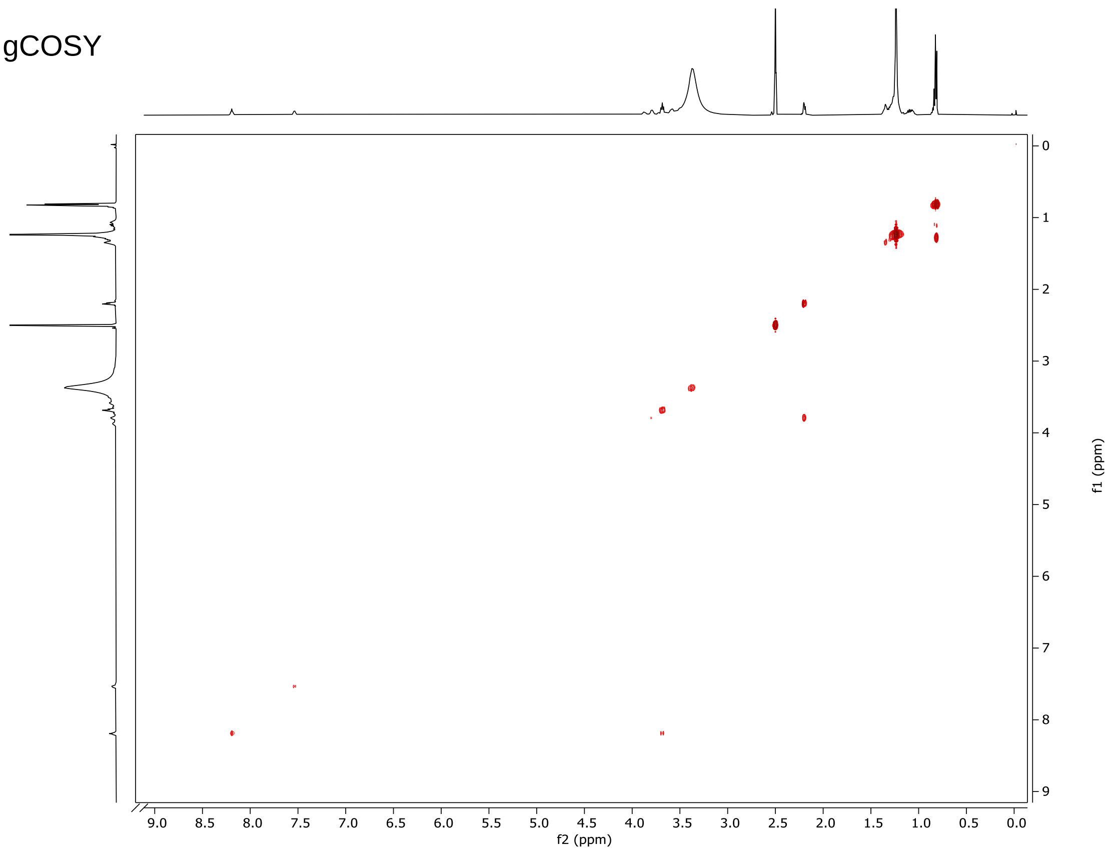

F NOESY

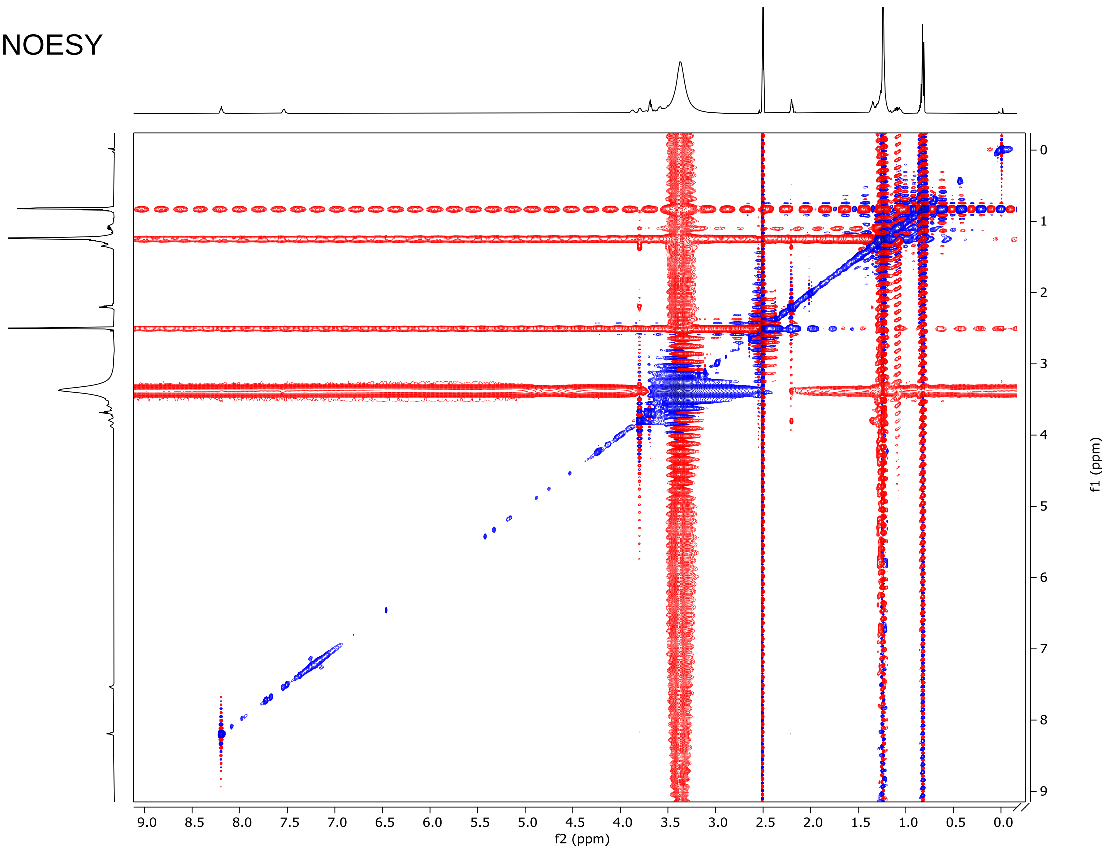

Figure S11. 1D and 2D NMR spectra of **14** (*m/z* 445.33) in DMSO-d_6.

A ^{1}H NMR

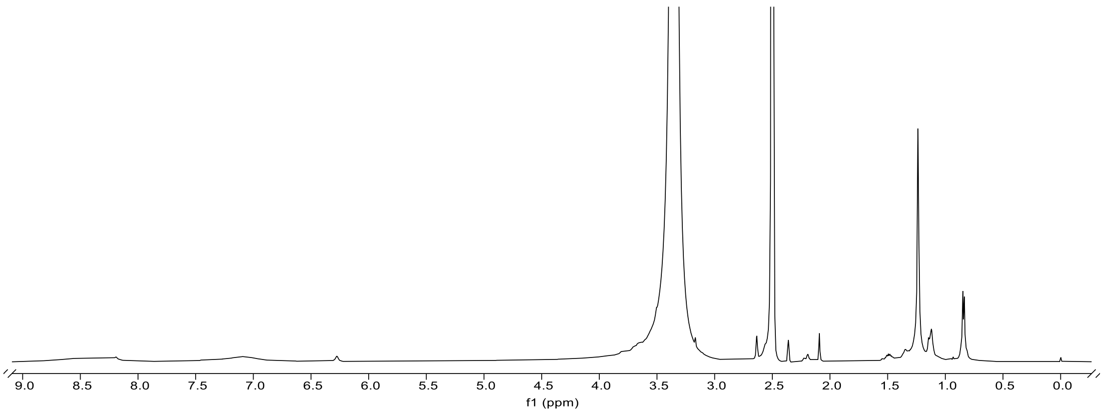

B HSQC

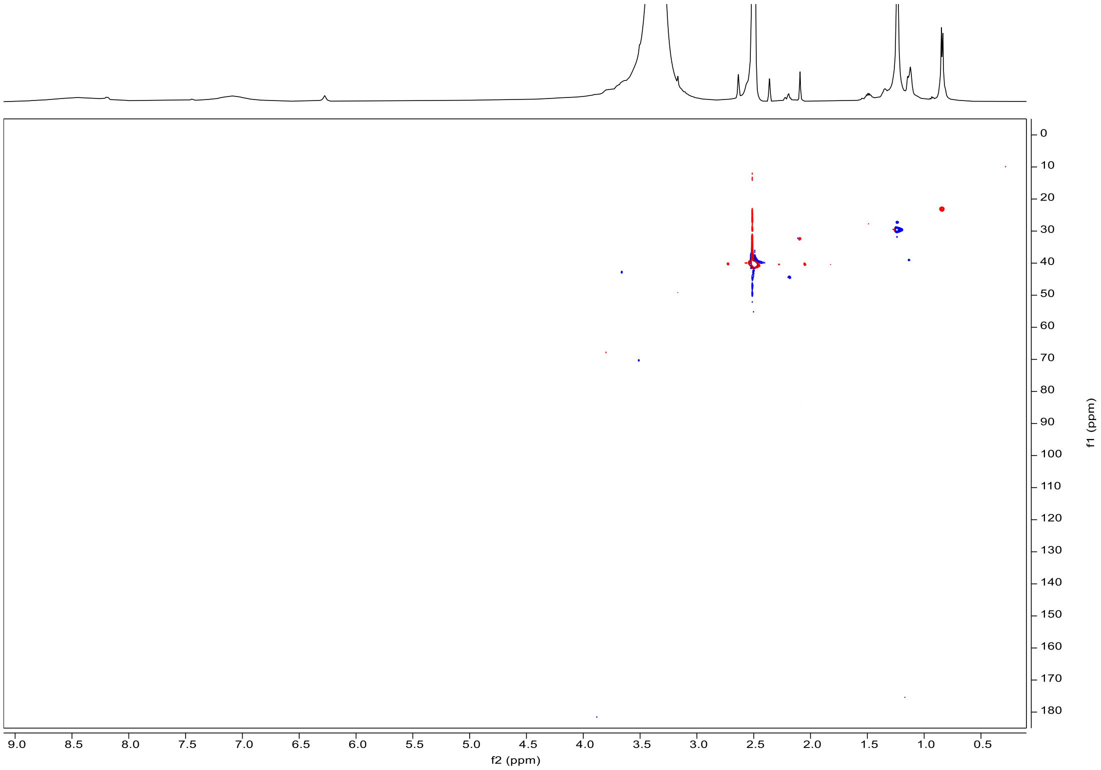

C HMBC

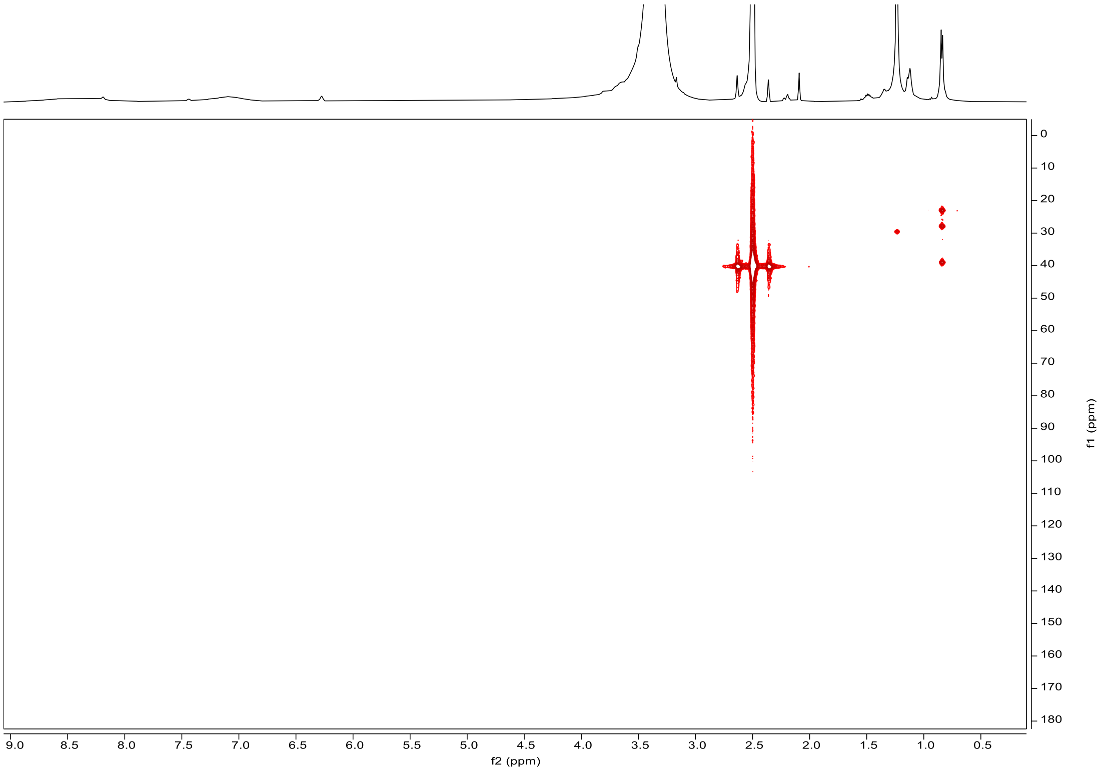
f1 (ppm)
f2 (ppm)
0
10
20
30
40
50
60
70
80
90
100
110
120
130
140
150
160
170
180
9.0
8.5
8.0
7.5
7.0
6.5
6.0
5.5
5.0
4.5
4.0
3.5
3.0
2.5
2.0
1.5
1.0
0.5

D COSY

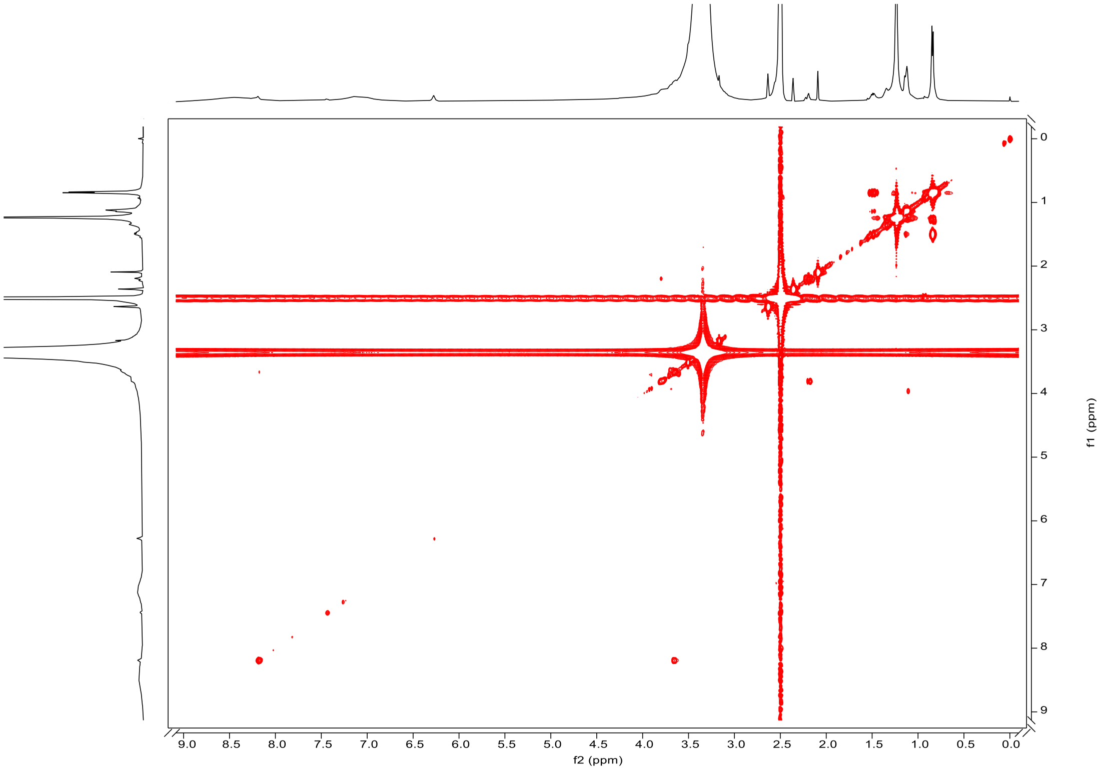
f1 (ppm)
f2 (ppm)
0
1
2
3
4
5
6
7
8
9
9.0
8.5
8.0
7.5
7.0
6.5
6.0
5.5
5.0
4.5
4.0
3.5
3.0
2.5
2.0
1.5
1.0
0.5
0.0

Figure S12. A mass shift in dipeptide lipids (**10**–**12**) by feeding of isotope-labeled branched chain amino acids (BCAAs) to *B. dorei*. (A–C) Incorporation of the isotopologue with *m/z* shifts of +7 from **10** (A) and +9 from **11** (B) and **12** (C), suggesting L-valine, L-leucine, and L-isoleucine are precursors for these metabolites, respectively. (D–F) HR-MS/MS data of the labeled species (orange box). Consistent fragment patterns were observed from the labeled metabolites compared to the unlabeled ones. The only deviations in fragment masses (+7 or +9 Da shift) are ones containing the acyl chain.

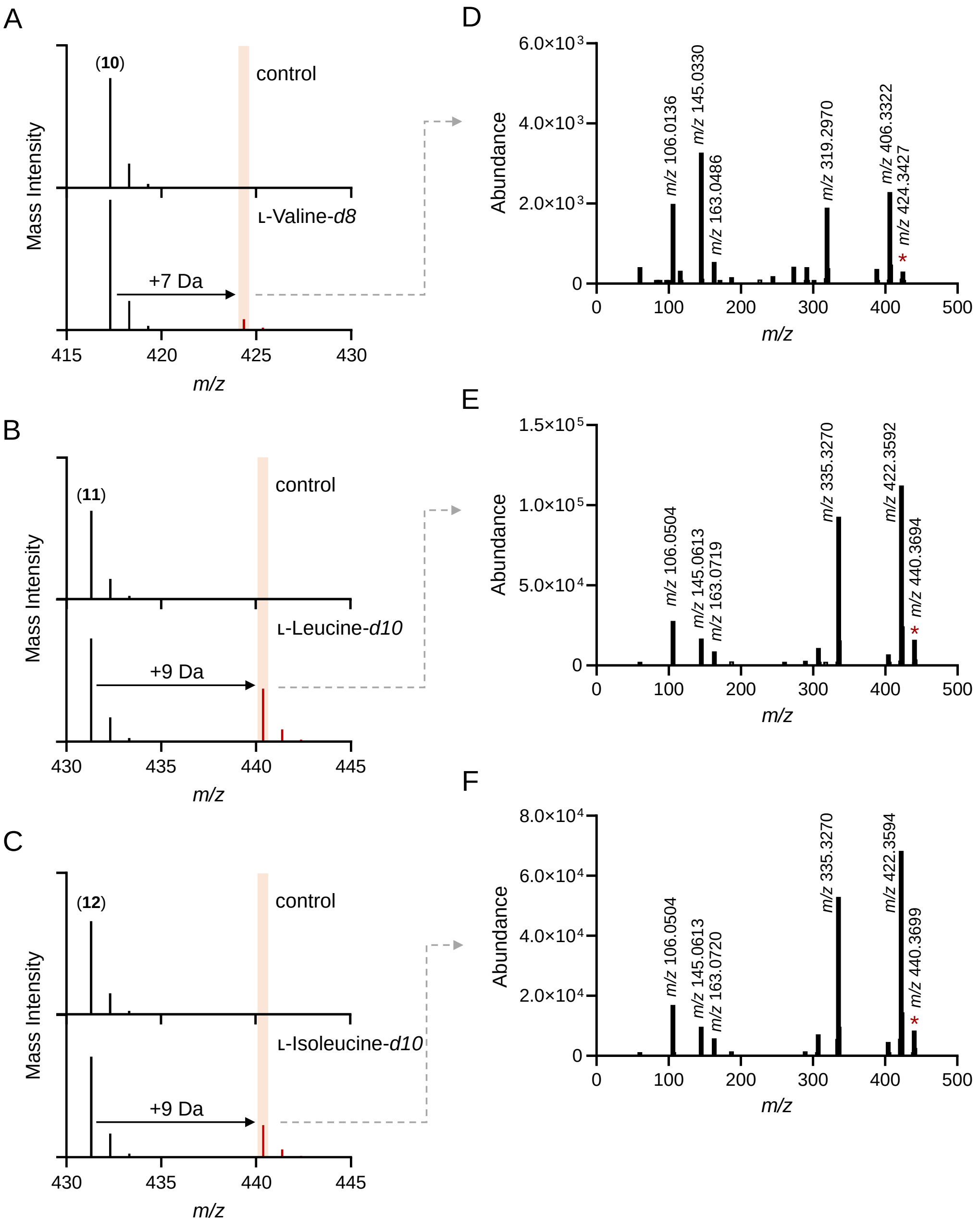

Figure S13. HR-MS/MS data for *N*-acylated adenosine variants (**16** and **17**). Collision-induced ion loss of 136 and 85 were observed from compounds **16** (A) and **17** (B).

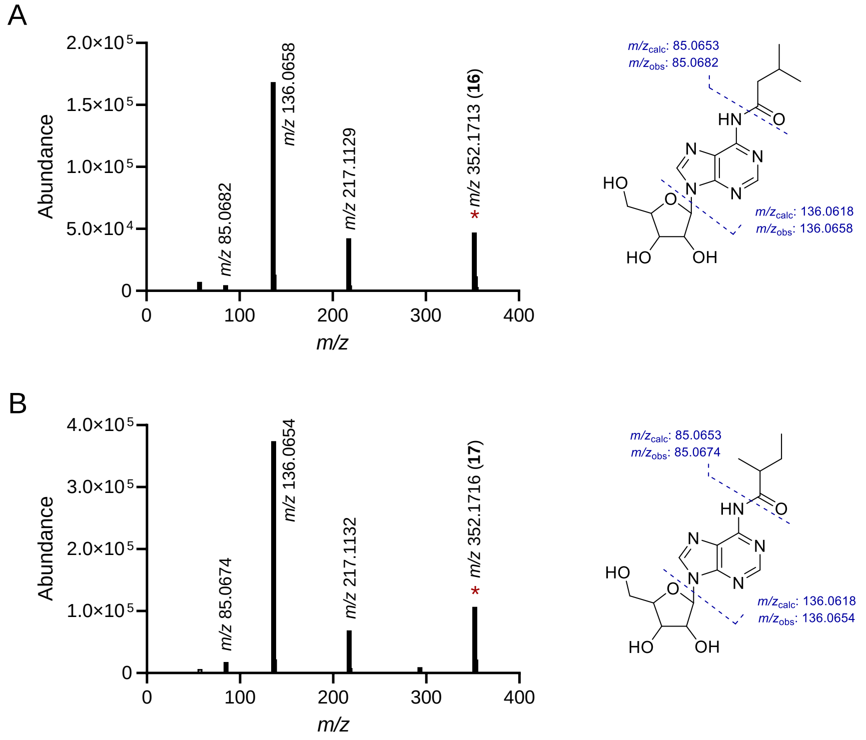

Figure S14. 1D and 2D NMR spectra of **16** (*m/z* 352.16) in DMSO-d_6.

A ^{1}H NMR

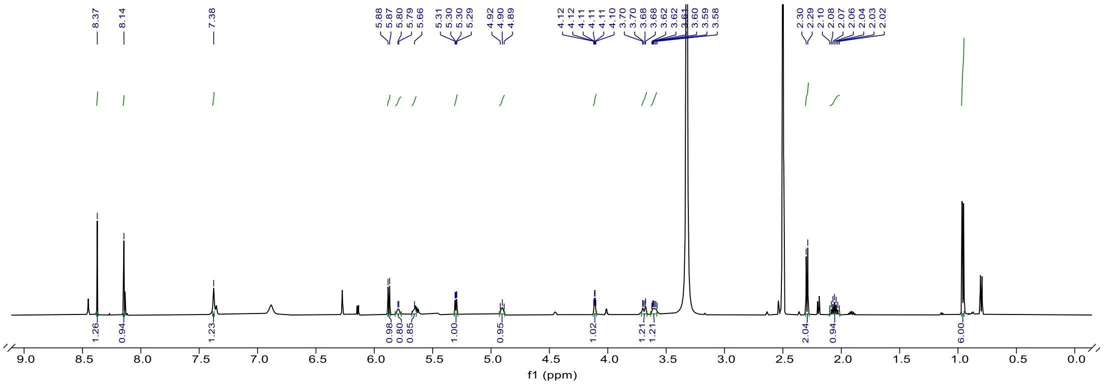

B HSQC

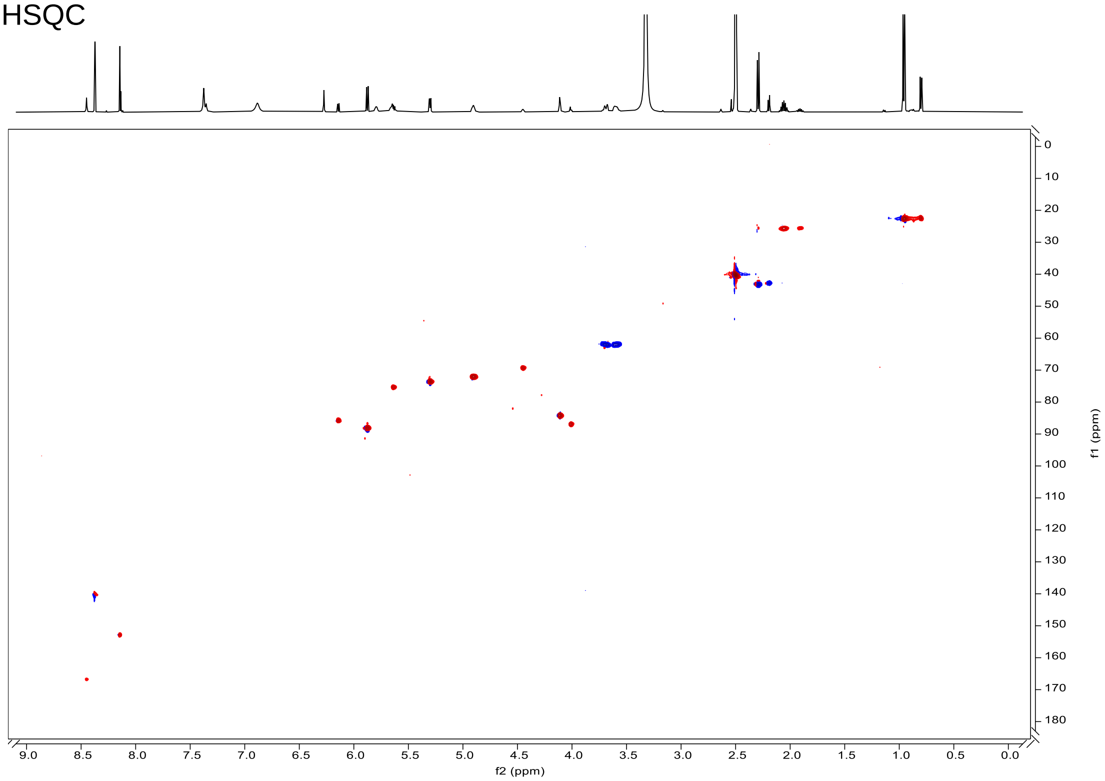

C HMBC

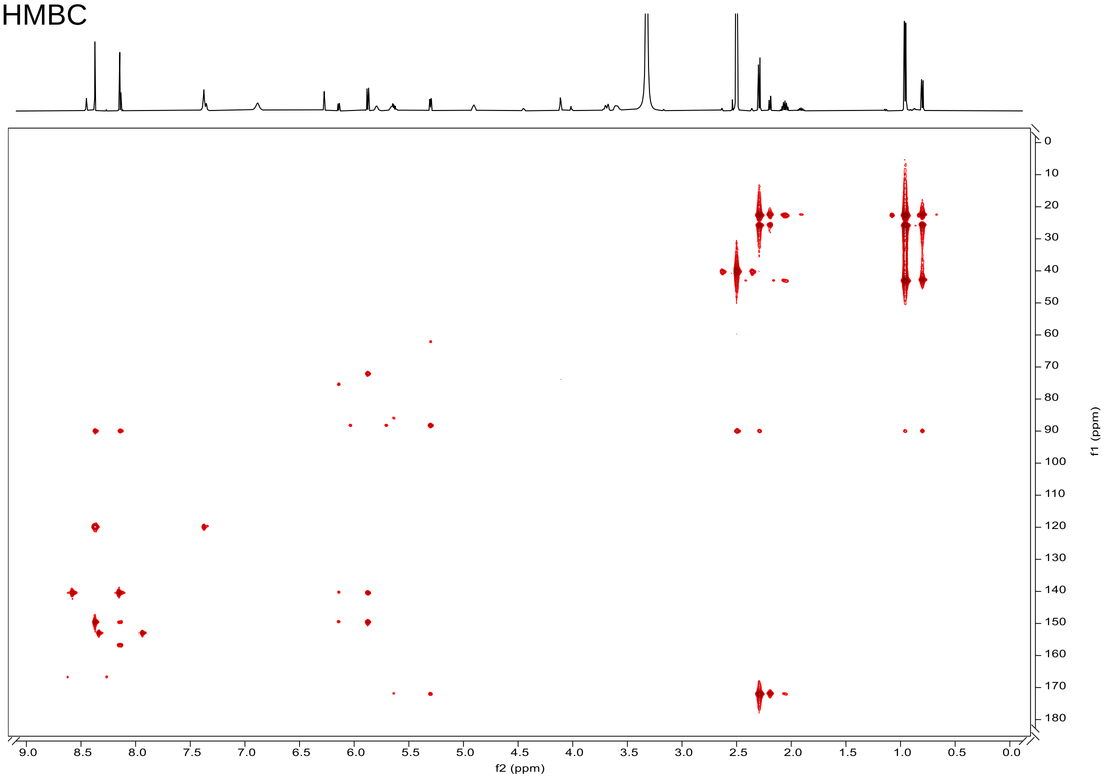

D gCOSY

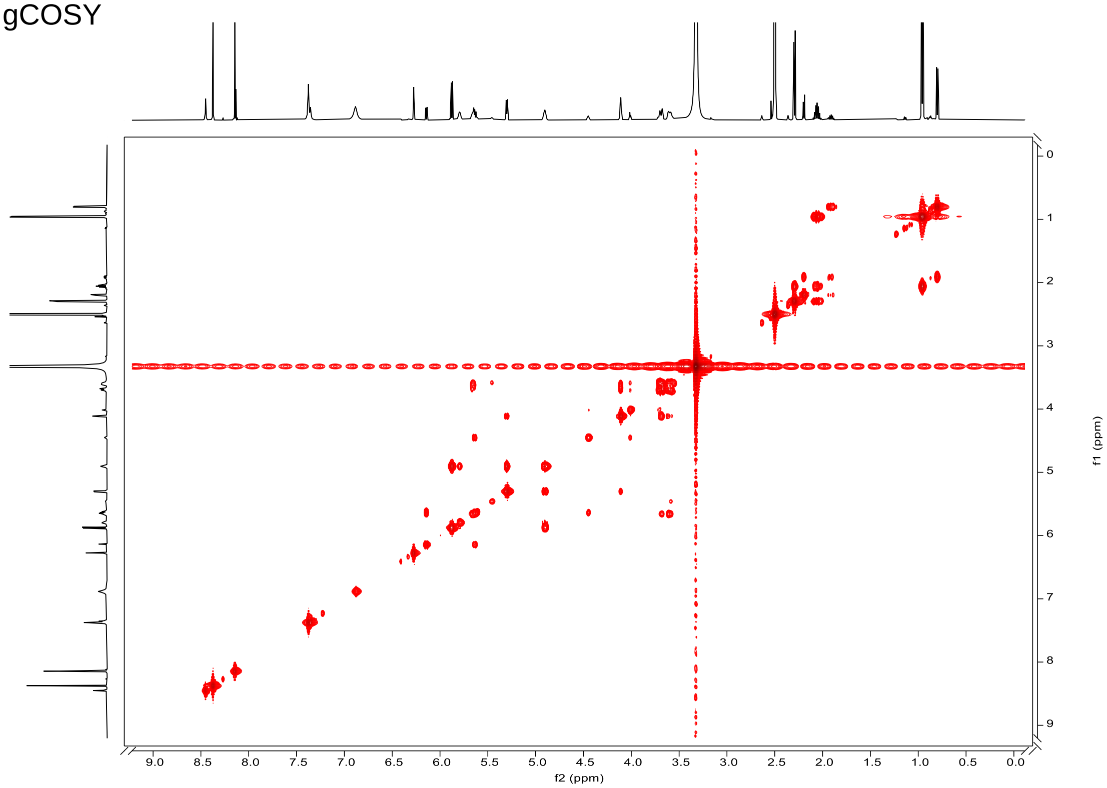

E NOESY

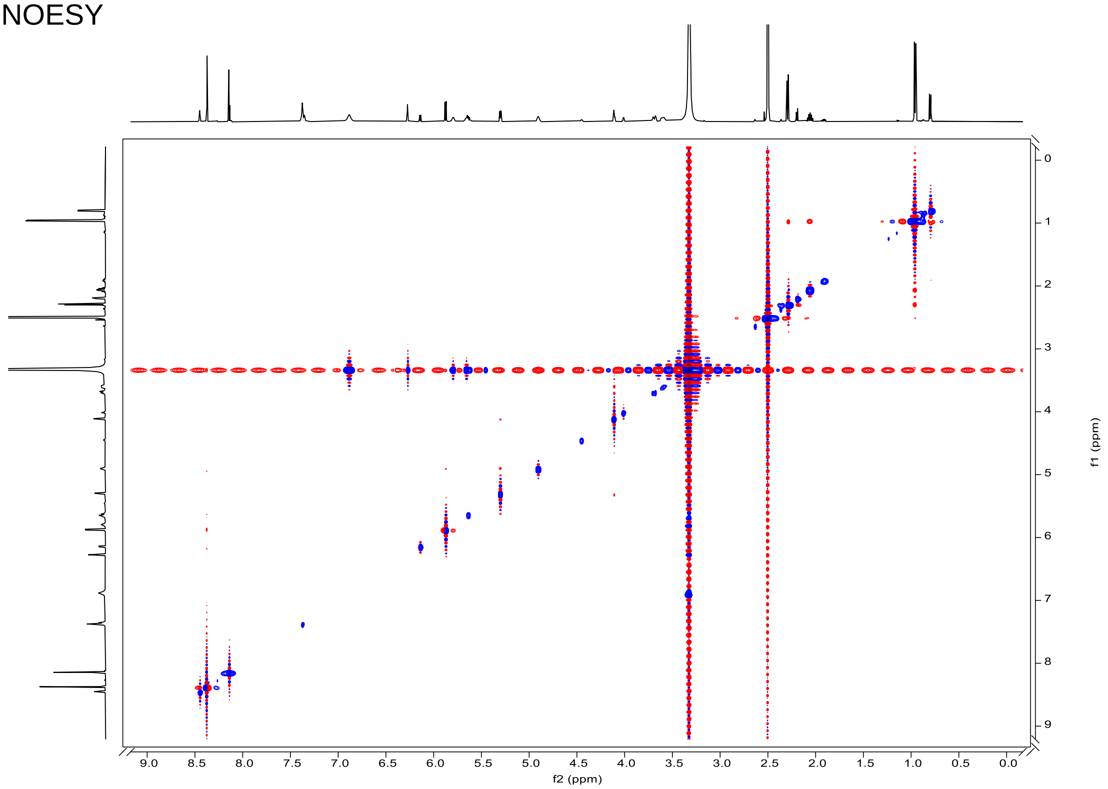

Figure S15. 1D and 2D NMR spectra of **17** (*m/z* 352.16) in DMSO-d_6.

A ^{1}H NMR

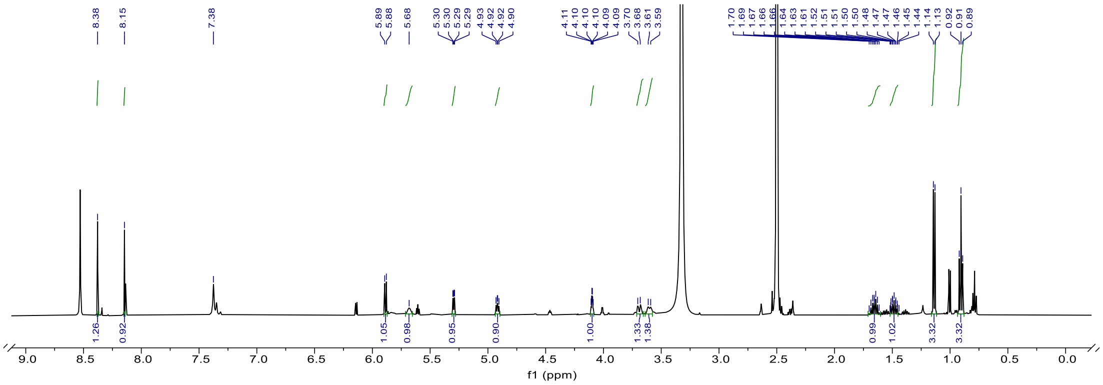

B HSQC

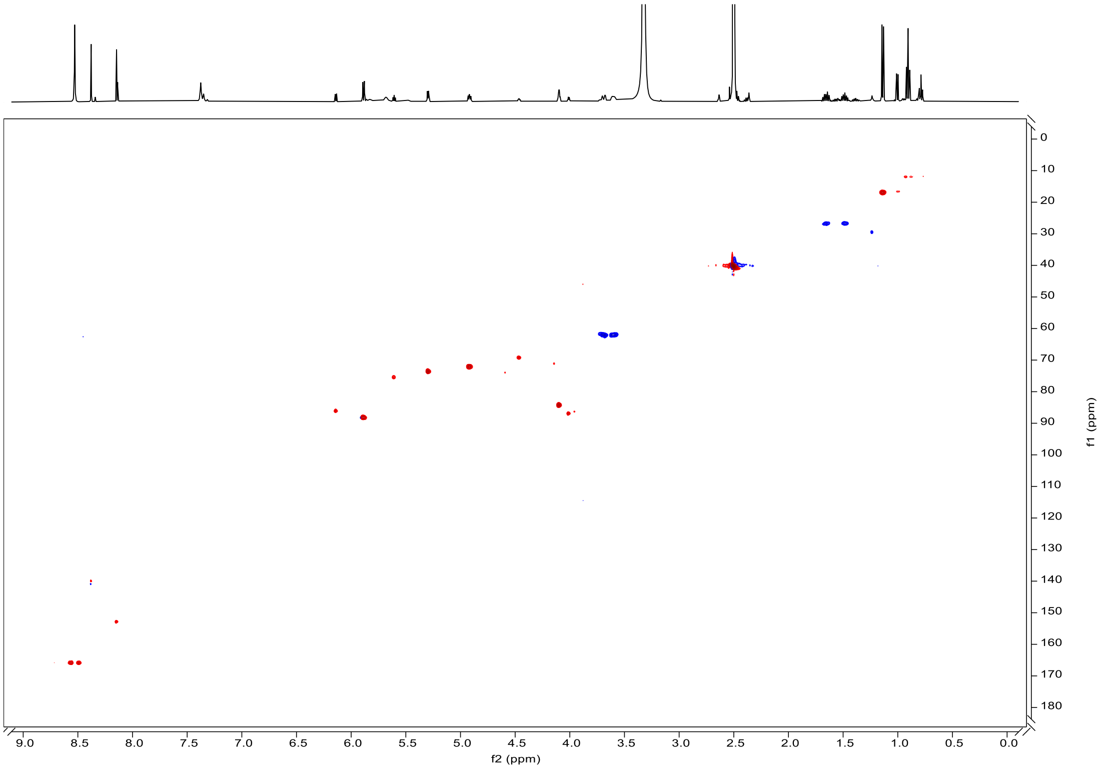

C HMBC

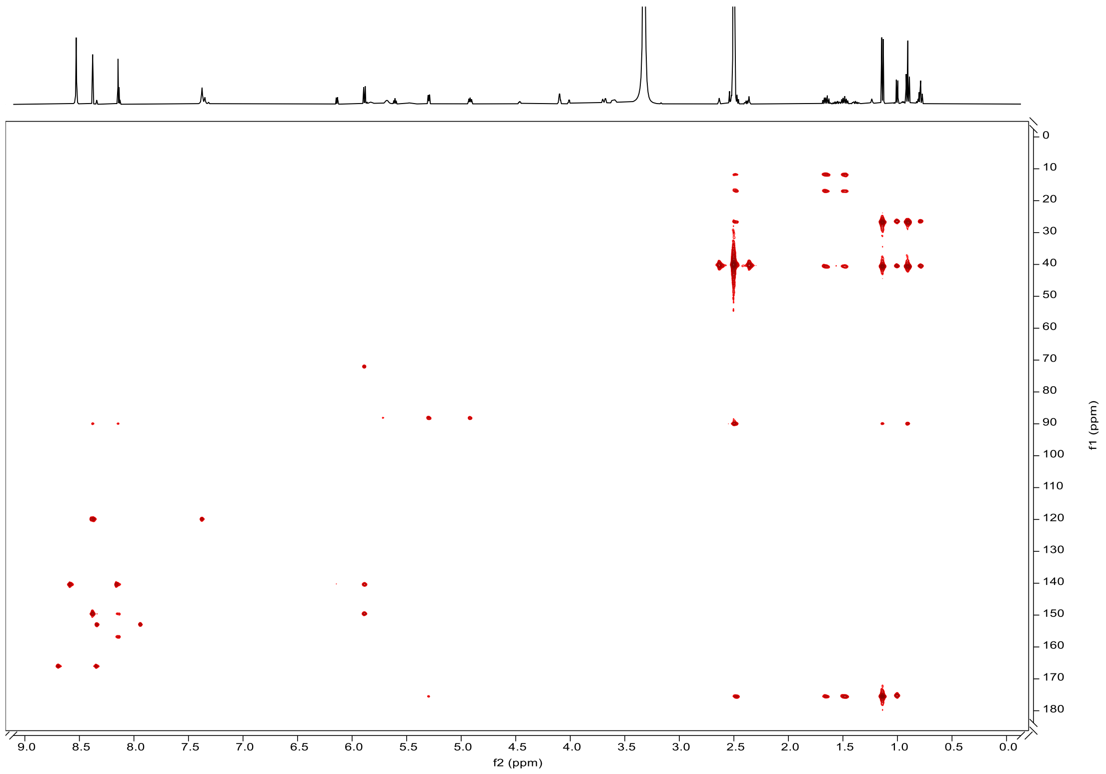

D gCOSY

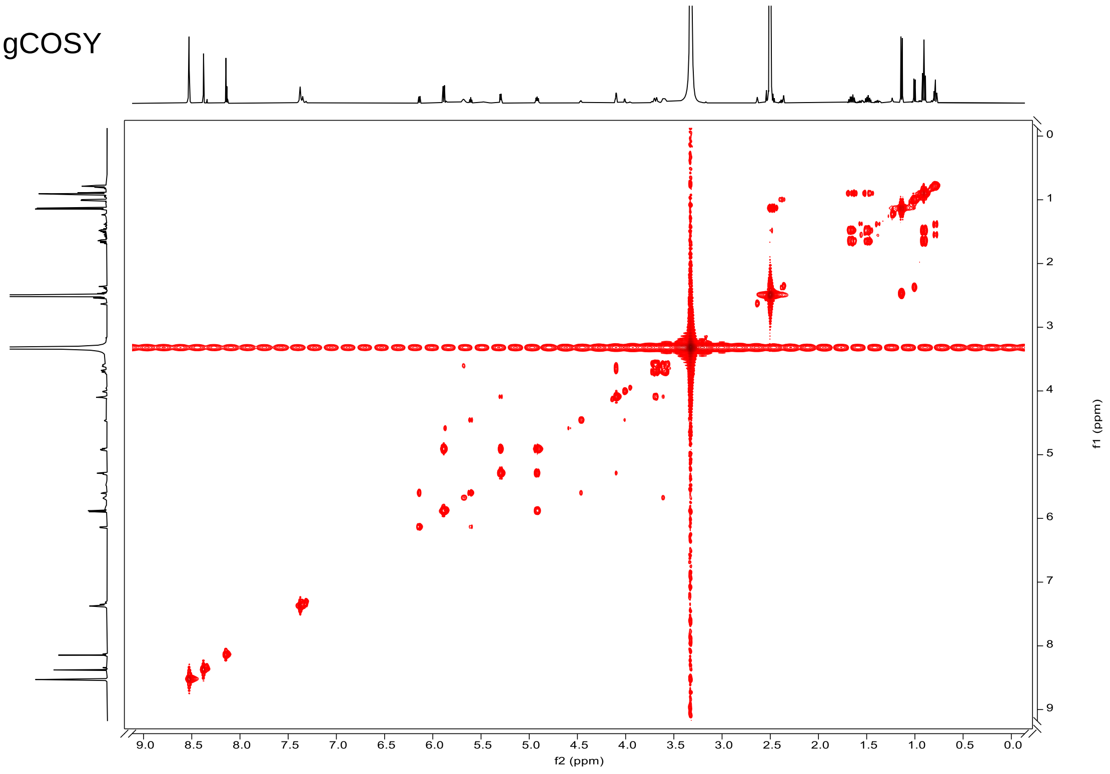

E NOESY

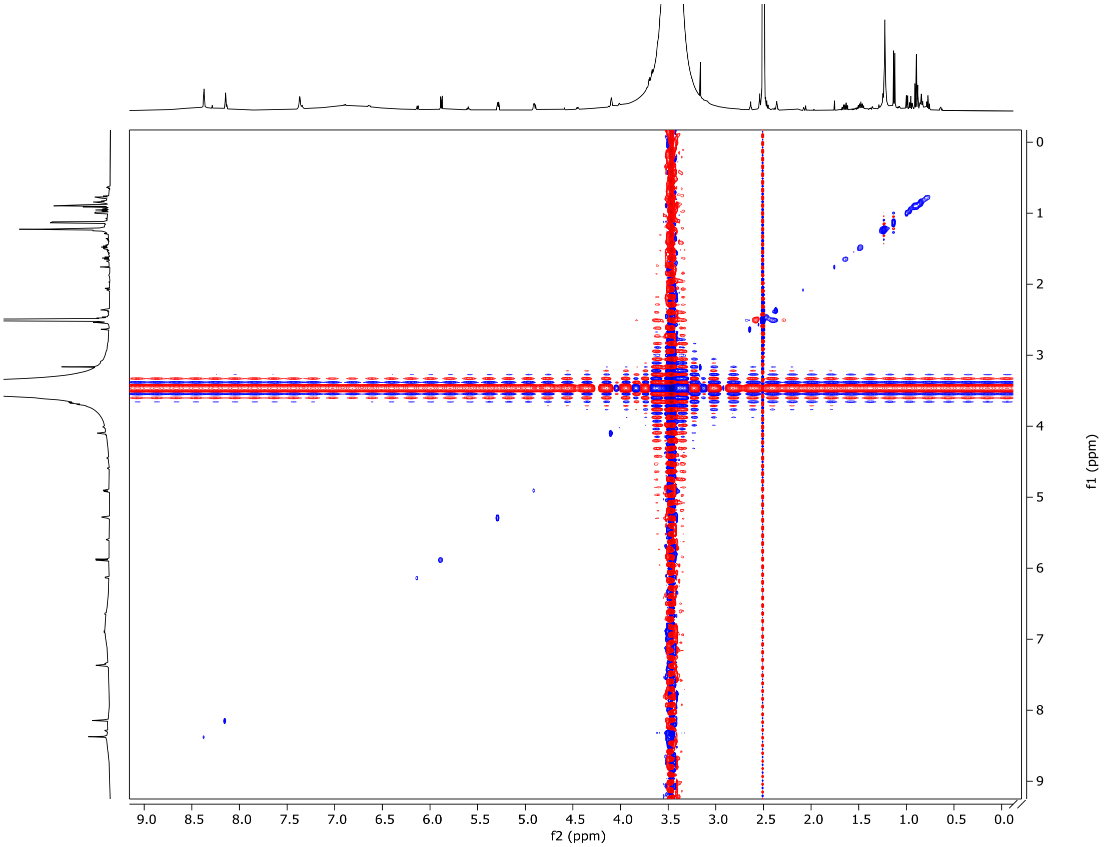

0
1
2
3
4
5
6
7
8
9
f1 (ppm)
9.0
8.5
8.0
7.5
7.0
6.5
6.0
5.5
5.0
4.5
4.0
3.5
3.0
2.5
2.0
1.5
1.0
0.5
0.0
f2 (ppm)

Figure S16. ^{1}H NMR spectrum of **16** in DMSO-d_6, with peak picking highlighting signals corresponding to the minor conformer.

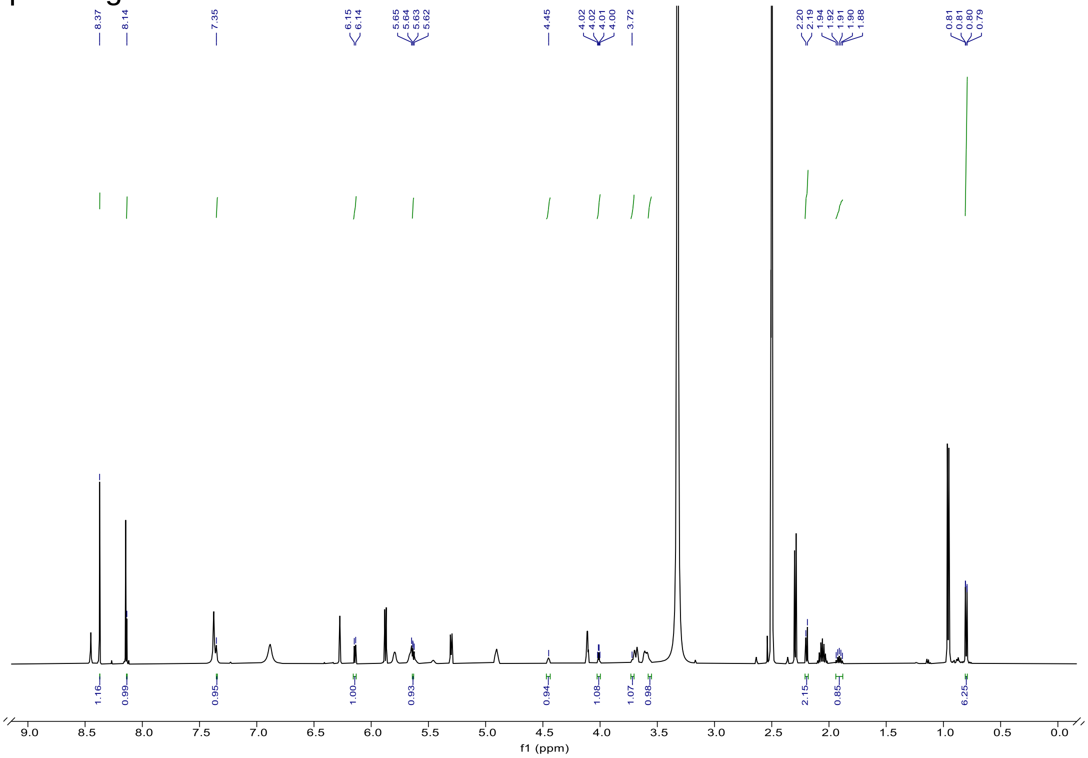

Figure S17. ^{1}H NMR spectrum of **17** in DMSO-d_6, with peak picking highlighting signals corresponding to the minor conformer.

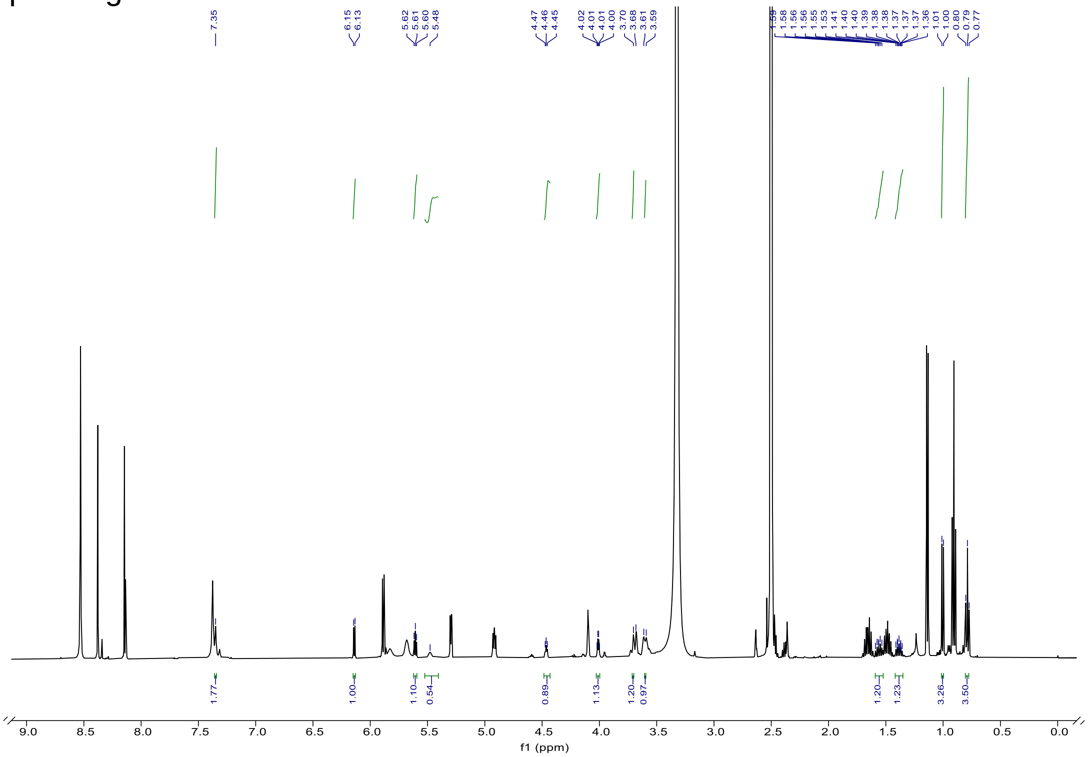

Figure S18. Variable-temperature (VT) ^{1}H NMR spectra of **16** from 298 K to 388 K at 500 MHz in DMSO-d_6.

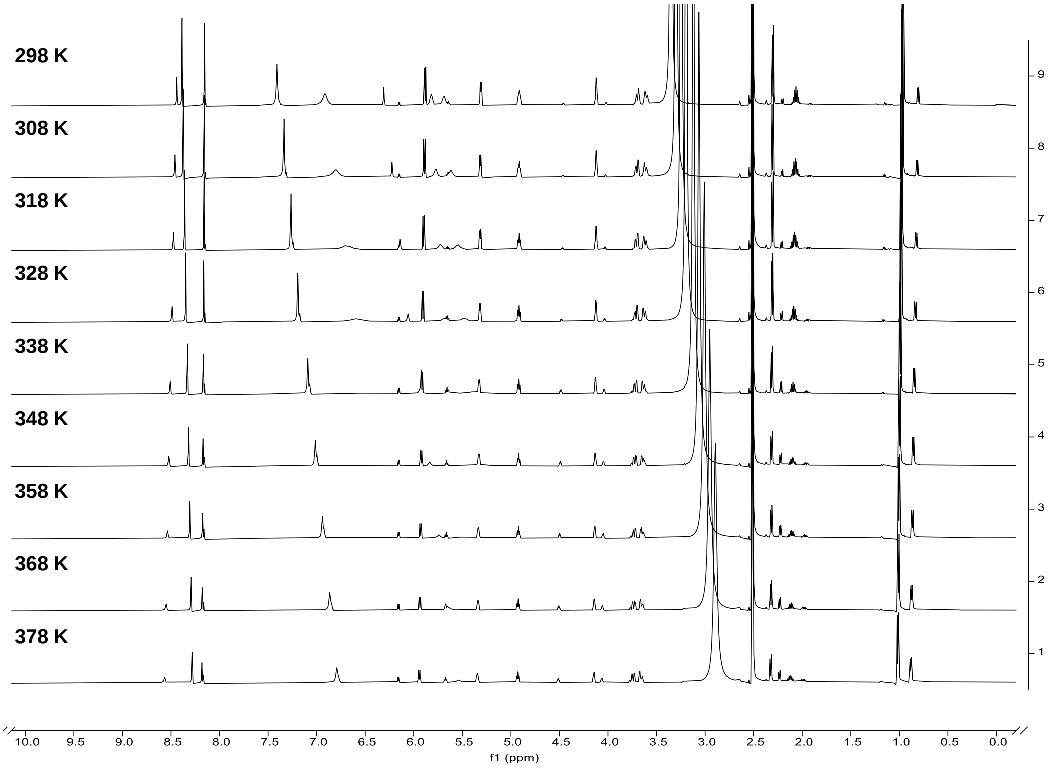

Figure S19. Overlay of VT ^{1}H NMR spectra aligned to the H-4'' (δ_H 0.95 ppm) of **16**.

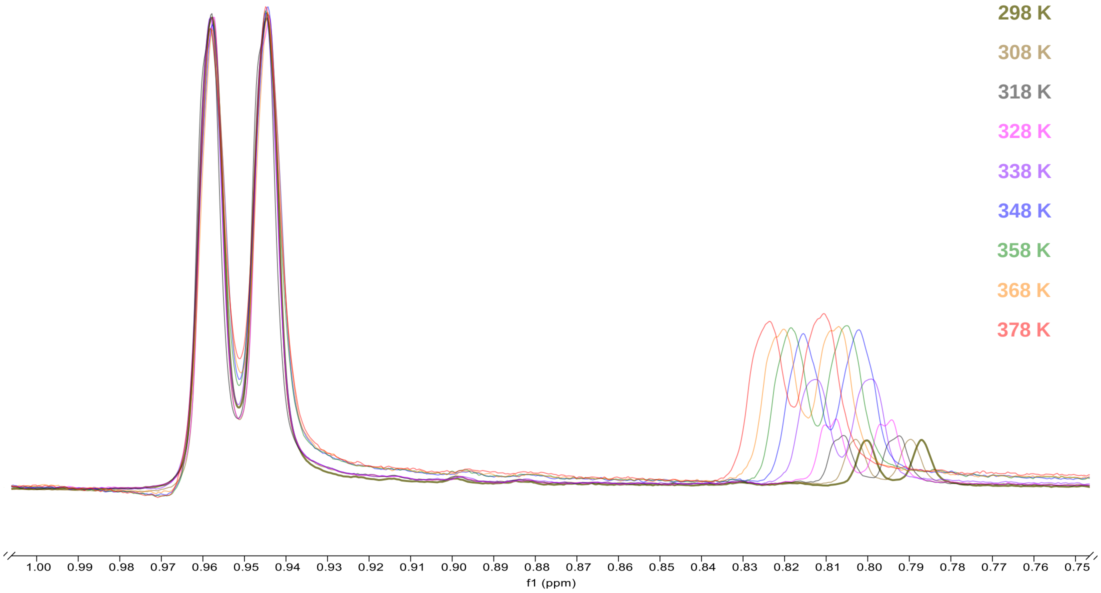

Figure S20. Variable-temperature (VT) ^{1}H NMR spectra of **17** from 298 K to 378 K at 500 MHz in DMSO-d_6.

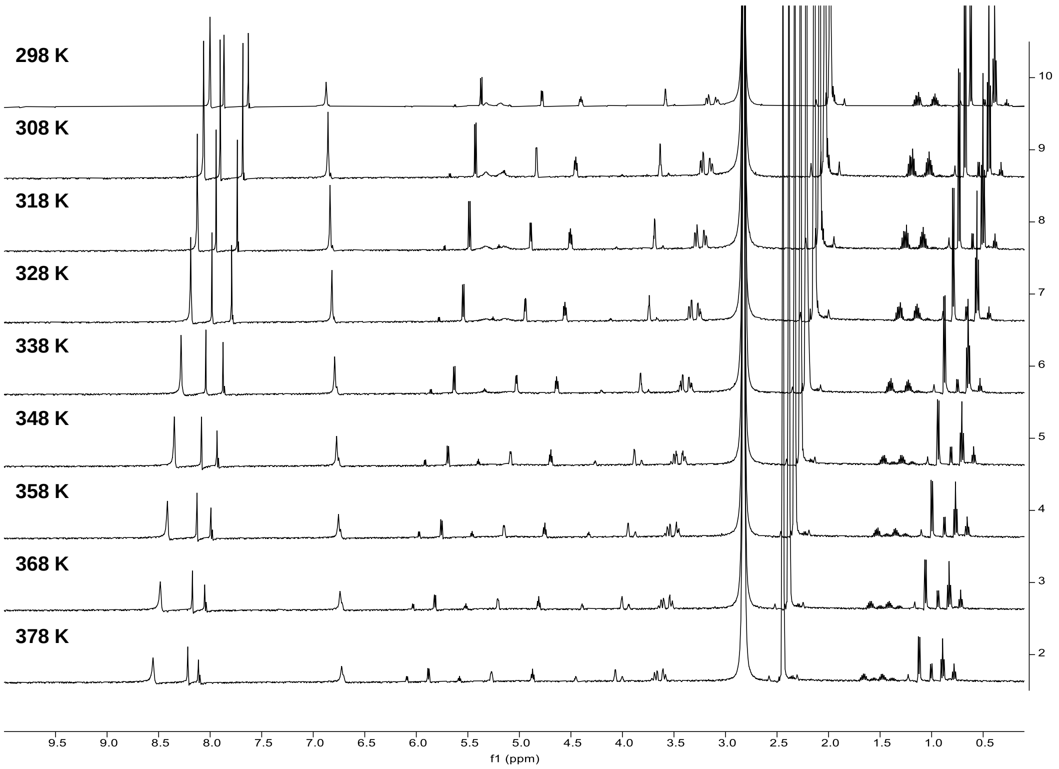

Figure S21. Overlay of VT 1H NMR spectra aligned to the H-5'' (δ_H 1.55 ppm) of **17**.

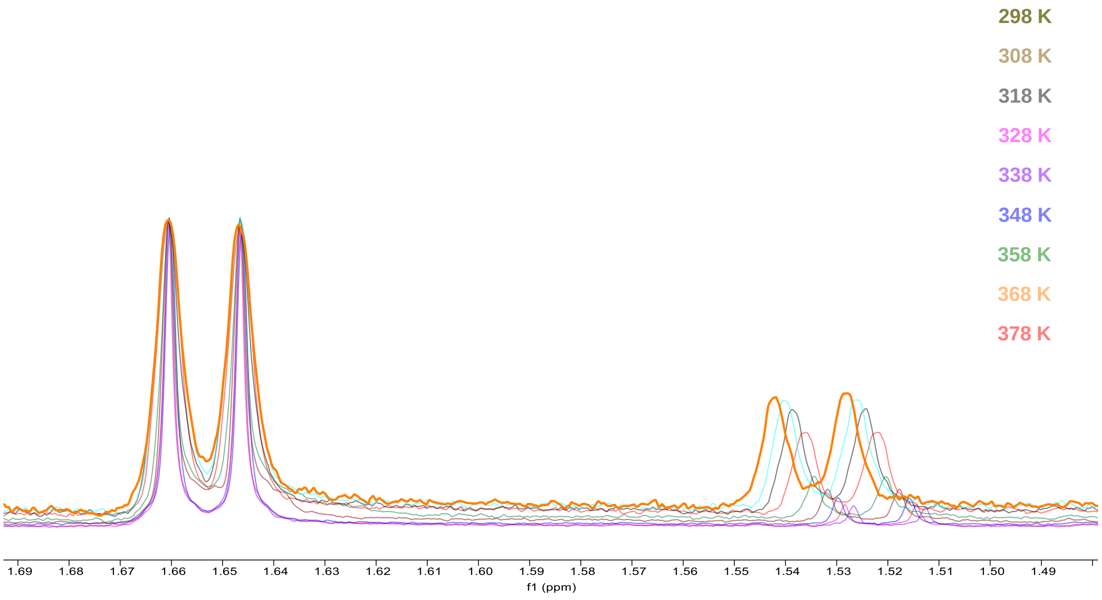

Figure S22. A mass shift in *N*-acylated adenosine variants by feeding of isotope-labeled branched chain amino acids (BCAAs) to *B. dorei*. (A–H) Incorporation of the isotopologue with *m/z* shifts of +9 from **16/*conf*-16** (A–B) and **17/*conf*-17** (C–D), suggesting L-leucine and L-isoleucine are precursors for these metabolites, respectively. (E–H) HR-MS/MS data of the labeled species (orange box). Consistent fragment patterns were observed from the labeled metabolites compared to the unlabeled ones. The only deviations in fragment masses (+9 Da shift) are ones containing the acyl chain.

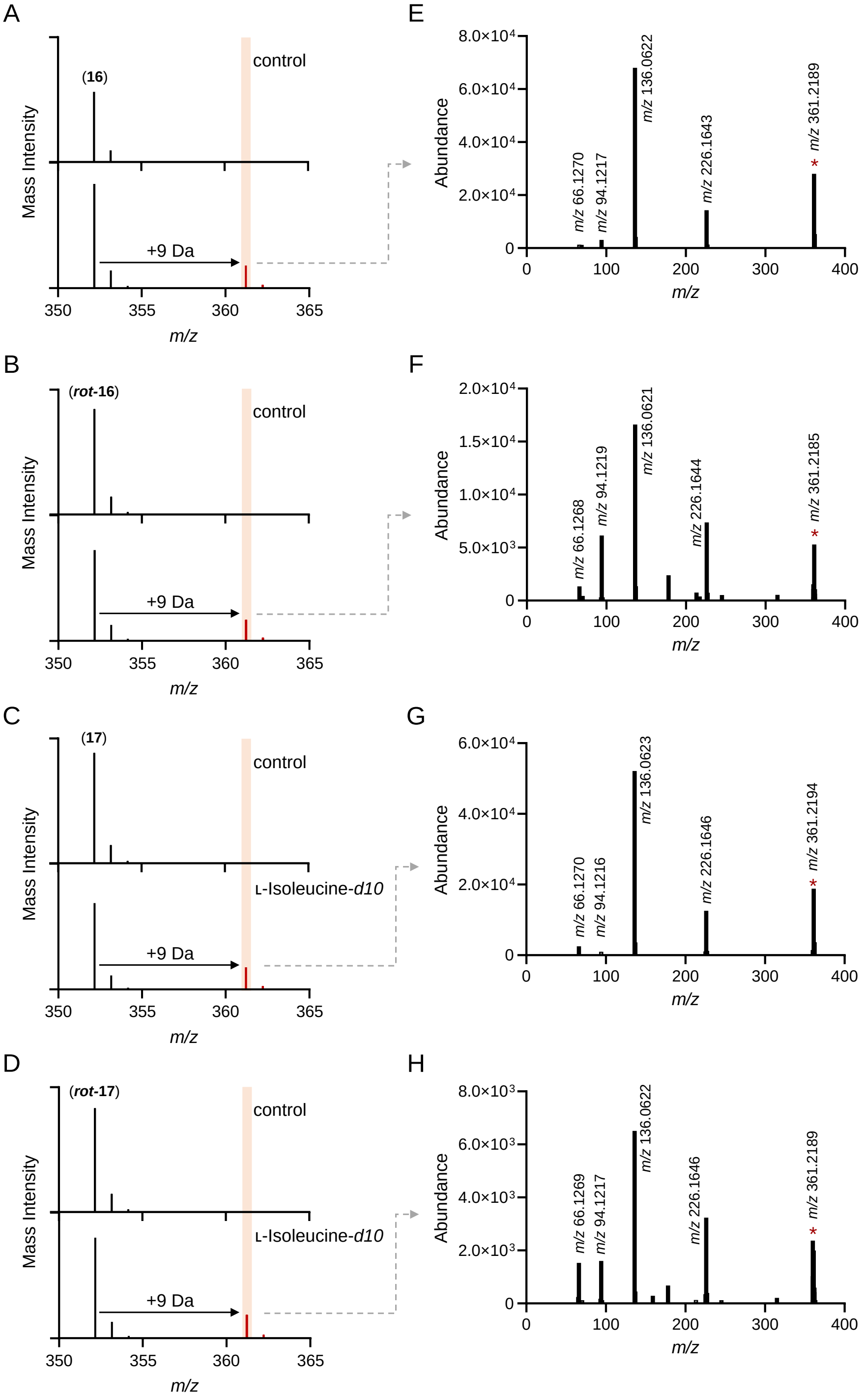

Figure S23. Cytotoxicity of doreamides A–C (**10–12**) and 6-*N*-acyladenosines (**16** and **17**) against RAW 264.7 macrophages after 24 h exposure.

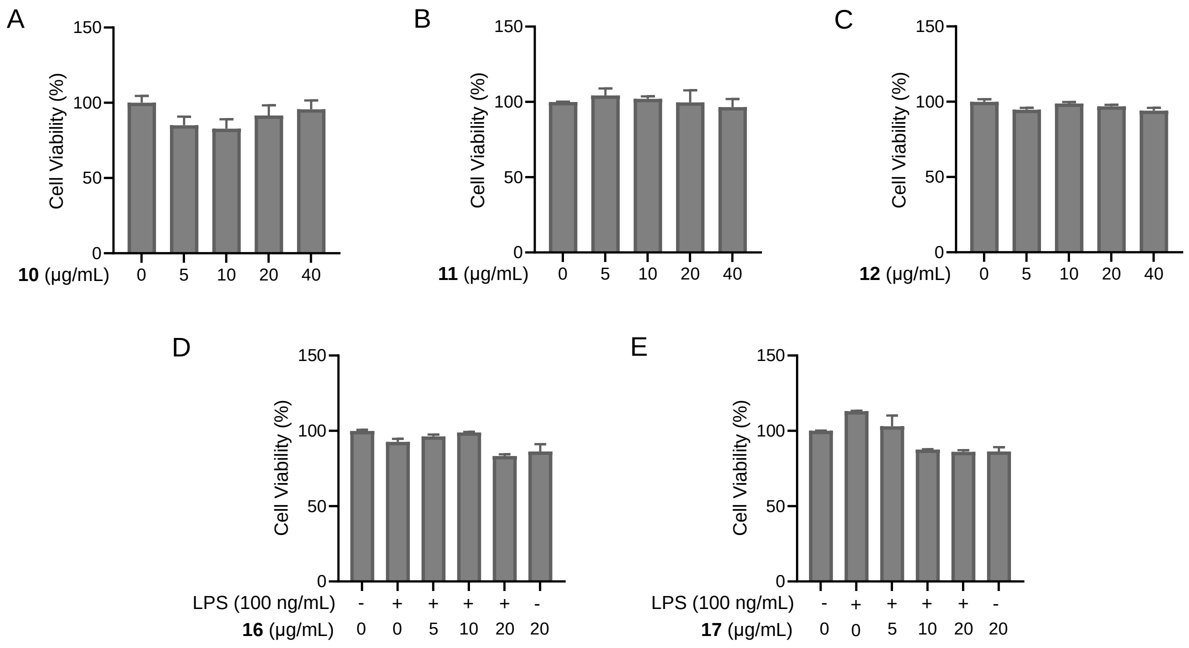

Figure S24. Thin-layer chromatography (TLC) analysis demonstrating absence of LPS in compounds **14** and **16**. (A) TLC analysis of LPS under different solvent conditions. Plates were eluted with 30% MeOH/DCM and visualized with *p*-anisaldehyde stain. LPS did not partition into the ethyl acetate (EA) organic layer used in our extraction procedure, instead remaining in the aqueous phase. Images are shown under UV (254 nm, left) and visible light (right). Co: co-injection of LPS, LPS-EA, and LPS-water. (B) TLC comparison of **14** and **16** with LPS. The LPS spot was absent in these purified compounds, indicating LPS is below the limit of detection. Co: co-injection of **14**, **16**, and LPS.

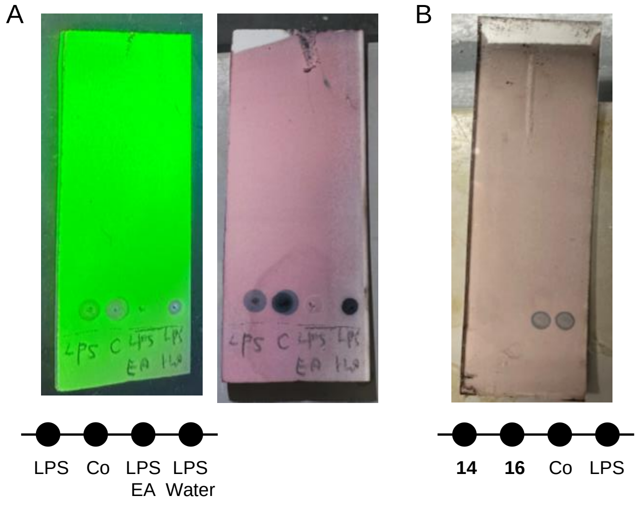

Figure S25. Limulus amebocyte lysate (LAL, Pierce^{TM}) assay-based confirmation of the absence of detectable LPS in compounds **16** and **17**. The kit was used according to manufacturer's instructions without modifications. (A) Standard curve generated for LPS quantification. (B) Quantified LPS levels in purified compounds after blank subtraction, showing values below the detectable range and confirming the absence of LPS contamination. Considering that 1 ng/mL LPS corresponds to approximately 10 endotoxin units (EU)/mL, the data indicate that the level of LPS in our purified materials is negligible (formally ~1 pg/mL). The concentration typically used for the positive control is 10–100 ng/mL. Thus, the amount of LPS, if any, is 10^4–10^5-fold lower in our samples.

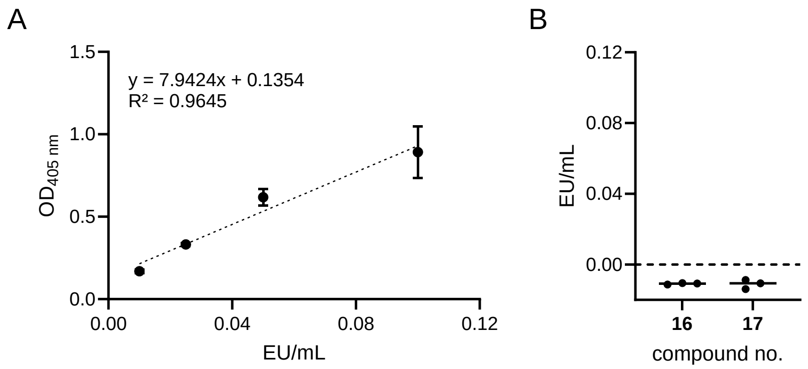

Figure S26. qRT-PCR of the genes in the doreamide BGC (*glsAB*), the branched chain amino acid transferase gene (*bcat*), and the *rpoB* housekeeping gene upon exposure to DMC (**1**) at 4 μM in comparison to the vehicle control. Data represent means ± S.E.M. (n=3). Significance was determined by multiple unpaired t-tests where * denotes a difference between vehicle control and indicated gene at $p<0.05$.

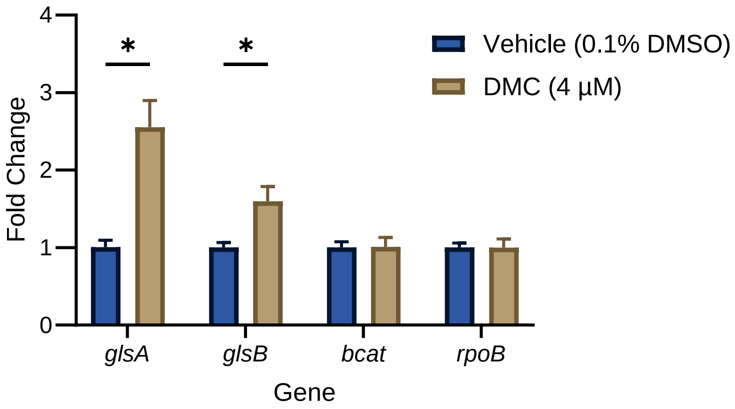

Figure S27. Extracted ion chromatograms (EICs, [M+H]$^+$) of GLs produced in *E. coli* BL21(DE3) harboring vectors expressing (A) *glsB* and *glsA,* (B) *glsB*, (C) *glsA*, or (D) empty vectors control. Each culture was treated with 500 µM IPTG.

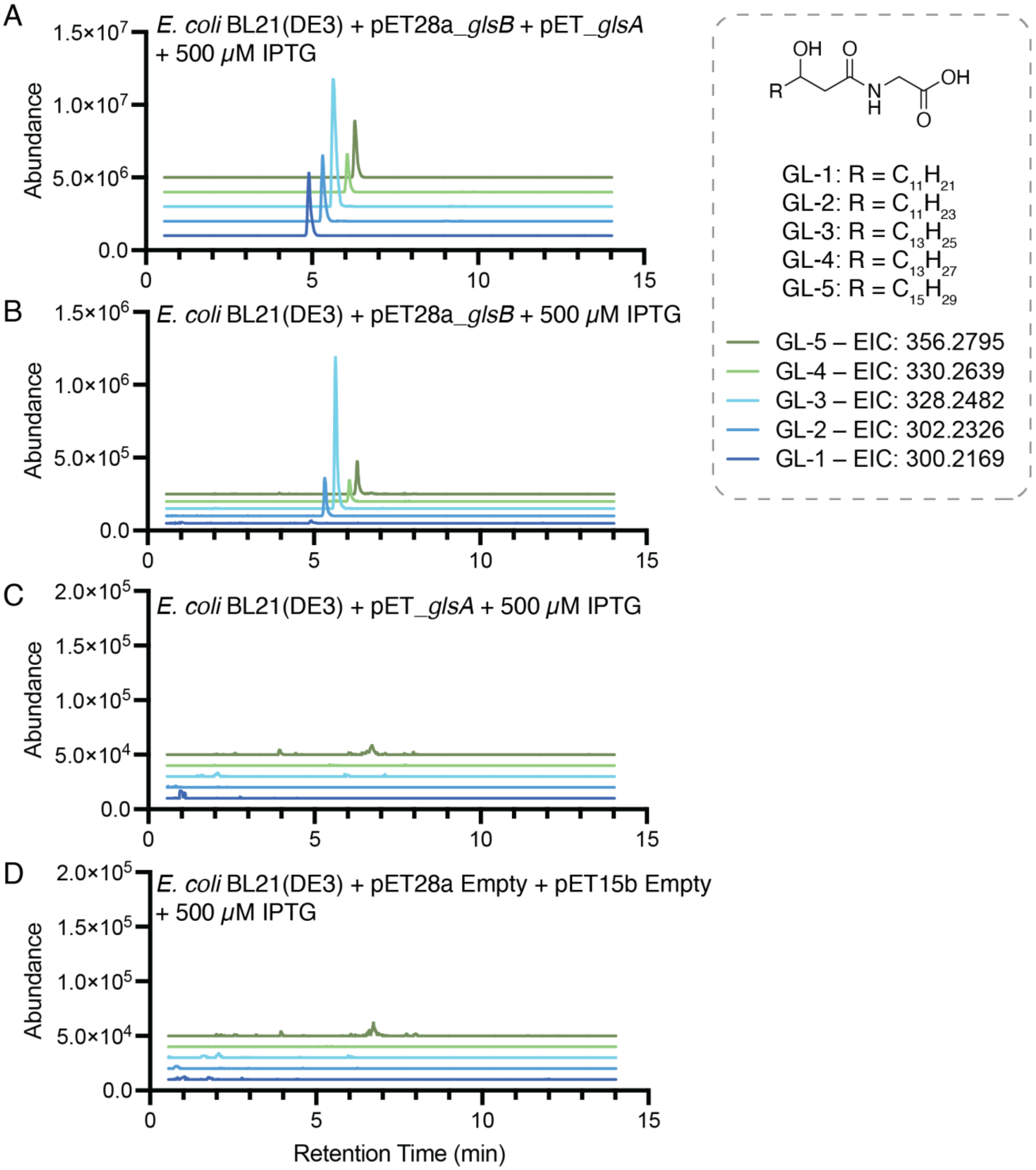

Figure S28. Extracted ion chromatograms (EICs, $[M+H]^+$) of *bis*-GLs produced in *E. coli* BL21(DE3) harboring vectors expressing (A) *glsB* and *glsA,* (B) *glsB*, (C) *glsA*, or (D) empty vectors control. Each culture was treated with 500 µM IPTG.

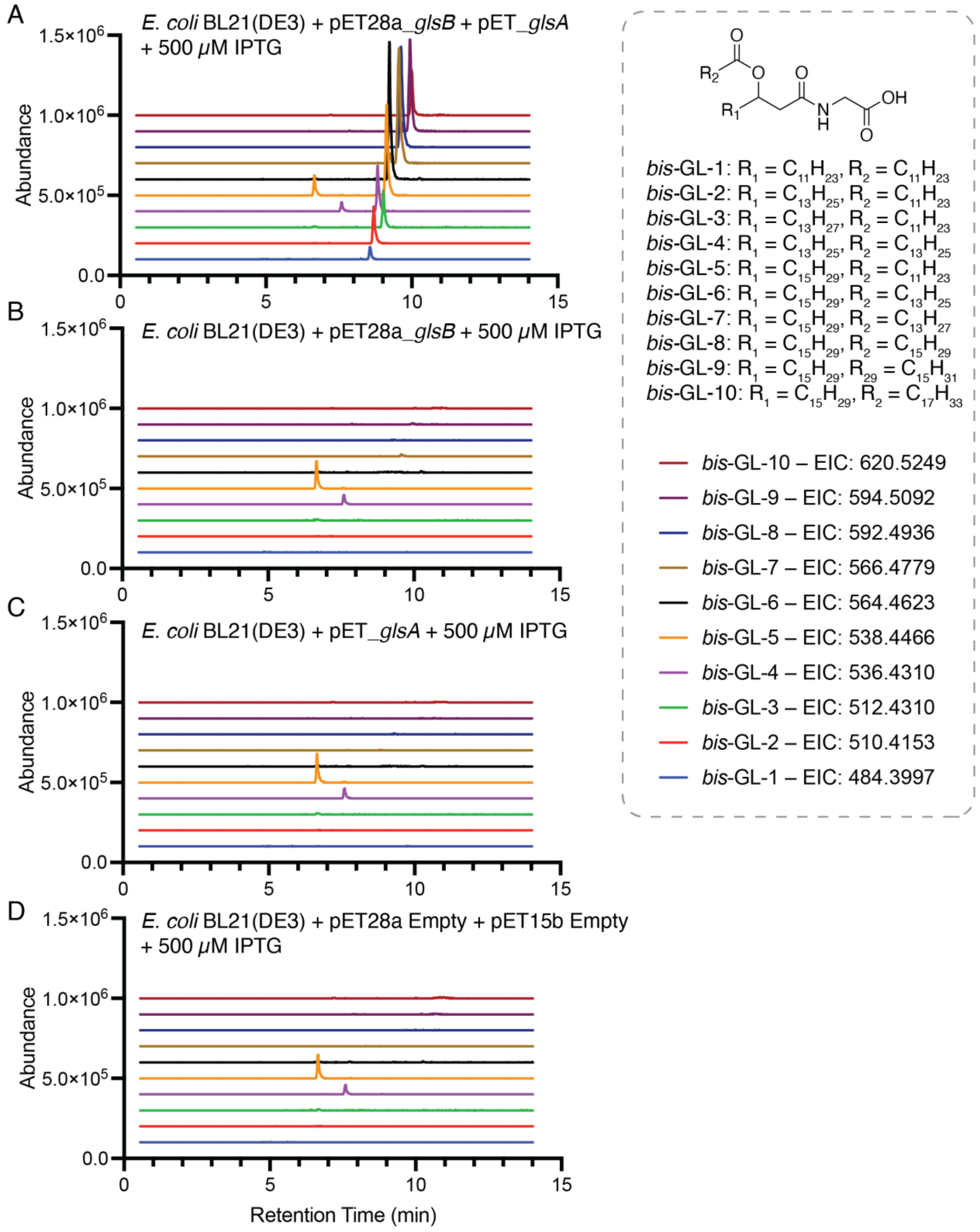

Figure S29. HR-MS/MS data for heterologous GLs 1–5 (A–E) and the predicted fragmentation patterns.

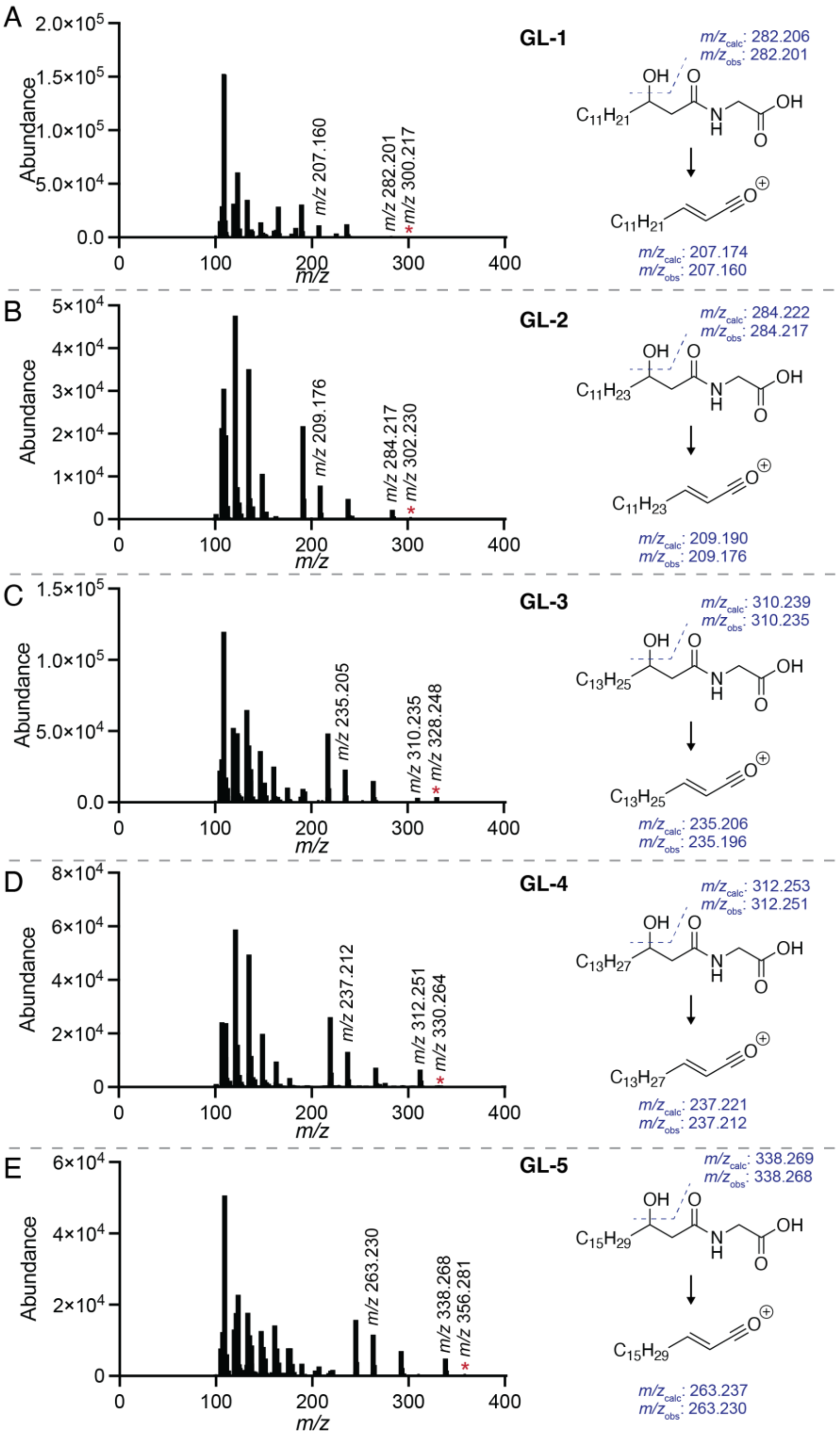

Figure S30. HR-MS/MS data for heterologous *bis*-GLs 1–10 (A–J) and the predicted fragmentation patterns.

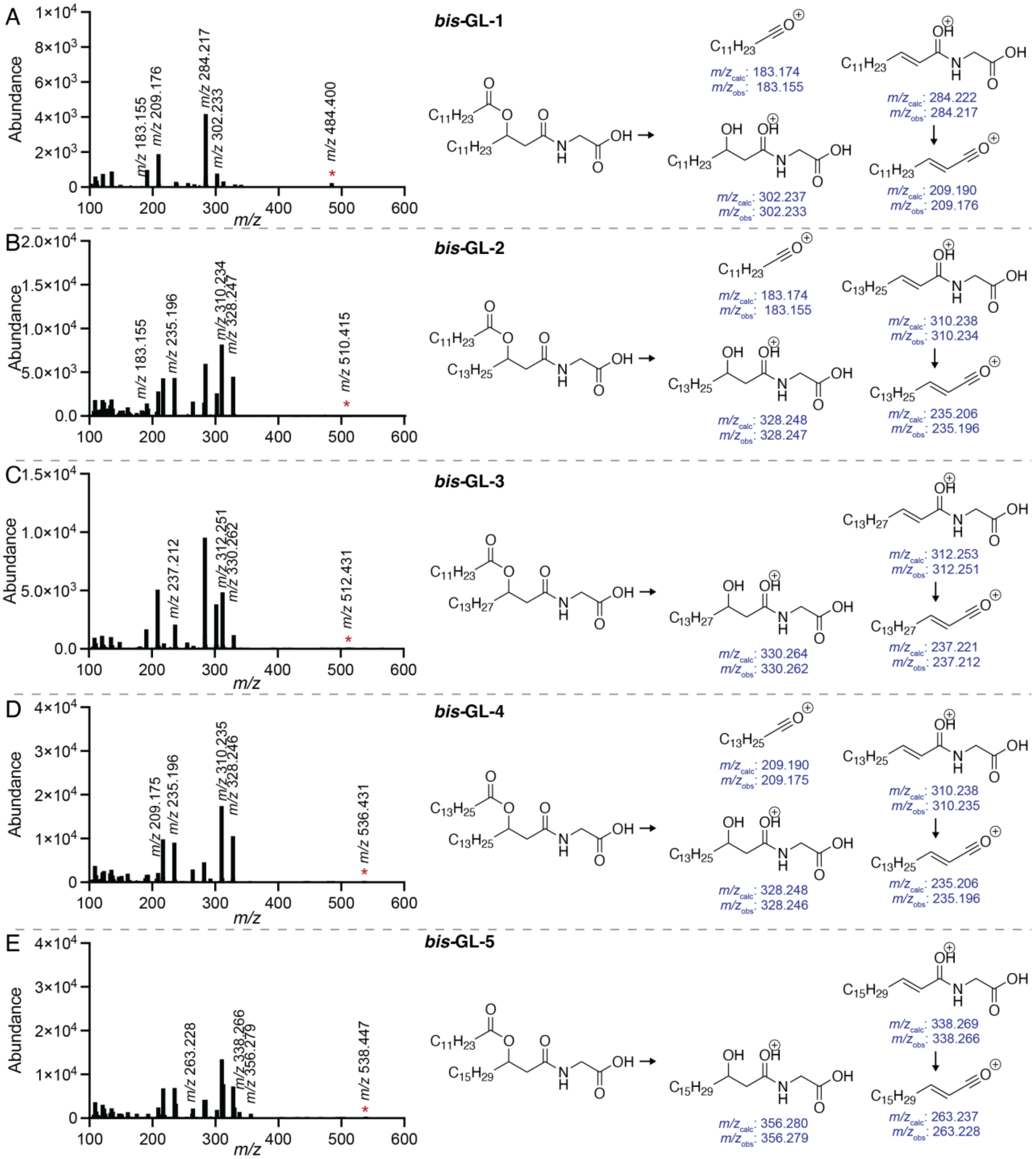

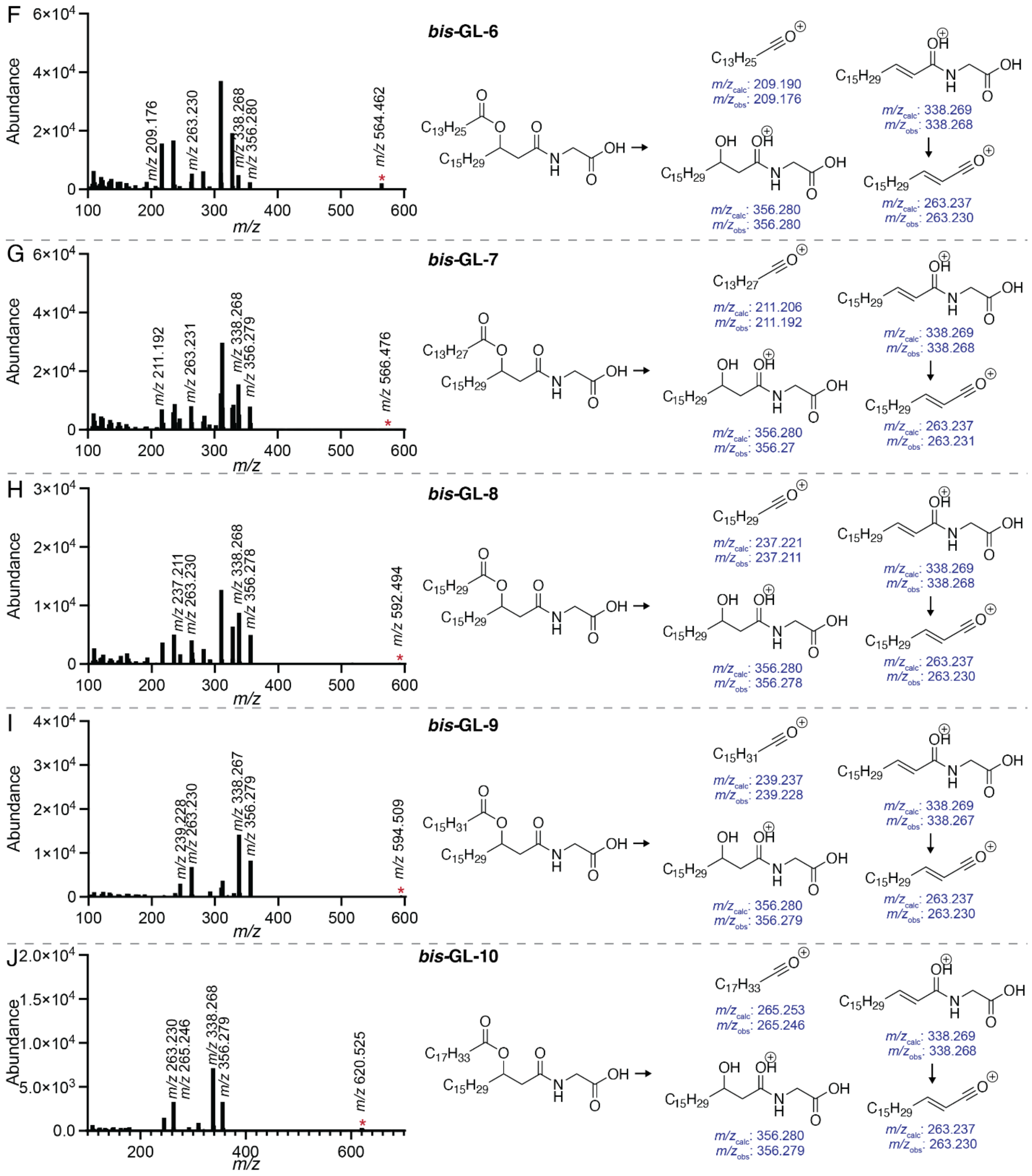

F
G
H
I
J
Abundance
m/z
bis-GL-6
m/z 209.176
m/z 263.230
m/z 338.268
m/z 356.280
m/z 564.462
m/z_calc: 209.190
m/z_obs: 209.176
m/z_calc: 356.280
m/z_obs: 356.280
m/z_calc: 338.269
m/z_obs: 338.268
m/z_calc: 263.237
m/z_obs: 263.230
bis-GL-7
m/z 211.192
m/z 263.231
m/z 338.268
m/z 356.279
m/z 566.476
m/z_calc: 211.206
m/z_obs: 211.192
m/z_calc: 356.280
m/z_obs: 356.27
m/z_calc: 338.269
m/z_obs: 338.268
m/z_calc: 263.237
m/z_obs: 263.231
bis-GL-8
m/z 237.211
m/z 263.230
m/z 338.268
m/z 356.278
m/z 592.494
m/z_calc: 237.221
m/z_obs: 237.211
m/z_calc: 356.280
m/z_obs: 356.278
m/z_calc: 338.269
m/z_obs: 338.268
m/z_calc: 263.237
m/z_obs: 263.230
bis-GL-9
m/z 239.228
m/z 263.230
m/z 338.267
m/z 356.279
m/z 594.509
m/z_calc: 239.237
m/z_obs: 239.228
m/z_calc: 356.280
m/z_obs: 356.279
m/z_calc: 338.269
m/z_obs: 338.267
m/z_calc: 263.237
m/z_obs: 263.230
bis-GL-10
m/z 263.230
m/z 265.246
m/z 338.268
m/z 356.279
m/z 620.525
m/z_calc: 265.253
m/z_obs: 265.246
m/z_calc: 356.280
m/z_obs: 356.279
m/z_calc: 338.269
m/z_obs: 338.268
m/z_calc: 263.237
m/z_obs: 263.230

Figure S31. HR-MS data for the dose-dependent induction of **10–12,** and **14** by DMC in (A–D) *Bacteroides dorei* CL02T12C06 and (E–G) *Bacteroides* sp. 1_1_30. Data were OD_{600nm} normalized and represent the mean ± S.E.M. (n=2-3 biological replicates). *, **, and *** denote difference between control and the indicated condition at p<0.05, p<0.01, and p<0.001, respectively (one-way ANOVA test).

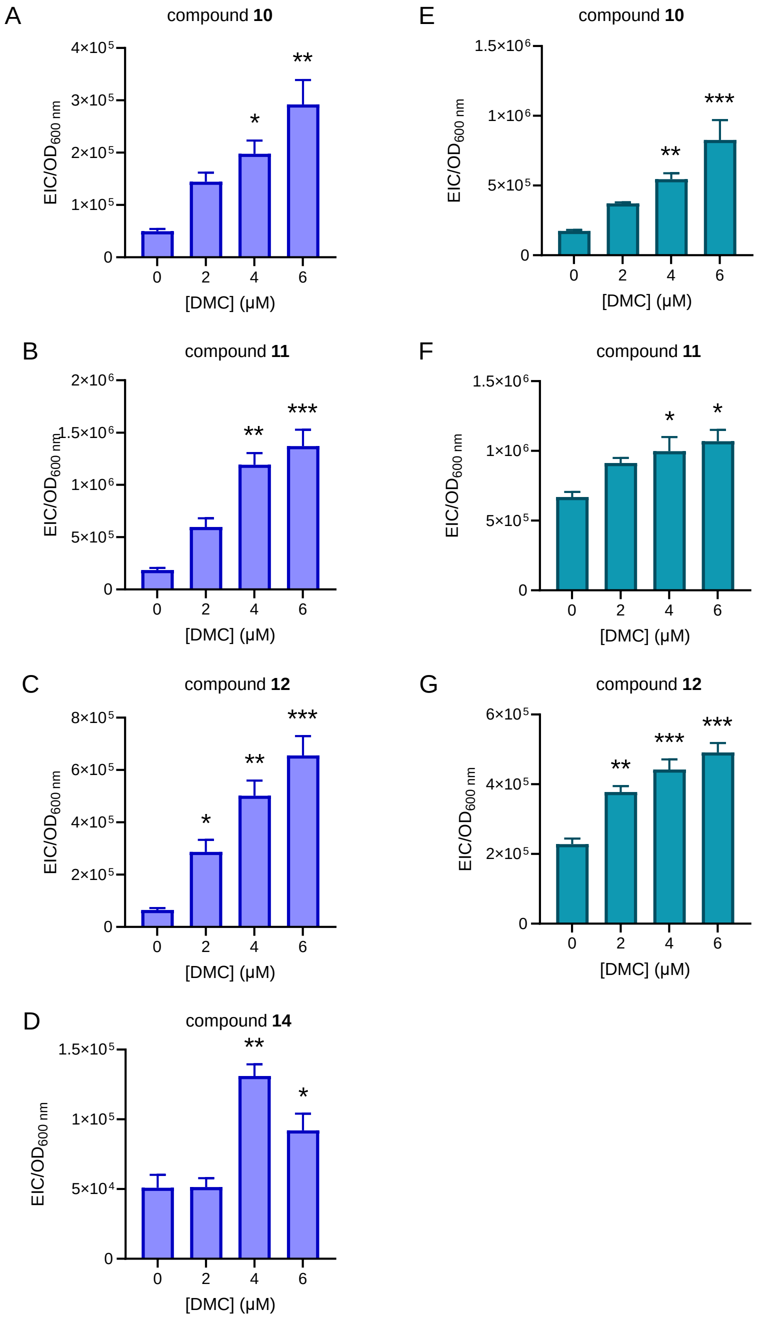

SI References

(1) Covington, B. C.; Seyedsayamdost, M. R. MetEx, a Metabolomics Explorer Application for Natural Product Discovery. *ACS Chem. Biol.* **2021**, *16*, 2825–2833.

(2) Li, X. C.; Ferreira, D.; Ding, Y. Determination of Absolute Configuration of Natural Products: Theoretical Calculation of Electronic Circular Dichroism as a Tool. *Curr. Org. Chem.* **2010**, *14*, 1678–1697.

(3) Li, W.; Godzik, A. Cd-hit: a fast program for clustering and comparing large sets of protein or nucleotide sequences. *Bioinformatics* **2006**, *22*, 1658–1659.

(4) Madeira, F.; Pearce, M.; Tivey, A. R. N.; Basutkar, P.; Lee, J.; Edbali, O.; Madhusoodanan, N.; Kolesnikov, A.; Lopez, R. Search and sequence analysis tools services from EMBL-EBI in 2022. *Nucleic Acids Res.* **2022**, *50*, W276–W279.

(5) Letunic, I.; Bork, P. Interactive Tree Of Life (iTOL) v5: an online tool for phylogenetic tree display and annotation. *Nucleic Acids Res.* **2021**, *49*, W293–W296.

(6) Tietz, J. I.; Schwalen, C. J.; Patel, P. S.; Maxson, T.; Blair, P. M.; Tai, H. C.; Zakai, U. I.; Mitchell, D. A. A new genome-mining tool redefines the lasso peptide biosynthetic landscape. *Nat. Chem. Biol.* **2017**, *13*, 470–478.

(7) Park, P. H.; McMullen, M. R.; Huang, H.; Thakur, V.; Nagy, L. E. Short-term treatment of RAW264.7 macrophages with adiponectin increases tumor necrosis factor-alpha (TNF-alpha) expression via ERK1/2 activation and Egr-1 expression: role of TNF-alpha in adiponectin-stimulated interleukin-10 production. *J Biol. Chem.* **2007**, *282*, 21695–21703.

(8) Wu, Y. Y.; Chen, C. C.; Chyau, C. C.; Chung, S. Y.; Liu, Y. W. Modulation of inflammation-related genes of polysaccharides fractionated from mycelia of medicinal basidiomycete Antrodia camphorata. *Acta Pharmacol. Sin.* **2007**, *28*, 258–267.

(9) Wang, G.; Petzke, M. M.; Iyer, R.; Wu, H.; Schwartz, I. Pattern of proinflammatory cytokine induction in RAW264.7 mouse macrophages is identical for virulent and attenuated Borrelia burgdorferi. *J. Immunol.* **2008**, *180*, 8306–8315.
